# SDA: a data-driven algorithm that detects functional states applied to the EEG of Guhyasamaja meditation

**DOI:** 10.3389/fninf.2023.1301718

**Published:** 2024-01-29

**Authors:** Ekaterina Mikhaylets, Alexandra M. Razorenova, Vsevolod Chernyshev, Nikolay Syrov, Lev Yakovlev, Julia Boytsova, Elena Kokurina, Yulia Zhironkina, Svyatoslav Medvedev, Alexander Kaplan

**Affiliations:** ^1^Faculty of Computer Science, Faculty of Economic Sciences, HSE University, Moscow, Russia; ^2^Center for Neurocognitive Research (MEG Center), Moscow State University of Psychology and Education, Moscow, Russia; ^3^Baltic Center for Neurotechnology and Artificial Intelligence, Immanuel Kant Baltic Federal University, Kaliningrad, Russia; ^4^Academician Natalya Bekhtereva Foundation, St. Petersburg, Russia; ^5^Save Tibet Foundation, Moscow, Russia; ^6^Laboratory for Neurophysiology and Neuro-Computer Interfaces, Lomonosov Moscow State University, Moscow, Russia

**Keywords:** EEG, clustering, unsupervised data annotation, information value, meditation practice, Ward's method, functional states, change point detection

## Abstract

The study presents a novel approach designed to detect time-continuous states in time-series data, called the State-Detecting Algorithm (SDA). The SDA operates on unlabeled data and detects optimal change-points among intrinsic functional states in time-series data based on an ensemble of Ward's hierarchical clustering with time-connectivity constraint. The algorithm chooses the best number of states and optimal state boundaries, maximizing clustering quality metrics. We also introduce a series of methods to estimate the performance and confidence of the SDA when the ground truth annotation is unavailable. These include information value analysis, paired statistical tests, and predictive modeling analysis. The SDA was validated on EEG recordings of Guhyasamaja meditation practice with a strict staged protocol performed by three experienced Buddhist practitioners in an ecological setup. The SDA used neurophysiological descriptors as inputs, including PSD, power indices, coherence, and PLV. *Post-hoc* analysis of the obtained EEG states revealed significant differences compared to the baseline and neighboring states. The SDA was found to be stable with respect to state order organization and showed poor clustering quality metrics and no statistical significance between states when applied to randomly shuffled epochs (i.e., surrogate subject data used as controls). The SDA can be considered a general data-driven approach that detects hidden functional states associated with the mental processes evolving during meditation or other ongoing mental and cognitive processes.

## 1 Introduction

The acquisition of psychological comfort by a person is the basis of any spiritual practice, including religious practices. In this regard, phenomena such as meditation, mind-wandering, insight, and religious excitement can be considered as self-induced changes in the functional state of the brain. Tracking these changes has recently become a popular topic in neuroscience.

A convincing corpus of literature report neurophysiological changes during meditative practices (Lutz et al., 2004; Britton et al., 2014; Lee et al., 2018; Brandmeyer et al., 2019; Volodina et al., 2021; Medvedev et al., 2022). At the same time, there is a high variability of the reported effects that accompany meditative states (Fell et al., 2010; Kaur and Singh, 2015; Lee et al., 2018; Brandmeyer et al., 2019), and this prevents the formulation of a theoretical description of ongoing neurophysiological modulations induced by meditative practices. The variability observed in the literature is explained by the diversity of the content of meditation practices (focused attention, active visualization, open observation, etc.), recording conditions (laboratory, retreat, and monastery), experience of the subjects (time spent in practice), and individual variability (Thomas and Cohen, 2014; Volodina et al., 2021).

EEG dynamics of meditative states were addressed in recent studies. Denison presented a visual analysis of EEG recorded during a progressive meditative sequence toward deeper states of serenity (Dennison, 2019; Fell et al., 2019). Huang and Lo compared EEG recordings of Zen meditation performed by experienced subjects vs. the resting state of the control group (Huang and Lo, 2009). The PSD data at the beginning, at the middle, and at the end of 40-min recordings were compared. A similar setup was applied for Vipassana meditation in a group of Buddhist monks (Marasinghe et al., 2021). The 40-min meditation session was split into 5-min intervals, and PSDs were statistically compared with the beginning of the meditation.

These naïve approaches suffer from a series of limitations. First of all, the choice of the control condition (resting state or beginning of the meditation) is debatable as the arbitrary chosen time intervals of the interest are hard to justify. Second, the approach takes no account of the individual meditative patterns of practitioners (difference in concentration, time of transition to meditative state), which are lost in the averaging procedure and fixed meditation length. Finally, most works considered meditative protocols that are not supposed to have any sequential structure, making the interpretation of EEG dynamics more challenging. Moreover, notice that just a small subsample of the data was analyzed (only the beginning and the end of recordings).

Recently, Volodina et al. (2021) used a more complicated experimental paradigm. Two groups, experienced practitioners and novices, were EEG-recorded when passing through the guided Taoist meditation with 16 audible instructions. Authors compared dynamics of the PSD indices averaged within six formally defined states: (1) control pre-meditation resting state, (2) relaxation and focus on the body, (3) mental silence, (4) active visualization, (5) closing practices, and (6) control post-meditation resting state. The main funding was the two opposite trends to relax and concentrate within the “experienced” group, associated neither with demographic nor with meditation experience. This is evidence that between-subject variability cannot be overcome using stricter experimental conditions (instructions, unified meditation protocol, and time limits). Thus, there is a need for a method that can operate on the data with minimal neurophysiological assumptions on EEG feature dynamics and detect trends and functional state changes within ongoing EEG.

In this study, we present the first attempt to algorithmically detect functional state changes in continuous EEG recording of meditative practice. We assume that EEG records of the ongoing Guhyasamaja meditation performed by highly experienced practitioners in natural conditions (Tibetan monastery) can have intrinsic non-stationary structure that may reflect functional states associated with a particular stage of meditation. This statement is motivated by the predetermined structure of the Guhyasamaja Tantric meditative practice, which includes eight stages of “dissolution of bodily elements and mental states” to the state of “clear light,” the reflection of which we expect to find in the EEG recording.

An unsupervised clustering approach may be in use for such a unique dataset with no strict assumptions on the number of states and state characteristics, see Dai et al. (2018) and Dai et al. (2022) for clustering methods review. The general pipeline for clustering algorithm application is to split continuous EEG data into epochs (points), represent each epoch in the chosen feature space, and then apply the clustering algorithm.

Surprisingly, there is a lack of methods that assist the research of hidden functional states within ongoing EEG. The validation and clustering quality estimation are typically performed the same way as for classification problems by comparing the obtained cluster structure with Ground Truth (GT) labeling provided with the dataset (fixed number of clusters). There are a relatively small number of works where an arbitrary number of clusters is considered or where the most descriptive analysis of features is introduced (Geva and Kerem, 1998; Kazemi et al., 2022); still, these works appeal to the known states (sleep stages and epileptic episodes).

This study introduces the State Detecting Algorithm (SDA), a novel technique addressing the challenge of ongoing EEG clustering without available annotation and minimal assumptions about states or feature behavior. SDA, based on Ward's hierarchical clustering with a temporal connectivity constraint, aims to find change points in time-series data, revealing hidden functional states.

We applied the SDA to EEG data recorded during ongoing Guhyasamaja Tantric meditation and surrogate EEG data without any temporal organization as control. We used common EEG features (PSD, PLV, and coherence index) as SDA input to provide a comprehensive description of detected states. The obtained functional states were assessed by clustering quality metrics, statistical tests between states, and the accuracy of predictive classification models and are compared for meditation data and control. We also introduce an information value (IV) metric to obtain the most descriptive EEG features of each obtained state.

This study presents the SDA approach, along with a comprehensive methodology to estimate the clustering quality without GT annotation. It offers a novel solution for unsupervised clustering in meditation research, contributing to the understanding of ongoing EEG dynamics. The SDA, along with IV analysis, can be used in clinical applications with strict problem formulation, such as sleep stages annotation, as well as in phenomenological psychophysiology research (self-induced functional state changes). The SDA applicability extends to arbitrary time-series data characterized by concealed staged dynamics.

## 2 Methods

### 2.1 Dataset

#### 2.1.1 Meditation protocol

Guhyasamaja Tantric meditation practice follows a strict protocol. Its essential part is the so-called “bringing dharmakaya of death into the path” (tib. འཆི་བ་ཆོས་སྐུའི་ལམ་འཁྱེར།, ‘chi ba chos sku'i lam ‘khyer), which consists of eight consequent stages of “dissolution of bodily elements and mental states” up to the state of “clear light.” The meditation protocol of eight stages is fixed in Buddhist tradition and has been practiced over centuries.

The duration of each stage is not limited, nor is the total meditation duration. Some monks include additional meditative techniques to the meditative session, such as analytical or focused meditation at the beginning of Tantric meditation to initialize the process. Some extend the meditation by performing the Guhyasamaja practice in its entirety, which includes an additional imaginative process of “self-generation” that passes through eight stages of the dissolution process in reverse order. This leads to individual variability of time spent in meditation and an uncertain number of meditative states within the practice. During the experiment, there were no external cues or instructions, and the ongoing meditation was naturally controlled only by a practitioner.

#### 2.1.2 Participants

The participants of the experiment were 30 Tibetan Buddhist monks who had practiced Guhyasamaja Tantric meditation on a regular basis for decades. We use the following notation to refer to the aforementioned EEG recordings: Subj1, Subj2, and so on, up to Subj30, respectively. Participants' meditations were recorded on different days, and the time of the meditation recording was chosen by a practitioner according to his daily routines. The participants signed the informed consent before the experiment. Each participant reported that the meditation was successful (i.e., passing through all eight states of Guhyasamaja meditation) during the questionnaire followed after the practice. During the questionnaire, the practitioners were also asked to subjectively estimate the total time spent in meditation, but the reported time in most cases differed dramatically, up to twice, from the registered time, which indicates that practitioners tend to lose their sense of time during meditation.

For Subj1 23.02.2020-15:30, recording duration 935 s (~16 min), reported duration 30 min.

For Subj2 28.02.2020-7:00, recording duration 2,344 s (~39 min), reported duration 25 min.

For Subj3 01.03.2020-9:00, recording duration 1,302 s (~22 min), reported duration 17 min.

A table with the date, time, and duration of all 30 meditations can be found in Supplementary Table 1.

#### 2.1.3 EEG recording and preprocessing

To provide environmental validity, the experiment took place in a Tibetan monastery in the very room used by participants for meditative practices.

The EEG signals were recorded with the NVX-52 acquisition system (Medical Computer Systems Ltd., Moscow, Russia) at a 500-Hz sampling rate, with analog bandpass filtering between 0.1 and 200 Hz. A digital 50-Hz notch filter was applied to remove artifacts caused by power line noise. Forty EEG channels positioned according to the 10–20 system were used with Cz as a reference electrode.

**Figure 1:**
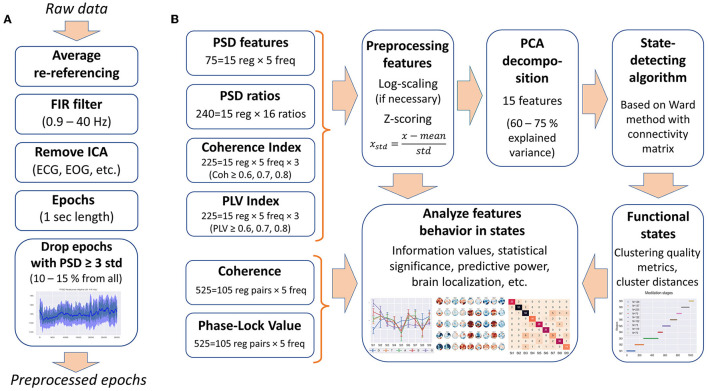
Data processing. **(A)** Steps of the EEG data preprocessing. **(B)** Types of EEG features used in the analysis and feature processing scheme.

The following data processing was performed with MNE-python (Gramfort et al., 2013). The EEG signals were re-referenced to average reference. A 0.9–40-Hz bandpass filter was applied to remove low-frequency artifacts (breath movements and electro-dermal activity) and high-frequency muscle activities (Widmann et al., 2015). Independent component analysis was performed, and ICA components corresponding to artifacts, such as eye blinks, saccades, muscle activities, heart rate, and rheogram, were excluded if present. For further analysis, the EEG data were split into 1-s epochs. Figure 1A shows the preprocessing steps.

Since most clustering techniques are sensitive to outliers, we included an additional step of cleaning the data from artifacts—epochs in which the PSD value exceeded three standard deviations for at least one of the calculated 75 PSDs were rejected from the analysis (EEG features are described in the Section 2.2.1). Thus, ~10–15% of all epochs were removed from each EEG recording.

#### 2.1.4 Simulated data

For each of the three subjects, we randomly shuffled epochs (1-s time windows) within the EEG record to test the SDA performance on the very same data with no temporal structure. The shuffled EEG recordings are referred to below as Subj1_surrogate, Subj2_surrogate, and Subj3_surrogate, respectively, and are called surrogate EEG data in this study. EEG features in the surrogate data were obtained by shuffling feature values calculated for each epoch in the original EEG recordings in the same order as their epoch indices. This allowed us to destroy possible temporal structures in a given feature space.

To verify the stability of functional states obtained with the SDA, we constructed simulated EEG data by randomly rearranging the obtained functional states for each of the three practitioners Subj1, Subj2, and Subj3 and applied the SDA again to test its ability to capture the rearranged states.

The process of generating the EEG data with rearranged states consists of the following steps. We applied the SDA to the initial subject's EEG data and randomly shuffled the obtained functional state indices. Then, we rearranged time-continuous epoch sequences corresponding to these functional states in the same order as their indices. EEG features in the simulated data were obtained by reordering the values of the original features, calculated within each epoch, in the same order as the corresponding epochs.

### 2.2 EEG features

#### 2.2.1 Feature engineering

##### 2.2.1.1 Bands for averaging features

Frequential features were computed for each 1-s epoch for each of the 38 channels (ear electrodes A1 and A2 were excluded from the analysis). Then, data were averaged in the frequency domain within five conventional bands: delta (0.9–4 Hz), theta (4–8 Hz), alpha (8–14 Hz), beta (14–25 Hz), and gamma (25–40 Hz).

**Figure 2:**
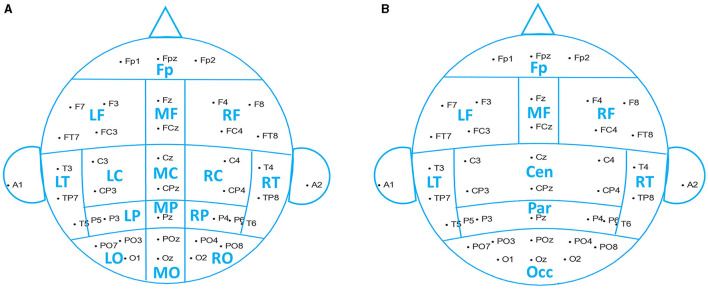
Reduction scheme into spatial ROIs. **(A)** Fifteen brain regions used in data analysis. **(B)** Nine brain regions used for the analysis results demonstration.

Based on the raw signal correlation between channels, we grouped 38 channels into 15 spatial ROIs (region of interest): pre-frontal (Fp1, Fp2, and Fpz), left frontal (F3, F7, FC3, and FT7), midline frontal (Fz and FCz), right frontal (F4, F8, FC4, and FT8), left central (C3 and CP3), midline central (Cz and CPz), right central (C4 and CP4), left temporal (T3, T5, and TP7), right temporal (T4, T6, and TP8), left parietal (P3 and P5), midline parietal (Pz), right parietal (P4 and P6), left occipital (PO3, PO7, and O1), midline occipital (POz and Oz), and right occipital (PO4, PO8, and O2), shown in Figure 2A.

The resulting frequential features were obtained by channel-averaging within each ROI. We used 15 ROIs to perform the data analysis, while for illustrative purposes, we reduced the number of ROIs to 9, merging central, parietal, and occipital regions, as shown in Figure 2B.

We used PSD and PSD ratios, power coherence indices, and phase-locking indices calculated for 1-s epochs as input to the SDA; refer to Supplementary material for the detailed calculation description.

##### 2.2.1.2 PSD features and PSD ratios

EEG power spectral densities were computed using the adaptive multitaper method (Prerau et al., 2017). Seventy-five PSD features were calculated (five frequency bands × 15 brain regions) with the use of MNE-python built-in function [psd_array_multitaper() with option adaptive = True].

We also computed 16 power ratios in 15 brain regions, 240 so-called PSD ratio features in total: theta/delta, alpha/delta, alpha/theta, alpha/(delta+theta), beta/delta, beta/theta, beta/alpha, beta/(delta+theta), beta/(theta+alpha), gamma/delta, gamma/theta, gamma/alpha, gamma/beta, gamma/(delta+theta), gamma/(theta+alpha), and gamma/(alpha+beta) EEG power ratios.

##### 2.2.1.3 Coherence and PLV indices

We computed coherence as a measure of power synchronization between two EEG signals based on cross-power spectral densities (CSD) for each 1-s epoch using a 5-s sliding window over five adjacent epochs centered around the current one. Coherence was calculated for each pair of EEG channels, resulting in 3,515 coherence features (five frequency bands × 703 channel pairs of 38 channels). We also computed phase-locking values (PLV) as a measure of phase synchronization between two signals based on CSD for each 1-s epoch with a 5-s sliding window. The total number of PLV features is 3,515 (five bands × 703 channel pairs), similar to coherence features. Refer to Supplementary material for the exact formulas and details of coherence and PLV calculation.

We did not use coherence and PLV features directly as input to the state-detecting algorithm but used coherence and PLV indices obtained on their basis. The coherence index of EEG channel *x* with threshold *P* is the number of channels *y* and *y*≠*x*, where coherence is greater or equal to the given threshold: [(*Coh*)]_*xy*_≥*P*. Coherence indices were computed for three thresholds (*P*∈{0.6, 0.7, 0.8}) and five frequency bands and were averaged within 15 brain regions, resulting in 255 coherence index features (3 thresholds × 5 bands × 15 regions). By analogy with the coherence indices, PLV indices were calculated, and a total of 255 PLV index features were obtained (3 thresholds × 5 bands × 15 regions) after averaging within brain regions.

The original coherence and PLV features for channel pairs, as far as their analogs for brain region pairs (525 coherence and 525 PLV features for 15 regions, i.e., 5 bands × 105 region pairs), were used as descriptive characteristics in the analysis of the functional states obtained as a result of SDA.

Signal synchronization parameters such as coherence and PLV require a longer time interval than 1 s to be more informative and relevant for physiological interpretation. Experimentally, we found that a 5-s sliding window for calculating coherence and PLV provides an optimal balance between interpretability and the use of 1-s epochs for calculating all features in the study to obtain enough data for effective analysis.

##### 2.2.1.4 Feature processing

Since the underlying Ward's hierarchical clustering method is sensitive to differences in ranges and distribution of data, as well as to feature correlations, the features were preprocessed. The obtained EEG features were log-scaled, if necessary, to approximate the normal distribution (PSDs and PSD ratios), then all the features were *z*-scored (75 PSD features, 240 PSD ratios, 255 coherence indices, and 255 PLV indices, 825 features in total).

To get rid of correlations and to reduce the dimensionality of the feature space, principal component analysis (PCA) was used, as in the study by Vivaldi and Bassi (2006). We restricted the number of principal components to 15 for all EEG recordings to correctly compare inter-cluster distances and clustering quality metrics between the subjects. Fifteen PCA components explain 60–80% of the original feature space variance (depending on EEG recording). Thus, each 1-s epoch was represented by 15 principal components for further use in the SDA. Figure 1B illustrates the steps of feature processing and their use in further analysis in this study.

#### 2.2.2 Information value approach for feature exploration

##### 2.2.2.1 What are IV and WoE

We used the information value (IV) approach to find the most descriptive EEG features for obtained functional states and to estimate the number of important features for each state in comparison with SDA results on surrogate data.

*Information value analysis* is a concept from Information Theory and one of the most useful techniques for selecting important features in a binary classification problem (Good and Osteyee, 2006). It provides a useful framework for exploratory analysis and evaluating variable importance for binary classifiers (Saputra et al., 2023). The problem of finding significant features for each cluster in clustered data (e.g., important neural correlates in given functional states) is similar to a classification problem with binary indicator variables for each cluster, so it can be solved using the information value approach.

The IV is a numerical value that quantifies the predictive power or influence of an independent continuous or categorical variable on the value of a specified binary variable, i.e., measures the importance of a feature. IV calculation is based on the weight of evidence (WoE) coefficients. WoE is closely related to IV and helps to understand if a particular range of values of an independent variable has a higher distribution of events or non-events. The weight of evidence describes the relationship between a predictor and a binary dependent variable, and information value is the measurement of that relationship's power.

**Figure 3:**
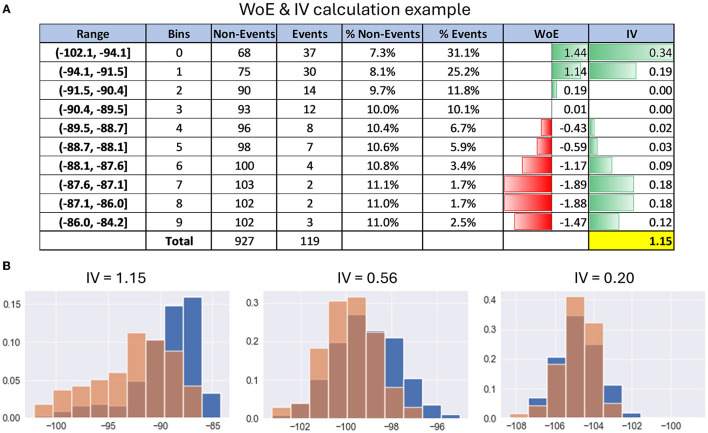
Examples of calculating information value. **(A)** Table with details of the calculation of WoE and IV on the example of PSD feature. **(B)** Histograms with examples of distribution differences of PSD features between the events cluster (red) and the entire dataset (blue) depending on their information value.

Figure 3A gives an example of WoE and IV calculation for better understanding. For exact formulas and calculation details, refer to Supplementary material.

##### 2.2.2.2 Interpretation of IV

In simple terms, the information value shows how much the feature behavior, i.e., distribution in the events subset, differs from its distribution in the entire dataset. Here is a rule of thumb for using information value to understand the predictive power of each neural feature for a given functional cluster of EEG recording:

- IV < 0.2, then the feature is useless for prediction;
- IV varies from 0.2 to 0.4, then the feature has weak predictive power;
- IV from 0.4 to 0.6, then the feature has medium predictive power;
- IV from 0.6 to 1, then the predictive power of the feature is strong;
- IV > 1, very strong predictive power, suspicious behavior (double check it).

IV significance thresholds for EEG features are higher than standard ones, e.g., for credit risk data (Siddiqi, 2006). The reason is that EEG features are not completely independent variables due to their time-series structure, especially in the case of averaging over several adjacent epochs (as for coherence and PLV features).

Figure 3B gives examples of distribution differences in the events class and the entire dataset for EEG features with IV approximately equal to 0.2, 0.6, and 1, respectively.

### 2.3 State-Detecting Algorithm (SDA)

#### 2.3.1 Algorithm idea

The generally accepted clustering approaches are not directly applicable in this study since they do not take into account the requirement of continuity in time of the obtained functional clusters (hypothetically, stages of meditation in our case). Some clustering methods allow the addition of a time-connectivity constraint, that is, the requirement that each cluster can be represented as a connected graph in which neighboring points (i.e., epochs with a distance not exceeding k seconds) are connected by edges. Thus, for data with a non-stationary structure, clusters that are more compact in time dimension can be formed.

**Figure 4:**
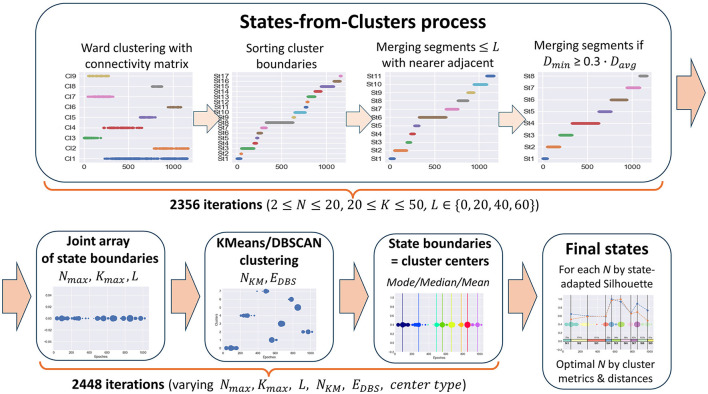
State-Detecting Algorithm (SDA) pipeline.

But even with a connectivity constraint, common clustering methods still do not solve the problem of finding functional states in time-series data since the resulting clusters are generally not continuous in time. Clusters can overlap in the time domain, and some clusters can be inside others or alternate, e.g., one cluster consists of odd epochs and the other one of even epochs. The result of applying Ward's clustering method with a connectivity matrix to meditation data, illustrating the described cluster behavior, is shown in Figure 4 in the first plot of the States-from-Clusters block.

The idea underlying the SDA is to treat the boundaries of clusters obtained with the use of a time-connectivity constraint as potential change points between states since there are no epochs similar to elements of a given cluster before (for left boundary) or after (for right boundary) them at a distance of k seconds. Thus, we sort the array of left and right boundaries of all the clusters in ascending order to receive a partition of the epoch space into time-continuous segments. Then, we improve this partition by merging adjacent segments if they are too short or the inter-cluster distance between them is too small. As a result of the merging process, only the best change-point candidates will stay in the array of potential state boundaries, which separate the segments long enough for the task objective with a sufficiently large cluster distance between them. The described steps of forming states are clearly depicted in the States-from-Clusters block of Figure 4.

The above process provides a partition into states only for a fixed set of clustering parameters, which may not be optimal for a given task. Since the optimal clustering parameters depend essentially on the initial data and are unknown in advance, it is reasonable to repeat the described process of forming potential states from clusters with varying hyperparameters (number of clusters, distance k between epochs in the connectivity constraint, minimum state length, etc.), then combine all the obtained arrays of state boundary candidates into a single sorted array of epoch indices. Repetitions are possible in this array, and the frequency of occurrence of an epoch index in it shows its stability as a potential boundary between functional states.

The next reasonable step is to cluster all elements of the joint array of potential state boundaries by one or more methods with a different clustering principle, e.g., K-Means and DBSCAN, to compare results and choose the best clustering. The centers of obtained clusters are the epoch indices with the best separating ability between potential states in feature space, and we can take them as the resulting state boundaries in the studied EEG data.

The SDA automatically produces the best partition into states based on state-adapted clustering quality measures and initial task restrictions set as algorithm hyperparameters (e.g., minimum state length to avoid the allocation of EEG recording artifacts in a separate state) for each considered number of states, along with corresponding clustering parameters and plots for further expert analysis. The optimal number of final functional states is selected by an expert conclusion as a compromise between the best clustering quality metrics and the conditions of the initial task (required number of clusters, cluster length restrictions, interpretability, required level of detail, etc.).

#### 2.3.2 Ward's method

Among the common clustering methods, a class of agglomerative hierarchical algorithms can add a connectivity constraint due to the principle of the sequential merging of clusters with a possible preliminary check of the connectivity condition. Of all the agglomerative hierarchical methods considered (Ward, average, complete, and single linkage), Ward's method showed the best clustering quality metrics on EEG data; therefore, it was chosen as the basis for the SDA.

A description of Ward's clustering method and the exact formula of the Ward distance (the increase of within-cluster variance after merging two clusters) used in Ward's method can be found in the Supplementary material, as well as in many online tutorials (Contreras and Murtagh, 2015; Virtanen et al., 2020). Ward distance depends on the size of clusters, which is useful in the clustering process to balance cluster sizes during formation, but to analyze the results, we additionally use Centroid distance (Euclidean distance between cluster centers), which is independent of cluster sizes.

Using the connectivity constraint in Ward's method allows us to obtain clusters containing epochs that are close in time, which is necessary to find time-continuous functional states during meditation. We create the connectivity matrix by defining the structure of a connected undirected graph in the epoch space (pairs of 1-s epochs with a distance not exceeding k seconds for a given hyperparameter k are connected by an edge). At each step of the clustering process, the Ward distance between clusters is calculated only if there is an edge between their elements.

#### 2.3.3 Clustering quality measures

When the ground truth of a dataset is not available, we have to use intrinsic methods to assess the clustering quality. In general, intrinsic methods evaluate clustering by examining how well the clusters are separated and how compact the clusters are.

In this study, we use three main clustering quality measures that do not require a priori knowledge of cluster labels, i.e., ground truth (Caliñski and Harabasz, 1974; Davies and Bouldin, 1979; Rousseeuw, 1987). They are the Silhouette coefficient, measuring how similar objects are to their own cluster (cohesion) compared to other clusters (separation) on average (it varies from−1 for incorrect clustering to 1 for highly dense clustering), the Calinski-Harabasz index, also known as Variance Ratio Criterion, characterizing the average density and separation of clusters as the ratio of variance between clusters to variance within clusters (it takes positive values and is higher when clusters are dense and well-separated), and the Davies-Bouldin index, which measures similarity of clusters comparing the distance between clusters with the size of the clusters themselves (zero is the lowest possible score, and values closer to zero indicate a better partition). Refer to Supplementary material for the exact formulas and calculation details.

For assessing the quality of functional state structure obtained with the SDA, we adapted the aforementioned clustering quality measures to better match the specifics of the task. Our goal is to find optimal change-points between different states, that provide the best separation for adjacent time-continuous functional clusters. Good data partition into states means well-separated pairs of adjacent states. Non-adjacent functional states may be similar, and states can alternate, for example.

So, instead of applying the clustering quality measures to the entire dataset partition into functional states, which does not solve the problem, we calculate them on all pairs of adjacent states, considered as two-cluster datasets, and take the average value. In this study, we call these adapted quality measures the state-adapted Silhouette coefficient, Calinski-Harabasz index, and Davies-Bouldin index, and use them to choose the best decision in the state-detecting algorithm.

#### 2.3.4 SDA description

The State-Detecting Algorithm (SDA) consists of two phases.

*The 1st phase* is an iterative process of searching for potential state boundaries based on Ward's hierarchical clustering method with a connectivity matrix. It is repeated for each triple of hyperparameters (*N, K*, and *L*) varying in ranges, as shown in Table 1. Table 1 shows the ranges of values specific to this study for all hyperparameters of the algorithm. Optimal ranges of hyperparameters were chosen empirically based on the clustering quality metrics, the nature of the data, and the purpose of our study.

**Table 1:** SDA hyperparameters.

**Phase**	**Sign**	**Values**	**Description**
Phase 1	*N*	[2, 20]	Number of clusters in the Ward's clustering method
	*K*	[20, 50]	Maximum distance between epochs in Ward's connectivity constraint
	*L*	{0, 20, 40, 60}	Minimum state length in the process of merging segments
	*W*	0.3	Coefficient in the Ward distance requirement for merging segments
Phase 2	*N*_*max*_	{10, 15, 20}	Maximum value of *N* to construct joint array of state boundaries
	*K*_*max*_	{35, 40, 45, 50}	Maximum value of *K* to construct joint array of state boundaries
	*N*_*KM*_	[2, 15]	Number of clusters in KMeans clustering method
	*E*_*DBS*_	{0.02, 0.025, 0.03}	Maximum sample distance in DBSCAN clustering method
	Center type	Mean, Median, Mode	Types of cluster centers in a given clustering

The process of forming potential states is described in the Section 2.3.1 and illustrated in Figure 4, States-from-Clusters block. It consists of the following steps.

1. Ward's clustering is performed for *N* clusters with a connectivity matrix based on *K* neighboring epochs.
2. The array of left and right boundaries of the obtained clusters, sorted in ascending order, forms a partition of the epoch space into time-continuous segments, i.e., potential functional states.
3. Each segment consisting of ≤ *L* epochs is merged with the nearest (by the Ward distance) of the two adjacent segments until only segments whose length exceeds *L* remain (when *L* = 0 this step is skipped).
4. The pair of segments with the smallest Ward distance *D*_*min*_ between them is merged if *D*_*min*_ ≤ *W*. *D*_*avg*_, where *D*_*avg*_ is the average value of Ward distance for all pairs of adjacent segments and *W* is a fixed coefficient. For this study, the coefficient *W* is set to 0.3 (Table 1). The merging process continues until the minimum Ward distance between adjacent segments exceeds the proportion *W* of its average value for all pairs of adjacent segments.

As a result, for each triple (*N, K, L*), we get a partition of the epoch space into potential states with a sufficiently large Ward distance between them. The obtained partition is represented as a sorted array of epoch indices that are state boundary candidates, i.e., change-points between data segments differing in the behavior of features. According to Table 1, there are 19·31·4 = 2, 356 iterations of the 1st phase of the SDA in our study.

*The 2nd phase* is a process of choosing the final state boundaries based on the results of the 1st phase. Its steps are illustrated in Figure 4 after the States-from-Clusters block.

For each triple of hyperparameters (*N*_*max*_, *K*_*max*_, *L*) from Table 1, a subset *Q* of triples (*N, K, L*) is formed, where *N* and *K* vary in ranges [2, *N*_*max*_] and [20, *K*_*max*_], respectively. For each subset *Q*, the following steps are performed.

1. For all triples (*N, K, L*) of the subset *Q*, we combine corresponding arrays of state boundary candidates, obtained in the 1st phase, into a single array of epoch indices, sorted in ascending order, which we treat as the array of state boundary candidates of the final partition.
2. The obtained joint array of epoch indices is clustered with KMeans and DBSCAN methods for all values of hyperparameters *N*_*KM*_ and *Eps*_*DBS*_, shown in Table 1, to reveal dense clusters of array points (coincident or close epoch indices).
3. In each obtained clustering, we select cluster centers of three types (mean, median, and mode) as candidates for the final state boundaries.

Then, for each number of clusters *N*_*KM*_ and for all the other considered hyperparameters (*N*_*max*_, *K*_*max*_, *E*_*DBS*_, *center types*) of the 2nd phase, varying in ranges as shown in Table 1, the best array of final state boundaries is determined based on the maximum state-adapted Silhouette measure (averaged Silhouette coefficient on all pairs of adjacent states).

This measure was chosen because it is the best among all considered clustering measures in determining the right place for the boundary between two adjacent states since it is calculated for each sample in both clusters, and samples classified incorrectly make a negative contribution to it.

As a result, for each number of clusters *N*_*KM*_, a single sorted array of epoch indices remains, which is treated as a set of the final state boundaries for this number of clusters. The optimal number of the resulting functional states (equal to *N*_*KM*_+1) is chosen by an expert conclusion based on the adapted clustering quality metrics (State-adapted Silhouette, Calinski-Harabasz, Davies-Bouldin measures), average distances between adjacent functional states (Ward and Centroid), and the task requirements.

The SDA pipeline was implemented based on the scikit-learn module in the Python library (Vallat and Walker, 2021).

#### 2.3.5 SDA performance evaluation

We applied the SDA to the EEG recordings of meditative practice performed by 30 highly experienced practitioners Subj1–Subj30. This study provides a detailed demonstration of SDA results in three subjects, Subj1, Subj2, and Subj3, respectively, and the average values of the considered quality estimates for all 30 subjects. We also applied the SDA to surrogate EEG data of Subj1_surrogate, Subj2_surrogate, and Subj3_surrogate and compared the results. The idea of such comparison was to analyze and evaluate the SDA performance and quality of the captured functional states within the original EEG of meditative practice compared to the EEG data with poor functional state structure or without it at all.

For the purpose of verifying the stability and separateness of the functional clusters captured by the state-detecting algorithm, as well as the SDA's ability to detect them in a changed order, we rearranged the obtained states for each participant by random reordering of corresponding segments in the EEG recording and applied the algorithm to the rearranged EEG data. The detailed process of reordering the state structure in meditation data is described in the Section 2.1.4 (Simulated data).

Inter-state and overall clustering quality obtained by SDA for subject data and surrogate data were assessed with clustering metrics, paired statistical comparisons with Bonferroni correction, information value (IV) analysis, and predictive models. The details are given below.

##### 2.3.5.1 Clustering quality

In this study, we use five basic clustering measures of the difference between a pair of adjacent functional states in time-series data. These are two types of cluster distances (Ward and Centroid distances) and three types of intrinsic clustering quality measures (Silhouette, Calinski-Harabasz, and Davies-Bouldin scores).

For evaluating the quality of the entire dataset partition into states, we use these measures averaged over all pairs of adjacent states and call them state-adapted clustering quality measures. The description of the aforementioned measures can be found above in the Sections 2.3.2 and 2.3.3. All the five specified measures assess the quality of the obtained partition into functional states and illustrate the mutual arrangement of time-adjacent states in the feature space, i.e., distances between adjacent states, their density and separation (Silhouette and Calinski-Harabasz scores) and similarity level (Davies-Bouldin index).

##### 2.3.5.2 Statistical significance

Statistical significance was estimated for all 1,875 obtained EEG features, averaged within 15 topographic brain regions, including 825 features involved in the SDA (PSDs, PSD ratios, coherence indices, and PLV indices) and 1,050 coherence and PLV features not used directly in the algorithm. The complete list of analyzed EEG features is provided in the Section 2.2.1.

For each subject Subj1, Subj2, and Subj3 and the corresponding surrogate data Subj1_surrogate, Subj2_surrogate, and Subj3_surrogate, statistical differences in EEG features were examined between each pair of states obtained as a result of SDA and for each state in comparison with the median value of the feature. The detailed results of the statistical analysis of the practitioners' data compared with surrogate data are presented in this study and Supplementary material. The same analysis was performed for the remaining 27 subjects (Subj4–Subj30), and the average results are also given in this study.

Since the calculated EEG features are non-normally distributed (even most of the PSD functions with logarithmic scaling did not pass the Shapiro–Wilk normality test) and due to the relatively small size of obtained state clusters, we used the non-parametric Mann–Whitney *U*-test to analyze pairs of states and one-sample Wilcoxon signed-rank test to compare states with the feature general median value. The obtained *p*-values were then corrected for multiple comparisons using the Bonferroni correction procedure as the strictest correction technique (Van der Weele and Mathur, 2019).

##### 2.3.5.3 Information value analysis

We calculated the average IV across all EEG features for subject data compared to surrogates; also, we calculated the proportion of features with medium and strong predictive power (IV ≥ 0.4 threshold) among all features and specific feature groups (five frequency band groups and six type-specific groups of EEG features).

This enabled us to assess the features' predictive power for states detected by the SDA in both cases and to reveal the most important features for each state obtained in the recordings. The description of the information value approach in feature exploration and feature importance analysis is given in detail in the Section 2.2.2.

##### 2.3.5.4 Predictive modeling analysis

Using the partition into functional states obtained by SDA as the target labels, we built predictive classification models to estimate feature prediction power in each state and the SDA's performance expressed in its ability to produce well-separated states in EEG feature space if they are naturally present in the data.

All the models are applied only to PSDs (75 features) and PSD ratios (240 features), described in the Section 2.2.1.2, and used as SDA-input. We did not use coherence and PLV features in predictive models, as well as coherence and PLV indices, because they are calculated for each epoch within a sliding window of five adjacent epochs, and their values in neighboring epochs of train and test datasets may depend on each other causing the risk of overfitting.

We applied three basic classification methods, Support Vector Machine, Logistic Regression, and XGBoost Classifier, as the most stable and well-performing on small datasets with imbalanced data for both multiclass and binary classification tasks with varying sets of hyperparameters. For all the machine learning algorithms, the step of preprocessing input features included filtering important features by their information value (IV ≥ 0.4) for each state in a training dataset in a binary classification task (and taking their union in a multiclass classification task), *z*-scoring, and PCA dimension reduction (20 PCA components left).

In the modeling process, in all cases, test dataset size was taken with 0.4 proportion, stratified *k*-fold cross-validation was used with *k* = 3 due to the small dataset size, and optimal hyperparameters for each classifier were chosen using the grid search process through various combinations of hyperparameters (kernel type and regularization parameter for SVC, solver, penalty type and regularization strength for Logistic Regression and number of estimates, maximum depth, subsample ratio, and learning rate for XGBoost). The best model architecture for each of the classifiers is reported in this study. We assessed the model performance with accuracy and F1 score for a multiclass classification task and balanced accuracy and ROC-AUC score for binary classification.

## 3 Results

### 3.1 Overview

In the current study, we focus on presenting and validating the developed SDA approach, and due to size limitations, a detailed demonstration of its results is provided in the study for three meditation recordings Subj1, Subj2, and Subj3. For the whole dataset (30 EEG recordings), average values of analyzed quality estimates are given within the study, and a detailed information on SDA quality measures for each of the 30 subjects is provided in Supplementary Tables 2, 3.

The SDA produced stable, well-defined functional states for the EEG recordings of all 30 subjects during meditation practice; we specify “well-defined” as statistically different feature dynamics within time-adjacent states. Here, we will discuss three of them in detail—Subj1, Subj2, and Subj3; 9 states were detected within Subj1 recording; 8 states were obtained in Subj2, and 10 states in Subj3. The optimal number of states in each case was chosen based on the local extremum of clustering quality characteristics and the meditative protocol, which assumes at least eight different stages to be traced in the data. The clustering results for surrogate data Subj1_surrogate, Subj2_surrogate, and Subj3_surrogate revealed low-quality metrics, comparable to random partition into states for any number of clusters, so we fixed for them the same number of states as for Subj1, Subj2, and Subj3, respectively, for ease of comparison.

**Figure 5:**
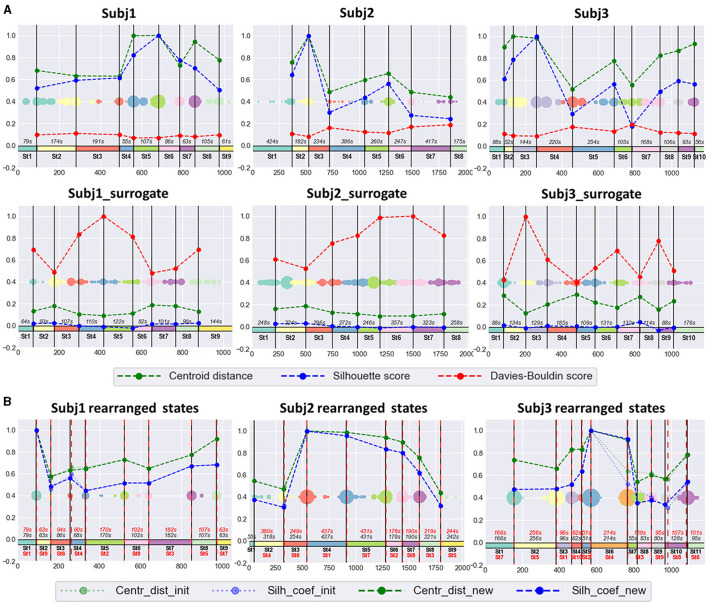
**(A)** Practitioners' and surrogate EEG data partition into states, obtained by SDA. The 1st row contains three EEG recordings of meditation (Subj1, Subj2, and Subj3, respectively), and the 2nd row contains corresponding surrogate data (Subj1_surrogate, Subj2_surrogate, and Subj3_surrogate, respectively). Horizontal axis denotes the epoch index along the recording. Vertical black lines are the final state boundaries obtained as the centers of corresponding clusters shown on a multicolored scatter plot placed in the box center. The scatter plot represents the joint array of state boundary candidates obtained in the SDA 2nd phase; dot size reflects the frequency of occurrence of an epoch in this array. The rectangles with the same colormap along zero-line denote the resulting states. State ID and state total duration (in seconds, s) are shown in black. Vertical axis denotes standardized clustering quality measures (the values in the range [−0.2;1] were obtained by division by the maximum found among pairs of adjacent states over participants' and surrogate data). Dashed polylines show clustering quality measures calculated for each pair of adjacent states (Centroid distance—green line, Silhouette score—blue line, Davies-Bouldin score—red line). **(B)** The SDA results on practitioners' EEG data with rearranged states. There are results of applying SDA after random rearranging of states within the recordings of meditation, depicted in the 1st row of point **(A)** for Subj1, Subj2, and Subj3, respectively. All designations from point **(A)** are valid for the obtained partition into states in point **(B)**, except Davies-Bouldin score polyline, which is not shown in point **(B)**. Additionally, red dashed vertical lines denote the rearranged original state boundaries (the expected boundaries), as well as state IDs and state durations in red color. Dotted light green polyline and dotted light blue polyline denote Centroid distance and Silhouette score, respectively, between the rearranged initial adjacent states (the expected ones). Dashed green polyline and dashed blue polyline denote Centroid distance and Silhouette score, respectively, between the new adjacent states, obtained as a result of repeated application of SDA.

The obtained functional state structure for subjects Subj1, Subj2, and Subj3 and their surrogate data is shown in Figure 5A, correspondingly with an indication of the duration in seconds for each state. It is also presented as a table in Supplementary Table 4. The scatter plots in Figure 5 also illustrate the stability of obtained state boundaries (mainly well-separated and high-density clusters of boundary candidates, mostly concentrated in one point) in practitioners' data in contrast to surrogate data, where we observe mainly spread and poorly separated boundary clusters in the scatter plots. This fact demonstrates a well-defined state structure of the Tantric meditation EEG recordings compared to surrogate data, which is also confirmed by the results of statistical analysis later in the study.

The values of SDA hyperparameters (see Table 1) corresponding to the best partition into states are given in Supplementary material for all six subjects (Supplementary Table 8).

Figure 5B shows the results of the experiment for rearranged Subj1, Subj2, and Subj3 data. In all three cases, SDA captured the initial functional states and rearranged them in a random order, with a slight deviation not exceeding 10 s (see Supplementary Table 6). Tables with clustering characteristics for the obtained state structure and initial state boundaries in the data with randomly rearranged states are given in Supplementary Tables 6, 7.

Further analyses are presented for both subject and surrogate states for contrast.

### 3.2 Clustering metrics comparison

**Figure 6:**
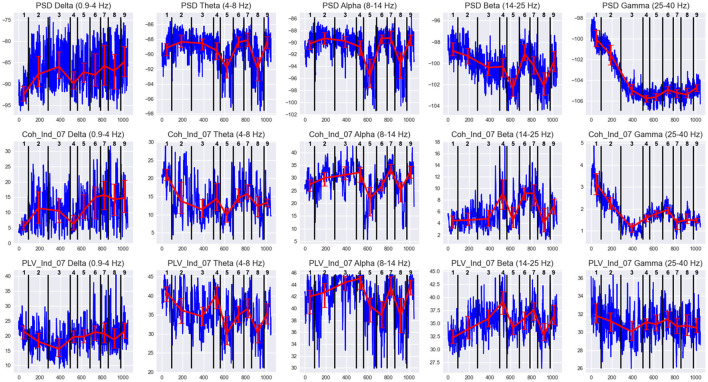
EEG features behavior in states of Subj1's practice recording. Horizontal axis denotes the epoch index along the recording. Vertical black lines are the state boundaries. Black numbers at the top of each plot are the state IDs. Blue lines denote EEG features dynamics along the recording. The features are PSDs (1st row), coherence, and PLV Indices with threshold 0.7 (2nd and 3rd row, respectively) in five frequency bands, averaged over all 38 channels. Red lines represent the Median ± IQR for a selected feature in each state.

The standardized values of the three most representative measures, independent of cluster sizes (Centroid distance, state-adapted Silhouette, and Davies-Bouldin scores), calculated for each pair of adjacent states, are given in Figure 5 for three subjects Subj1, Subj2, and Subj3. For example, from Figure 5, we see that the most distant states in the Subj1 recording are the 5th and the 8th, but the 8th state has more similar epochs with its adjacent states than the 5th. Figure 6 demonstrates these interstate differences in feature space.

Table 2 represents the main clustering parameters (five aforementioned averaged measures for pairs of adjacent states) for the obtained functional state structure of the Buddhist practitioners' recordings and the corresponding surrogate data, detailed for Subj1, Subj2, and Subj3 and averaged for all 30 subjects. We can conclude from Table 2 that all the clustering parameters differ significantly between the two groups of subjects—practitioners and surrogate data (from 4 to 100 times depending on the type of measure). A detailed table with clustering quality measures for each of the 30 subjects can be found in Supplementary Table 2.

**Table 2:** Clustering quality measures of EEG data partition obtained by SDA.

**Subject**	**Number of states**	**Cluster center type for state boundaries**	**Averaged over pairs of adjacent states**				
			**Ward distance**	**Centroid distance**	**Silhouette coefficient**	**Calinski-Harabasz index**	**Davies-Bouldin index**
Subj1	9	Mode	23,293	20.7	0.197	67.5	1.7
Subj2	8	Mode	17,853	12.4	0.102	59.4	2.9
Subj3	10	Mode	11,874	14.7	0.112	32.8	2.7
Subj1_surrogate	9	Median	771	3.6	0.002	1.4	13.2
Subj2_surrogate	8	Median	856	2.5	0.001	1.9	17.1
Subj3_surrogate	10	Mode	942	3.9	0.001	1.7	12.4
Average for 30 subjects Subj1–Subj30			12,509	10.9	0.127	68.7	3.1
Average for surrogate data			856	3.4	0.001	1.7	14.2

Figure 5 also shows that clustering quality measures for each pair of adjacent states in the practitioners' EEG recordings are substantially (several times) better than those in surrogate data (Centroid distance and Silhouette coefficient are higher, and Davies-Bouldin index is lower).

The results of comparing clustering quality measures confirm the presence of a structure of well-separated functional clusters in the EEG recordings of the Buddhist Tantric meditation captured by the SDA, and on the contrary, a poor functional state structure or even absence of it in the surrogate data. A table with the values of all five clustering quality measures on each pair of adjacent states for Subj1, Subj2, and Subj3 and their corresponding surrogate data can be found in Supplementary Table 5.

### 3.3 Statistical comparison

In all cases, SDA detects functional states in the practitioners' meditation recordings with high statistical reliability of differences in neural features. Table 3 for each pair of states shows the proportion of statistically significant features with a confidence level of *p*-value < 0.01 of the number of all EEG features for subjects Subj1 and Subj1_surrogate respectively (Mann-Whitney *U*-test with Bonferroni correction).

**Table 3:** State pairwise statistical significance (A) in practitioner's Subj1 EEG data; (B) in surrogate data Subj1_surrogate.

**(A) Subj1**									
**%**	**St1**	**St2**	**St3**	**St4**	**St5**	**St6**	**St7**	**St8**	**St9**
St1	0	31	57	37	51	45	53	50	45
St2	31	0	32	28	45	39	31	40	22
St3	57	32	0	25	46	42	15	36	10
St4	37	28	25	0	39	31	13	39	11
St5	51	45	46	39	0	39	45	12	33
St6	45	39	42	31	39	0	29	38	26
St7	53	31	15	13	45	29	0	37	8
St8	50	40	36	39	12	38	37	0	23
St9	45	22	10	11	33	26	8	23	0
**(B) Subj1_surrogate**									
**%**	**St1**	**St2**	**St3**	**St4**	**St5**	**St6**	**St7**	**St8**	**St9**
St1	0	0	0	0	0	0	0	0	0
St2	0	0	0	0	0	0	0	0	0
St3	0	0	0	0	0	0	0	0	0
St4	0	0	0	0	0	0	0	0	0
St5	0	0	0	0	0	0	0	0	0
St6	0	0	0	0	0	0	0	0	0
St7	0	0	0	0	0	0	0	0	0
St8	0	0	0	0	0	0	0	0	0
St9	0	0	0	0	0	0	0	0	0

For each pair of states, obtained by SDA, at the intersection of the corresponding row and column, the percentage of significantly different features is indicated according to the Mann–Whitney U-test with Bonferroni correction (p < 0.01).

According to Table 3A, the proportion of statistically significant features between adjacent states (the 2nd diagonal of the matrix) varies from 23 to 39% for the practitioner Subj1, 32% on average, which indicates considerable differences in the behavior of neural features between states and a good ability of the SDA to capture the functional state structure of EEG data. There are no significant features for Subj1_surrogate data, which means the absence of temporal structure. A small number of significant features may be present in surrogate data since the state boundaries are not random but are determined by the SDA, which can detect small fluctuations even in shuffled data.

Analogous tables with similar results for subjects Subj2 and Subj2_surrogate and Subj3 and Subj3_surrogate can be found in Supplementary Tables 10, 11.

The average proportion of statistically significant features between adjacent states for all 30 subjects Subj1–Subj30 varies from 16 to 54% with a mean value of 36% (for detailed information, refer to Supplementary Table 3). On the contrary, for surrogate data, there are no statistically significant features at all.

The Supplementary material section also provides tables with detailed information on feature significance in functional states vs. feature median value for the three subjects Subj1, Subj2, and Subj3 (one-sample Wilcoxon signed-rank test with Bonferroni correction, *p* < 0.01). These tables contain the total proportion of significant features compared to the corresponding surrogate data (the results of the comparison are very close to those for pairs of states—high statistical significance in practitioners' data in contrast to the absence in surrogate data) and the proportion of significant features in a group for different groups of EEG features (five frequency band groups, nine brain region groups, and six feature type groups), which allows us to analyze and compare feature significance in these groups for each state (Supplementary Tables 12–14). The average significance in states versus median value for all 30 subjects varies from 20 to 70% with a mean value of 41% and is given in detail for each of the subjects Subj1–Subj30 in Supplementary Table 3, while for surrogate data, it is zero.

Figure 6 illustrates the differences in feature behavior between the obtained states in Subj1's practice recording. For each state in Figure 6, we can observe essentially different behavior compared to adjacent states of at least one type of feature used as SDA input (PSD, coherence index, and PLV index) in at least one of the five frequency bands. From Figure 6, we can clearly see how these differences in feature behavior were captured by the SDA in the resulting states, and we can analyze the behavior of neural correlates for each state (which could reflect the meditation stage). The analogous figures for practitioners Subj2 and Subj3, as well as for surrogate data Subj1_surrogate, Subj2_surrogate, and Subj3_surrogate, can be found in Supplementary Figures 1–5.

**Figure 7:**
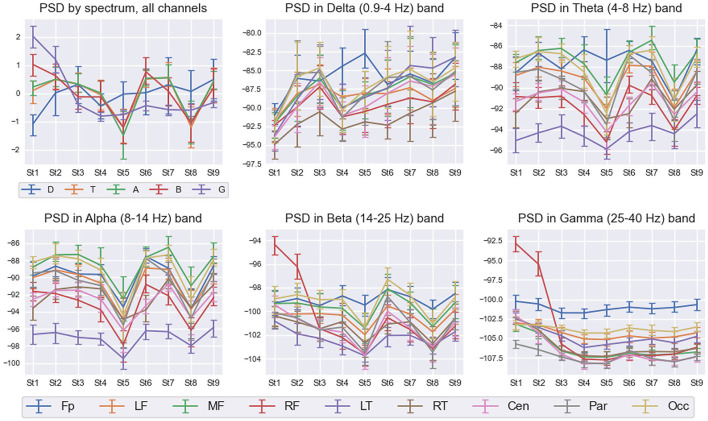
PSD interstate dynamics in five frequency bands and nine spatial ROIs in Subj1's practice recording. Data are represented as Median ± IQR for nine states shown on the horizontal axis. The box in the left upper corner illustrates *z*-scored PSDs averaged over all 38 channels for five frequency bands: delta (D—blue line), theta (T—orange line), alpha (A—green line), beta (B—red line), and gamma (G—purple line). With respect to the five frequency bands, the rest five boxes illustrate the average dynamics of PSDs in nine spatial ROIs: pre-frontal (Fp—blue line), left frontal (LF—orange line), midline frontal (MF—green line), right frontal (RF—red line), left temporal (LT—purple line), right temporal (RT—brown line), central (Cen—pink line), parietal (Par—gray line), and occipital (Occ—khaki line).

Figure 7 shows detailed interstate differences in the behavior of PSD features within five frequency bands over nine spatial ROIs for Subj1. It provides a better understanding of PSD features behavior and their spatial organization in the obtained states. For illustrative purposes, we describe the most essential properties of found states in terms of PSD features for the practitioner Subj1, according to Figure 7. They are also reflected in the statistical significance and information values of these features.

From Figure 7, we can observe high Gamma activity in the right frontal area in the 1st and 2nd states at the beginning and the end of the meditation, which monotonously decreases and stabilizes from the 3rd state. There are also sharp drops of theta, alpha, and beta powers in the 5th and 8th states (for theta, not in all the brain regions; the pre-frontal and left temporal areas have their own trend). Delta activity increases from the 1st state in all the brain areas and peaks in the pre-frontal region in the 5th state.

Statistical plots similar to Figure 7 for all types of EEG features used as input in the SDA (PSD, PSD ratio, coherence index, and PLV index features) can be found in Supplementary material for each of the three practitioners Subj1, Subj2, and Subj3 (Supplementary Figures 6–17).

**Figure 8:**
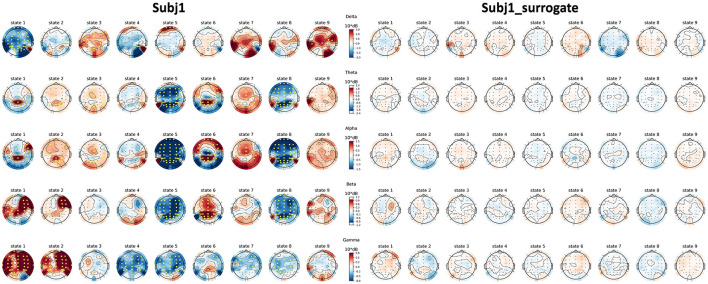
Topographic maps of PSD features for Subj1 and Subj1_surrogate. PSDs are compared within the obtained states of practitioner's and surrogate data (columns) and five frequency bands (rows). Yellow markers denote a significant (*p* < 0.01) difference with the median PSD value of the EEG recording according to the one-sample Wilcoxon signed-rank test, followed by Bonferroni correction.

Topographic maps of PSD features of practitioner Subj1, represented in Figure 8, illustrate their PSD dynamics and localization for all obtained states in the recording and the number and localization of significant features; the same picture is given for Subj1_surrogate data for contrast. Analogous topographic plots for Subj2 vs. Subj2_surrogate and Subj3 vs. Subj3_surrogate are given in Supplementary Figures 18–20.

### 3.4 Information value analysis

Since the functional states obtained by SDA in the original EEG data are well-defined, i.e., differ from each other substantially and have a lot of statistically significant features, we thresholded features with medium and strong predictive power (IV ≥ 0.4) during the IV analysis.

Table 4 represents the detailed IV analysis results for Subj1. Total average IV and proportion of features with IV ≥ 0.4 (features with medium and strong predictive power) are given in comparison with surrogate data Subj1_surrogate. In Table 4 for all the states, we observe the same pattern as in the feature statistical significance analysis. Information values of features are substantially higher for the obtained partition into states of initial meditation recording than for corresponding surrogate data.

**Table 4:** Information value analysis for Subj1 in comparison with Subj1_surrogate.

**EEG data states**	**Subj1_surrogate**			**Subj1**			**Subj1 details**										
	**% of dataset**	**Average IV**	**% with IV** ≥**0.4**	**% of dataset**	**Average IV**	**% with IV** ≥**0.4**	**% of features with IV** ≥**0.4 from all features in group**										
							**Delta**	**Theta**	**Alpha**	**Beta**	**Gamma**	**PSD**	**PSD ratios**	**Coherence**	**PLV**	**Coh index**	**PLV index**
State 1	7	0.07	0	9	0.97	61	67	70	52	58	60	72	79	64	42	74	62
State 2	10	0.06	0	18	0.31	17	16	13	4	15	35	36	22	20	10	20	15
State 3	11	0.06	0	20	0.35	30	19	25	59	19	28	48	40	27	19	37	41
State 4	12	0.04	0	7	0.53	52	58	51	53	57	43	49	50	55	38	60	75
State 5	13	0.04	0	12	0.56	45	21	65	74	34	33	76	61	42	31	55	50
State 6	9	0.06	0	10	0.68	48	17	64	74	49	37	56	50	52	37	63	48
State 7	11	0.05	0	7	0.55	49	39	56	63	51	37	39	41	58	34	69	54
State 8	11	0.05	0	11	0.43	35	15	48	54	29	29	65	48	35	19	36	47
State 9	16	0.04	0	7	0.37	35	43	37	36	29	33	31	30	39	25	53	38

The total average IV for Subj1 varies from 0.3 to 0.97 depending on state, and the proportion of features with IV ≥ 0.4 (which corresponds to sufficiently high predictive power) varies from 17 to 61% from the total number of 1,875 EEG features. In contrast, the average IV for surrogate data Subj1_surrogate does not exceed 0.07, which means the absence of predictive power, and the proportion of important features (IV ≥ 0.4) is zero, i.e., there are no important features at all for obtained states.

Table 4 also provides detailed information on the proportion of important features in different feature groups for Subj1 (five frequency band groups and six type-specific groups of EEG features). This allows us to analyze and compare the importance of different groups of features for obtained states within the EEG recording of meditation. The Supplementary material section contains an extension of Table 4 with the addition of nine brain region groups and analogous results for practitioners Subj2 and Subj3 in comparison with surrogate data Subj2_surrogate and Subj3_surrogate, respectively (Supplementary Tables 15–17).

The total average IV and the proportion of features with IV ≥ 0.4 averaged over states for each of the 30 subjects Subj1–Subj30 can be found in Supplementary Table 3 and varies in the range 0.21–0.83 with an average of 0.44 for IV score and in the range 14–54% with an average of 30% for the proportion of important features. On the contrary, the average IV score for surrogate data does not exceed 0.05, and the proportion of important features is zero.

Feature predictive power based on the IV analysis is confirmed by the quality measures of classification models predicting obtained functional states, which are described in the next section.

### 3.5 Predictive modeling analysis

**Figure 9:**
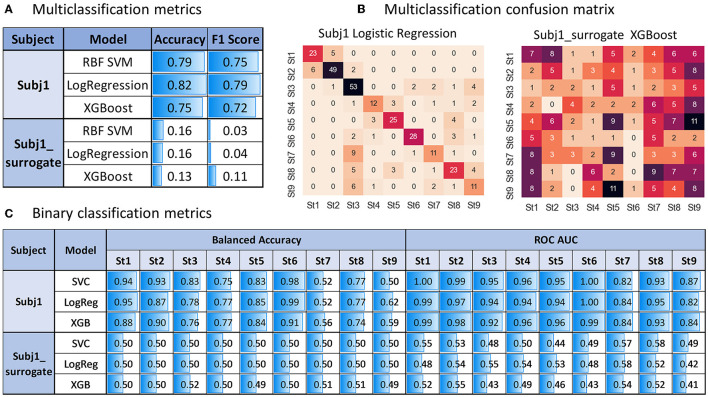
Predictive modeling analysis for Subj1 in comparison with Subj1_surrogate. **(A)** Multiclass classification accuracy and F1 score on test data (stratified sample of size 0.4 of the entire dataset) for three types of classifies predicting nine states produced by the SDA. **(B)** Multiclass confusion matrix for test data using the classifier with the best performance (Logistic Regression for Subj1 and XGBoost for Subj1_surrogate). **(C)** Binary classification balanced accuracy and ROC AUC on test data for three types of classifications predicting each state.

From Figure 9A, we can see sufficiently high accuracy and F1 quality measures of all the models (even based only on PSD-related features, without using coherence and PLV indices) in the multiclass classification task of predicting obtained state structure for practitioner Subj1 (accuracy 0.82 on the best model), pointing to well-separated nine functional states in his meditation recording, captured by SDA, in contrast with surrogate data Subj1_surrogate (accuracy 0.16 on the best model), showing model performance comparable to random choice for all the three classifiers, which points to almost absence of state structure in data.

The confusion matrix in Figure 9B illustrates sufficiently good multiclass classification results for all states of Subj1 recording except for states 7 and 9, in which one-third to one-half of the epochs are misclassified. As we can see from Table 4 with the IV analysis results, states 7 and 9 contain only 7%, that is, about 70 epochs. They are the smallest ones on par with state 4, but they have less important PSD and PSD ratio features (used in the modeling process) than state 4, according to Table 4. These two factors (small size and lack of predictive power) cause underfitting of the classifiers in states 7 and 9 on PSD features, which we can also observe in binary classification results represented in Figure 9C.

All the binary classifiers perform well for all states of Subj1 practice recording except states 7 and 9 (0.79 balanced accuracy and 0.92 ROC AUC on average). All three models corresponding to states 7 and 9 have low balanced accuracy but reasonably high ROC AUC score, which means good enough ranking of probability rates but incorrect choice of threshold for classifying events and non-events due to lack of events in the dataset, which is not compensated by the predictive power of features.

In contrast, all the binary classifiers for all states of surrogate data Subj1_surrogate have balanced accuracy and ROC AUC scores comparable to random choice (0.5 ± 0.02 for balanced accuracy and 0.5 ± 0.1 for ROC AUC), which means poor predictive power of EEG features in states and confirms the absence of state structure in the surrogate data. The analogous tables for Subj2 and Subj3 and their corresponding surrogate data with similar results can be found in Supplementary Figures 21, 22.

**Figure 10:**
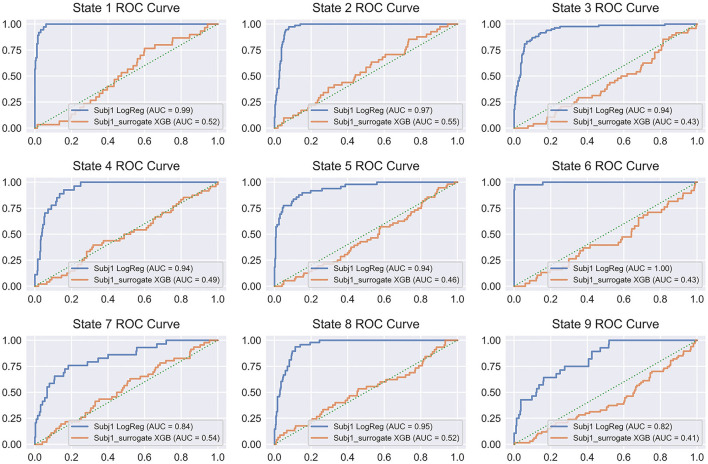
ROC curve plots for binary classifiers, predicting states obtained by SDA for Subj1 and Subj1_surrogate. ROC curves are represented for the classifiers, showing the best average performance (Logistic Regression for Subj1 and XGBoost for Subj1_surrogate). Blue ROC curve corresponds to Subj1, orange one to Subj1_surrogate, and green dotted diagonal line denotes a completely random classifier. The AUC (Area Under Curve) is the area enclosed by the ROC curve. A perfect classifier has AUC = 1, while a completely random classifier has AUC = 0.5.

Figure 10 illustrates the discussed pattern by representing the ROC curves for all the states of Subj1 and Subj1_surrogate for binary classification models, which provide the best average performance in balanced accuracy (Logistic Regression for Subj1 and XGBoost for Subj1_surrogate). From Figure 10, we can conclude that binary classification models for surrogate data Subj1_surrogate, reflected in ROC curves, are close to completely random classification for all the predicted states.

On the other hand, ROC curves for all the states of Subj1′s meditation recording have a clearly defined convex shape, and their ROC-AUC varies from 0.86 to 1 depending on the state, even for PSD-only dataset, which indicates a fairly good predictive power of PSD features in states and therefore well-separated functional states, obtained by SDA. The analogous plots for Subj2 and Subj3, in contrast with Subj2_surrogate and Subj3_surrogate, respectively, demonstrating a similar pattern are represented in Supplementary Figures 23, 24.

Multiclass classification models for all 30 practitioners' meditation recordings demonstrate fairly high performance (whereas they used only PSD-related features, although the SDA also used coherence and PLV indices as input) varying in accuracy from 0.49 to 0.88 with an average of 0.67 and in F1 score from 0.41 to 0.86 with an average of 0.64, as opposed to an average 0.15 accuracy and 0.11 F1 score on the best model for surrogate data, comparable to random choice.

Average binary classifiers performance (on PSD and PSD ratio features only) for all 30 subjects varies in balanced accuracy from 0.6 to 0.91 with a mean value of 0.74 and in ROC AUC score from 0.79 to 0.99 with a mean of 0.91 in contrast with surrogate data demonstrating random classification for all the predicted states (0.5 ± 0.02 for balanced accuracy and 0.5 ± 0.15 for ROC AUC).

Multiclass classifiers and average binary classifier performance metrics for each of the 30 subjects Subj1–Subj30 can be found in Supplementary Table 3.

## 4 Discussion

The goal of this study was to develop an approach for analyzing EEG recordings of the meditative process with eight strictly regulated states in Buddhist monks to check for long-lasting quasi-stationary segments comparable in their number and duration to the stages of the meditative process.

The SDA approach is the initial step in the study of a meditative process with a strictly regulated number of stages, the so-called dissolution process in Buddhism. The neurophysiology of this type of meditation is addressed for the first time in the world.

The main challenge is that monks-subjects cannot give reports on the transition from one stage to another during the meditation process; that is why we introduced SDA as essentially a data-driven technique.

The SDA can be considered as a preprocessing step for further neurophysiological data description and generalization on a sample of more than 30 meditation recordings of monk-subjects recorded in the same paradigm described in the Section 2 with strictly controlled and ecologically valid experimental conditions (staged Guhyasamaja meditation protocol, subject's questionnaire after practice, and EEG recorded in monasteries located in India and Nepal).

The results of the SDA states annotation obtained for the 30-subject sample are given in the Section 3 and Supplementary material sections. The neurophysiological results are planned to be published as a separate article, while in this study, we present the SDA as a method designed specifically to process such a unique dataset and trace Guhyasamaja meditation hidden dynamics on EEG.

### 4.1 SDA performance

We performed a comprehensive initial validation of the proposed algorithm that verified the high sensitivity of SDA and the high quality of detection of functional states in EEG recordings in the presence of a non-stationary structure in a given feature space. The following methods are used in the study to check the quality of the developed algorithm.

#### 4.1.1 SDA performance on rearranged states

To verify the sensitivity of the SDA and stability of obtained functional states in the practitioners' recordings, i.e., potential stages of meditative practice protocol, we randomly rearranged the found states and ensured that SDA stably reproduces them.

The small deviation of found change-points in the rearranged data (< 10 s), see Figure 5B and Supplementary Table 6, confirms the presence of a well-defined stable structure of functional states (presumably, stages of meditation) in the practitioners' EEG recordings, accurately captured by the SDA.

Well-defined state structure of EEG data assumes a significant difference in the feature space between time-adjacent functional states. At the same time, non-neighboring states can be very similar, for example, states can alternate. If two similar functional clusters become adjacent as a result of the rearrangement of the initial states, and their cluster distance is too small, the SDA, applied to the reordered data, can treat them as a single cluster and offer a better partition into states in terms of clustering quality measures underlying the algorithm.

We encounter such a situation in the case of Subj2 and Subj3 with randomly rearranged states, as shown in Figure 5B. The partition of Subj2's rearranged data into nine states, obtained by SDA, accurately captures all the eight reordered initial functional states (and places an additional state boundary), while the partition into eight states differs in one state boundary from the reordered initial one because some similar non-neighboring initial states become adjacent after rearrangement, and the algorithm finds a better partition with slightly higher average Silhouette score and Centroid distance. A similar situation is with Subj3's rearranged data—the partition into 11 states captures all the 10 reordered initial functional states, while the partition into 10 states differs in one state boundary, which provides slightly better average clustering quality metrics. See Figure 5B and Supplementary Tables 6, 7 for details.

#### 4.1.2 SDA performance on real and surrogate EEG data

1. We calculated the Ward and Centroid distances between functional states obtained by the SDA, as well as three main intrinsic (i.e., without ground truth) clustering quality metrics (state-adapted Silhouette, Calinski-Harabasz, and Davies-Bouldin), totally five types of measures, for the original recordings compared with corresponding surrogate EEG with shuffled epochs. We found considerable differences between the original and surrogate data, ranging from 4 to 100 times depending on the type of quality measure (see the Section 3).
2. For each pair of functional states and each state vs. global average, the statistical significance of all EEG features was calculated using the Mann–Whitney and the Wilcoxon criteria with Bonferroni correction for the practitioners' recordings compared to surrogate EEG data. The statistical analysis revealed a considerable amount of significantly different features for the practitioners' recordings and an inconsequential amount for surrogate data.
3. For each functional state, the information values of all EEG features were calculated for both the practitioners and surrogate data, and the comparison results were analyzed. This enabled us to assess the features predictive power for states detected by the SDA in cases of the presence/absence of time-continuous structure within the data and to reveal the most important features for each state obtained in the recordings. The IV analysis also revealed a substantial number of important EEG features for all found states in participants' recordings and an insignificant number of them in the surrogate data.
4. For all the recordings, as well as for the surrogate data, binary classification models were built to predict the occurrence of an epoch in each found state, as well as multiclass models, to predict the entire partition using three methods (SVM, Logistic Regression, and XGBoost classifiers). Then, the analysis and comparison of quality metrics of the classification results were performed to assess the predictive power of EEG features for obtained states of subjects and surrogate data. We got sufficiently high accuracy in predicting functional states within recording for all the practitioners, which confirmed the strong predictive power of EEG features for them and the quality of prediction comparable to random selection for corresponding surrogate data.

To conclude, the State-Detecting Algorithm (SDA) has successfully passed all the listed validations.

### 4.2 Limitations of the study

This study faces certain limitations, with the choice of a suitable control condition being a crucial yet challenging aspect. While options such as repetitive meditation by the same subjects or recording their resting state were considered, there is no guarantee that these conditions will serve as a definitive and proper control. In the case of repetitive meditations, there is a pitfall that each meditative practice is a unique self-induced state; there is no guarantee that the same subject will produce the same number of states with comparative duration and neural characteristics. At the same time, the resting state recording of experienced practitioners could reflect potential biases and may not fully encapsulate the diverse dynamics present in other cognitive states.

Furthermore, establishing a clear connection between the obtained functional states and distinct meditation stages is vital for a comprehensive understanding of the SDA algorithm's applicability. In this study, we present the SDA and its validation on real and surrogate data and a general methodology, how to obtain and analyze the states of an individual EEG recording of Guhyasamaja meditation on an example of 30 subjects (3 monks recordings were taken for an extended demonstration). Our next article will be dedicated to a more extensive exploration and systematization of the diverse states observed in 30 Guhyasamaja meditation recordings. The analysis of the between-monks variability and revealing trends observed in the sample will be the focus of a distinct and forthcoming study.

In addition, we would like to check the SDA performance on more conventional datasets to prove that this approach has the potential for wide application. It is essential to conduct further testing on datasets with publicly available ground truth (GT) labeling, particularly in scenarios such as sleep stages classification. These limitations will also be addressed in the next articles.

## 5 Conclusion

To solve the problem of detecting stages in ongoing EEG recording of Buddhist Tantric Guhyasamaja meditation, i.e., the optimal change-points between different functional states, in the absence of ground truth, we proposed a data-driven unsupervised algorithm based on hierarchical clustering techniques called State-Detecting Algorithm (SDA). To the best of our knowledge, this is considered the first automated unsupervised data-driven method that finds natural time-continuous functional clusters in time-series data, with maximizing differences between time-adjacent clusters in a given feature space.

The performance of the proposed SDA was investigated on Guhyasamaja meditation EEG recordings of 30 Buddhist practitioners in comparison with surrogate data obtained by shuffling epochs of the original EEG recordings.

The dramatic contrast of SDA performance on the meditation practice EEG recordings and surrogate data was observed. *Post-hoc* analyses of the real meditation recordings revealed:

1) Clustering quality metrics of the practitioners' EEG data partition into states are several times higher than the values for surrogate data.
2) Significant statistical differences between time-adjacent states were observed in a substantial amount of EEG features.
3) Information value analysis revealed a considerable number of features with strong predictive power for each state.
4) Supervised ML classification algorithms are easily trained to predict the states obtained by SDA with high accuracy.

In contrast, none of these results were replicated on surrogate data with the lack of temporal structure.

An additional test of the sensitivity of the SDA was carried out, showing that after randomly rearranging the obtained states in practitioners' EEG data, the algorithm stably reproduces them. This indicates not only the high sensitivity and robustness of the SDA but also the presence of a well-defined state structure in the Buddhist practitioners' meditation recordings.

During the study, the most important neural features and their localization in the cerebral cortex were identified for all discovered functional states for each practitioner based on the information value metric, their level of statistical significance, and their contribution to the accuracy of classification models predicting the states.

The SDA developed to detect time-continuous data segments that differ most in the behavior of features may be of use not only for EEG data but also for time-series data of arbitrary nature with a non-stationary temporal structure in a given feature space for capturing and investigating hidden functional states.

Furthermore, we plan to present the results of a comparative analysis of the functional states obtained using the SDA on 30 EEG recordings of Buddhist Tantric Guhyasamaja meditation practice performed by 30 Tibetan monks, reveal common tendencies, and describe the behavior of general neural correlates accompanying the practice.

## Data availability statement

The raw data supporting the conclusions of this article will be made available by the authors, without undue reservation.

## Ethics statement

The studies involving humans were approved by Bekhtereva Foundation University. The studies were conducted in accordance with the local legislation and institutional requirements. The participants provided their written informed consent to participate in this study.

## Author contributions

EM: Conceptualization, Data curation, Formal analysis, Investigation, Methodology, Software, Validation, Visualization, Writing—original draft. AR: Conceptualization, Investigation, Methodology, Project administration, Supervision, Validation, Visualization, Writing—original draft, Writing—review & editing. VC: Conceptualization, Funding acquisition, Project administration, Resources, Supervision, Validation, Methodology, Writing—review & editing. NS: Data curation, Validation, Visualization, Writing—review & editing. LY: Data curation, Validation, Writing—review & editing. JB: Data curation, Writing—review & editing. EK: Data curation, Funding acquisition, Project administration, Resources, Writing—review & editing. YZ: Data curation, Methodology, Project administration, Resources, Writing—review & editing. SM: Conceptualization, Data curation, Funding acquisition, Resources, Supervision, Writing—review & editing. AK: Conceptualization, Data curation, Funding acquisition, Project administration, Resources, Supervision, Validation, Writing—review & editing.
