# Supplementary material for: SDA: a data-driven algorithm that detects functional states applied to the EEG of Guhyasamaja meditation
Source: Front Neuroinform. 2024 Jan 29;17:1301718. doi: 10.3389/fninf.2023.1301718 (PMC10859925; doi:10.3389/fninf.2023.1301718)
Supplement: Supplementary file 1 [file Data_Sheet_1.docx]

**SDA: a data-driven algorithm that detects functional states applied to the EEG of Guhyasamaja meditation**

**Ekaterina Mikhaylets, Alexandra Razorenova*, Vsevolod Chernyshev, Nikolay Syrov, Lev Yakovlev, Julia Boytsova, Elena Kokurina, Yulia Zhironkina, Svyatoslav Medvedev and Alexander Kaplan**

**Supplementary materials**

### **Calculation details**

#### **Coherence and PLV calculation**

*Coherence* is a measure of synchronization between two EEG signals, which estimates similarity of their spectral characteristics per frequency. For a pair of signals $x$ and $y$ coherence is the normalized cross spectrum, varying in the range $[0,1]$ and for a given 1-s epoch and frequency band estimated by formula

$${Coh}_{xy}=\frac{\left| E[S_{xy}] \right|}{\sqrt{E\left[ S_{xx} \right]\cdot E[S_{yy}]}} ,$$

where $S_{xy}$ is the cross-power spectral density (CSD) between $x$ and $y$, $S_{xx}$ and $S_{yy}$ are power spectral densities (PSD) for $x$ and $y$ respectively, $E[ ]$ denotes the arithmetic mean of specified values for 5 adjacent 1-s epochs with the given epoch in the center. Similar to PSD, CSD values were calculated using the adaptive Multitaper method and averaged for each frequency band.

*Phase-locking value (PLV)* is a measure of phase synchronization between a pair of signals $x$ and $y$, varying in the range $[0,1]$ and for a given 1-s epoch and frequency band estimated by

$${PLV}_{xy}=\left| E\left[ \frac{S_{xy}}{\left| S_{xy} \right|} \right] \right| .$$

#### **Information Value and WoE calculation**

For each particular class (range of values) of an independent variable $x$ and dependent binary variable $y$, we can calculate *Weight of evidence* (WoE) as following:

$$WoE=\ln\left( \frac{\%Events}{\%NonEvents} \right) ,$$

where *%Events* is the ratio of the number of events in the certain class of variable $x$ to the number of events in total, and *%NonEvents* is similarly the proportion of non-events.

For categorical variables each value forms a class, and continuous variables are divided into classes by percentile-based binning. In general, 10 bins with deciles of variable $x$ are taken. Ideally, each bin should have at least 10% of observations to be representative taking into account relatively small dataset sizes in EEG analysis.

If a particular class or bin contains no event or non-event, we add 0.5 to the number of events and non-events in a class to ensure that WoE coefficients are defined everywhere. Then *Information value* (IV) of a variable is calculated by formula:

$$IV=\sum_{Bins} \left( \%Events-\%NonEvents \right)\cdot WoE .$$

#### **Ward method description**

*Ward method* is an agglomerative hierarchical clustering method that minimizes variance within clusters. It operates on the following principle. Initially, each element forms a separate cluster. At each step, the two clusters with the smallest Ward distance are combined into one. The process continues until the specified number of clusters is reached or the threshold of the distance between clusters is exceeded.

*Ward distance* between two clusters is calculated as the increase in the “error sum of squares” (ESS) after merging two clusters into a single cluster, where ESS of a cluster is the sum of squares of the deviations from the cluster center (mean vector). After mathematical transformations Ward distance between clusters $X$ and $Y$ is expressed by the formula:

$$D\left( X,Y \right)=ESS\left( X\cup Y \right)-\left( ESS\left( X \right)+ESS\left( Y \right) \right)=\frac{N_{X}\cdot N_{Y}}{N_{X}+N_{Y}}\left\| \boldsymbol{m}_{X}-\boldsymbol{m}_{Y} \right\|^{2} ,$$

where $N_{X}$ and $N_{Y}$ are the sizes, and $\boldsymbol{m}_{X}$ and $\boldsymbol{m}_{Y}$ are the centers (mean vectors) of the clusters $X$ and $Y$ respectively.

*Centroid distance* between two clusters is the Euclidean distance between the centers of these clusters.

In essence, Ward distance is the merging cost of combining two clusters and measures how much the sum of squares will increase when we merge them. In fact, Ward distance is weighted squared Centroid distance, where the weighting coefficients depend on cluster sizes. Thus, Ward distance is sensitive to cluster sizes, which is useful for getting size-balanced clusters during the clustering process.

#### **Clustering quality measures calculation**

*Silhouette coefficient* for a single sample is calculated as $s=\frac{b-a}{max(a,b)}$ where $a$ is average intra-cluster distance, i.e. the average distance between a sample and all other points in the same class, and $b$ is average inter-cluster distance, i.e. the average distance between a sample and all other points in the next nearest cluster. It varies from -1 for incorrect clustering to 1 for highly dense clustering. The Silhouette Coefficient for a set of samples is defined as the mean of the Silhouette Coefficient for each sample.

*Calinski-Harabasz index* is defined as the ratio of the between-clusters dispersion and the within-cluster dispersion:

$$CH=\frac{tr(B_{k})}{tr(W_{k})}\cdot\frac{n-k}{k-1} ,$$

where $n$ is the dataset size, $k$ is the number of clusters, $tr(B_{k})$ is the trace of the between group dispersion matrix and $tr(W_{k})$ is the trace of the within-cluster dispersion matrix. It takes positive values and is higher when clusters are dense and well separated.

*Davies-Bouldin index* is defined as the average similarity between each cluster and its most similar one and is given by formula:

$$DB=\frac{1}{k}\sum_{i=1}^{k} \max_{i\neq j} \frac{s_{i}+s_{j}}{d_{ij}} ,$$

where $k$ is the number of clusters, $s_{i}$ is the average distance between each point of cluster $i$ and the centroid of that cluster (the cluster diameter), $d_{ij}$ is the centroid distance between clusters $i$ and $j$. Zero is the lowest possible score, values closer to zero indicate a better partition.

***Supplementary Table 1.*** *Date-time and duration of meditation practice for all subjects.*

| **Subject** | **Meditation Date** | **Start Time** | **Meditation Duration** |
| --- | --- | --- | --- |
| **Subj1** | 01.03.2020 | 7:06:00 | 16 min |
| **Subj2** | 28.02.2020 | 4:52:00 | 39 min |
| **Subj3** | 29.02.2020 | 12:19:00 | 22 min |
| **Subj4** | 23.02.2020 | 4:18:00 | 26 min |
| **Subj5** | 23.02.2020 | 13:31:00 | 30 min |
| **Subj6** | 26.02.2020 | 7:03:00 | 51 min |
| **Subj7** | 26.02.2020 | 12:29:00 | 18 min |
| **Subj8** | 27.02.2020 | 12:31:00 | 53 min |
| **Subj9** | 27.02.2020 | 7:07:00 | 1 h 24 min |
| **Subj10** | 29.02.2020 | 6:58:00 | 24 min |
| **Subj11** | 02.03.2020 | 7:11:00 | 26 min |
| **Subj12** | 02.03.2020 | 12:28:00 | 1 h 16 min |
| **Subj13** | 25.04.2022 | 8:21:00 | 59 min |
| **Subj14** | 25.04.2022 | 13:29:00 | 22 min |
| **Subj15** | 26.04.2022 | 12:43:00 | 21 min |
| **Subj16** | 27.04.2022 | 8:16:00 | 50 min |
| **Subj17** | 28.04.2022 | 11:51:00 | 39 min |
| **Subj18** | 29.04.2022 | 7:22:00 | 45 min |
| **Subj19** | 10.05.2022 | 14:00:00 | 38 min |
| **Subj20** | 10.05.2022 | 11:51:00 | 23 min |
| **Subj21** | 11.05.2022 | 6:12:00 | 34 min |
| **Subj22** | 11.05.2022 | 14:00:00 | 38 min |
| **Subj23** | 12.05.2022 | 12:17:00 | 30 min |
| **Subj24** | 13.05.2022 | 12:52:00 | 50 min |
| **Subj25** | 13.05.2022 | 15:04:00 | 32 min |
| **Subj26** | 14.05.2022 | 6:25:00 | 30 min |
| **Subj27** | 09.05.2023 | 15:38:00 | 36 min |
| **Subj28** | 11.05.2023 | 15:40:00 | 34 min |
| **Subj29** | 17.05.2023 | 9:09:00 | 17 min |
| **Subj30** | 18.05.2023 | 7:36:00 | 25 min |

***Supplementary Table 2.*** *Clustering quality measures of SDA results for all subjects.*

| **Subject** | **Number of states** | **Cluster center type for state boundaries** | **Averaged over pairs of adjacent states** | | | | |
| --- | --- | --- | --- | --- | --- | --- | --- |
|  |  |  | **Ward distance** | **Centroid distance** | **Silhouette Coefficient** | **Calinski-Harabasz Index** | **Davies-Bouldin Index** |
| **Subj1** | 9 | mode | 23293 | 20.7 | 0.20 | 67.5 | 1.7 |
| **Subj2** | 8 | mode | 17853 | 12.4 | 0.10 | 59.4 | 3.5 |
| **Subj3** | 10 | mode | 11874 | 14.7 | 0.11 | 32.8 | 2.7 |
| **Subj4** | 9 | mode | 4891 | 8.1 | 0.09 | 29.8 | 3.5 |
| **Subj5** | 9 | mode | 13664 | 13.3 | 0.16 | 73.8 | 2.1 |
| **Subj6** | 10 | med | 9051 | 8.2 | 0.11 | 62.1 | 3.3 |
| **Subj7** | 7 | mode | 3962 | 8.1 | 0.08 | 23.6 | 4.2 |
| **Subj8** | 9 | med | 10284 | 8.7 | 0.07 | 42.9 | 4.0 |
| **Subj9** | 12 | med | 8728 | 7.2 | 0.06 | 40.8 | 5.3 |
| **Subj10** | 9 | mode | 4559 | 9.3 | 0.12 | 25.3 | 4.0 |
| **Subj11** | 7 | med | 10803 | 10.5 | 0.16 | 82.7 | 2.4 |
| **Subj12** | 9 | med | 21645 | 8.8 | 0.10 | 127.3 | 3.2 |
| **Subj13** | 12 | med | 13660 | 11.3 | 0.14 | 72.6 | 2.8 |
| **Subj14** | 11 | med | 6768 | 11.5 | 0.15 | 44.3 | 2.2 |
| **Subj15** | 5 | mode | 11127 | 12.4 | 0.17 | 62.5 | 2.8 |
| **Subj16** | 10 | med | 13621 | 13.1 | 0.18 | 61.0 | 4.2 |
| **Subj17** | 8 | med | 9958 | 8.9 | 0.10 | 55.7 | 3.7 |
| **Subj18** | 11 | mode | 9872 | 10.2 | 0.10 | 47.3 | 3.1 |
| **Subj19** | 7 | med | 20497 | 9.7 | 0.12 | 104.0 | 2.9 |
| **Subj20** | 9 | med | 3525 | 7.5 | 0.07 | 20.2 | 3.7 |
| **Subj21** | 9 | mode | 15339 | 13.0 | 0.18 | 90.4 | 2.0 |
| **Subj22** | 8 | med | 15418 | 10.0 | 0.12 | 85.9 | 3.3 |
| **Subj23** | 7 | mode | 16050 | 11.5 | 0.14 | 95.2 | 2.8 |
| **Subj24** | 7 | mode | 23915 | 10.4 | 0.16 | 190.4 | 2.5 |
| **Subj25** | 8 | mode | 4823 | 7.4 | 0.05 | 23.3 | 4.3 |
| **Subj26** | 8 | mode | 15155 | 12.2 | 0.15 | 83.2 | 2.4 |
| **Subj27** | 10 | mode | 14043 | 12.2 | 0.19 | 97.8 | 2.0 |
| **Subj28** | 10 | mode | 12841 | 12.9 | 0.22 | 105.0 | 1.8 |
| **Subj29** | 3 | med | 11986 | 10.5 | 0.11 | 59.4 | 3.2 |
| **Subj30** | 8 | mode | 16050 | 11.5 | 0.14 | 95.2 | 2.8 |

***Supplementary Table 3.*** *Information value, statistical significance, model quality estimates of SDA results for all subjects. All measures except for the multiclass model scores are averaged by states.*

| **Subject** | **Average IV** | **% of features with IV>0.4** | **% of significant features (adjacent states)** | **% of significant features  (vs median)** | **Multiclass Model Accuracy** | **Multiclass Model  F1 score** | **Binary Balanced Accuracy** | **Binary Classifier ROC AUC** |
| --- | --- | --- | --- | --- | --- | --- | --- | --- |
| **Subj1** | 0.53 | 42% | 32% | 27% | 0.82 | 0.79 | 0.79 | 0.92 |
| **Subj2** | 0.28 | 19% | 23% | 32% | 0.61 | 0.57 | 0.66 | 0.86 |
| **Subj3** | 0.48 | 37% | 19% | 26% | 0.69 | 0.65 | 0.76 | 0.94 |
| **Subj4** | 0.24 | 17% | 19% | 24% | 0.56 | 0.53 | 0.70 | 0.89 |
| **Subj5** | 0.64 | 42% | 44% | 44% | 0.69 | 0.65 | 0.78 | 0.96 |
| **Subj6** | 0.45 | 35% | 35% | 59% | 0.74 | 0.74 | 0.85 | 0.97 |
| **Subj7** | 0.36 | 24% | 20% | 32% | 0.61 | 0.57 | 0.63 | 0.83 |
| **Subj8** | 0.37 | 31% | 40% | 51% | 0.49 | 0.53 | 0.61 | 0.82 |
| **Subj9** | 0.22 | 15% | 28% | 40% | 0.49 | 0.48 | 0.62 | 0.86 |
| **Subj10** | 0.35 | 24% | 16% | 20% | 0.53 | 0.53 | 0.67 | 0.86 |
| **Subj11** | 0.49 | 29% | 36% | 39% | 0.88 | 0.86 | 0.91 | 0.98 |
| **Subj12** | 0.53 | 38% | 54% | 70% | 0.70 | 0.69 | 0.80 | 0.96 |
| **Subj13** | 0.39 | 27% | 42% | 44% | 0.69 | 0.64 | 0.78 | 0.94 |
| **Subj14** | 0.51 | 38% | 31% | 38% | 0.61 | 0.51 | 0.65 | 0.89 |
| **Subj15** | 0.44 | 29% | 40% | 37% | 0.84 | 0.79 | 0.86 | 0.97 |
| **Subj16** | 0.38 | 20% | 33% | 30% | 0.55 | 0.53 | 0.64 | 0.86 |
| **Subj17** | 0.29 | 19% | 38% | 38% | 0.69 | 0.70 | 0.75 | 0.94 |
| **Subj18** | 0.32 | 26% | 42% | 44% | 0.49 | 0.47 | 0.60 | 0.84 |
| **Subj19** | 0.63 | 43% | 45% | 59% | 0.75 | 0.76 | 0.88 | 0.97 |
| **Subj20** | 0.36 | 25% | 16% | 24% | 0.58 | 0.48 | 0.65 | 0.87 |
| **Subj21** | 0.48 | 38% | 44% | 47% | 0.70 | 0.67 | 0.78 | 0.95 |
| **Subj22** | 0.41 | 26% | 38% | 36% | 0.66 | 0.63 | 0.70 | 0.88 |
| **Subj23** | 0.40 | 27% | 47% | 48% | 0.69 | 0.69 | 0.81 | 0.95 |
| **Subj24** | 0.83 | 44% | 52% | 66% | 0.83 | 0.77 | 0.84 | 0.96 |
| **Subj25** | 0.21 | 14% | 18% | 21% | 0.49 | 0.41 | 0.61 | 0.79 |
| **Subj26** | 0.47 | 35% | 49% | 47% | 0.59 | 0.55 | 0.64 | 0.85 |
| **Subj27** | 0.64 | 41% | 50% | 52% | 0.83 | 0.83 | 0.89 | 0.99 |
| **Subj28** | 0.79 | 55% | 47% | 59% | 0.82 | 0.78 | 0.87 | 0.98 |
| **Subj29** | 0.27 | 16% | 42% | 30% | 0.76 | 0.76 | 0.78 | 0.89 |
| **Subj30** | 0.38 | 27% | 37% | 31% | 0.77 | 0.70 | 0.80 | 0.93 |

| ***Supplementary Table 4.*** *Boundaries and lengths of states obtained by SDA for practitioners and surrogate data.* |
| --- |
| 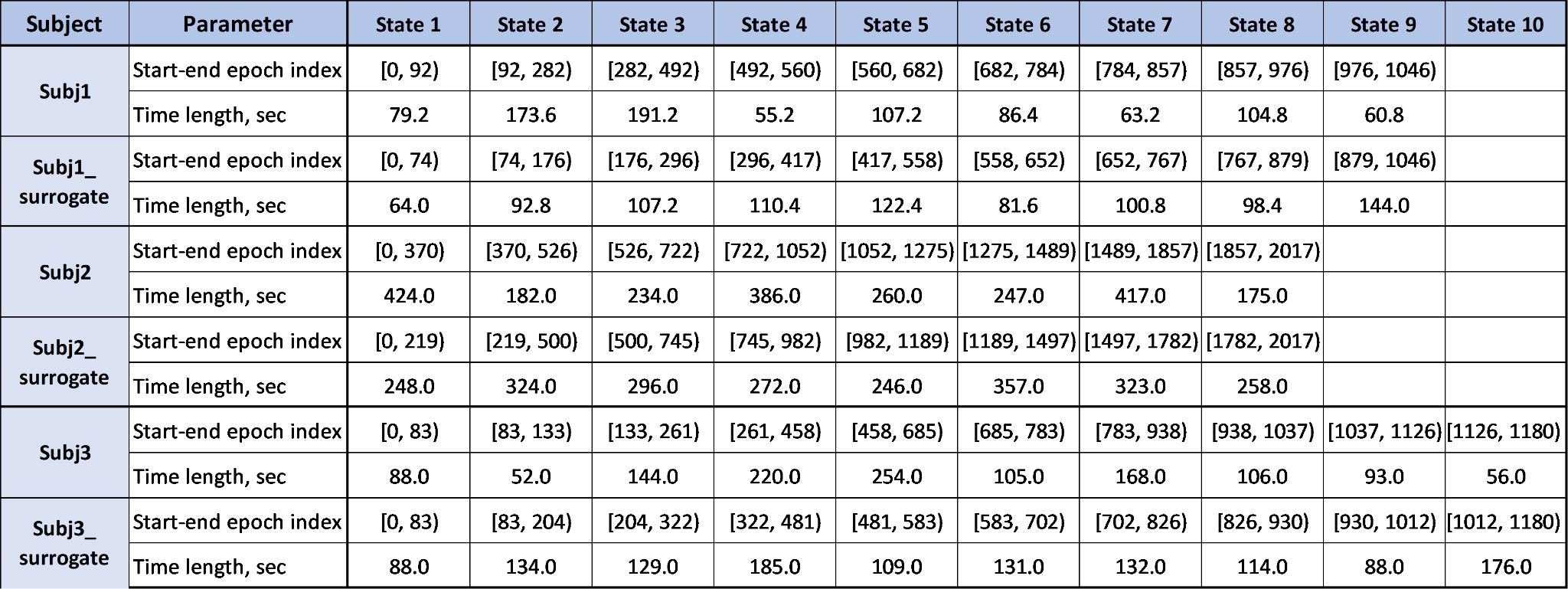 |

| ***Supplementary Table 5.*** *Clustering quality measures on pairs of adjacent states obtained by SDA* |  |
| --- | --- |
| 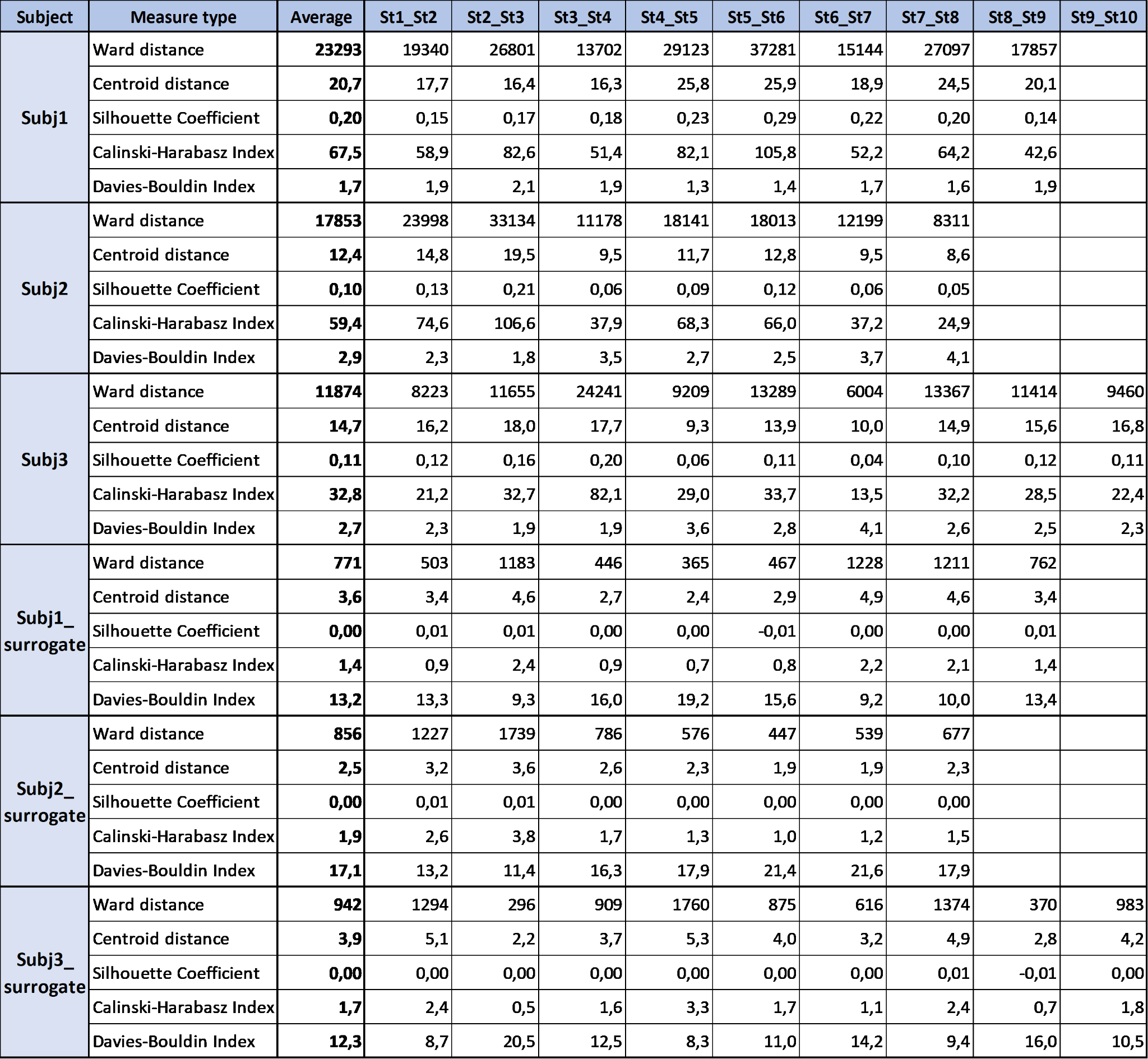 |  |

| ***Supplementary Table 6.*** *Boundaries and lengths of states obtained by SDA and of rearranged initial states for practitioners’ EEG data with shuffled states.* |
| --- |
| 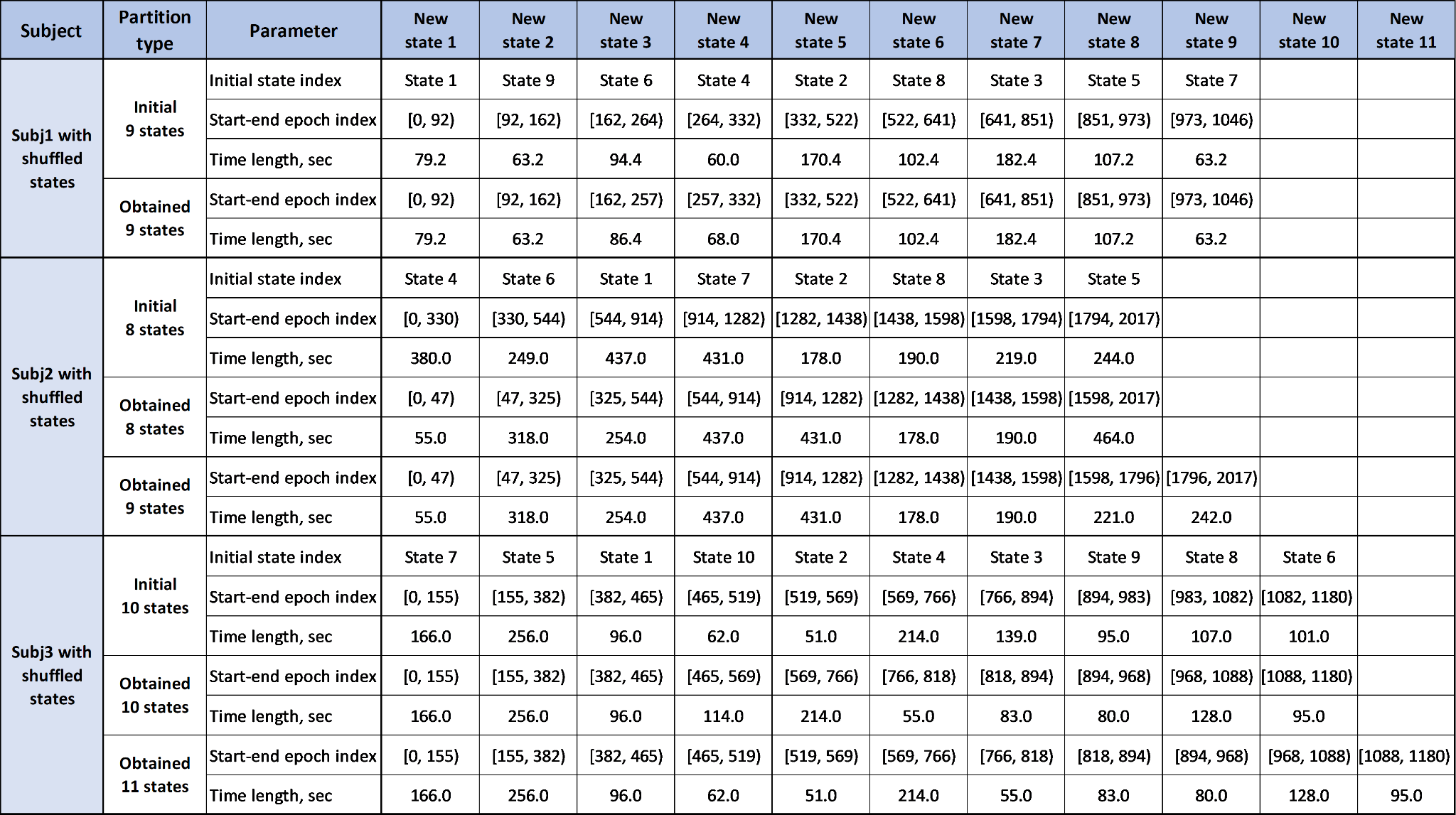 |

| ***Supplementary Table 7.*** *Clustering quality measures on pairs of adjacent states, obtained by SDA, and on rearranged initial states for practitioners’ EEG data with shuffled states.* |
| --- |
| 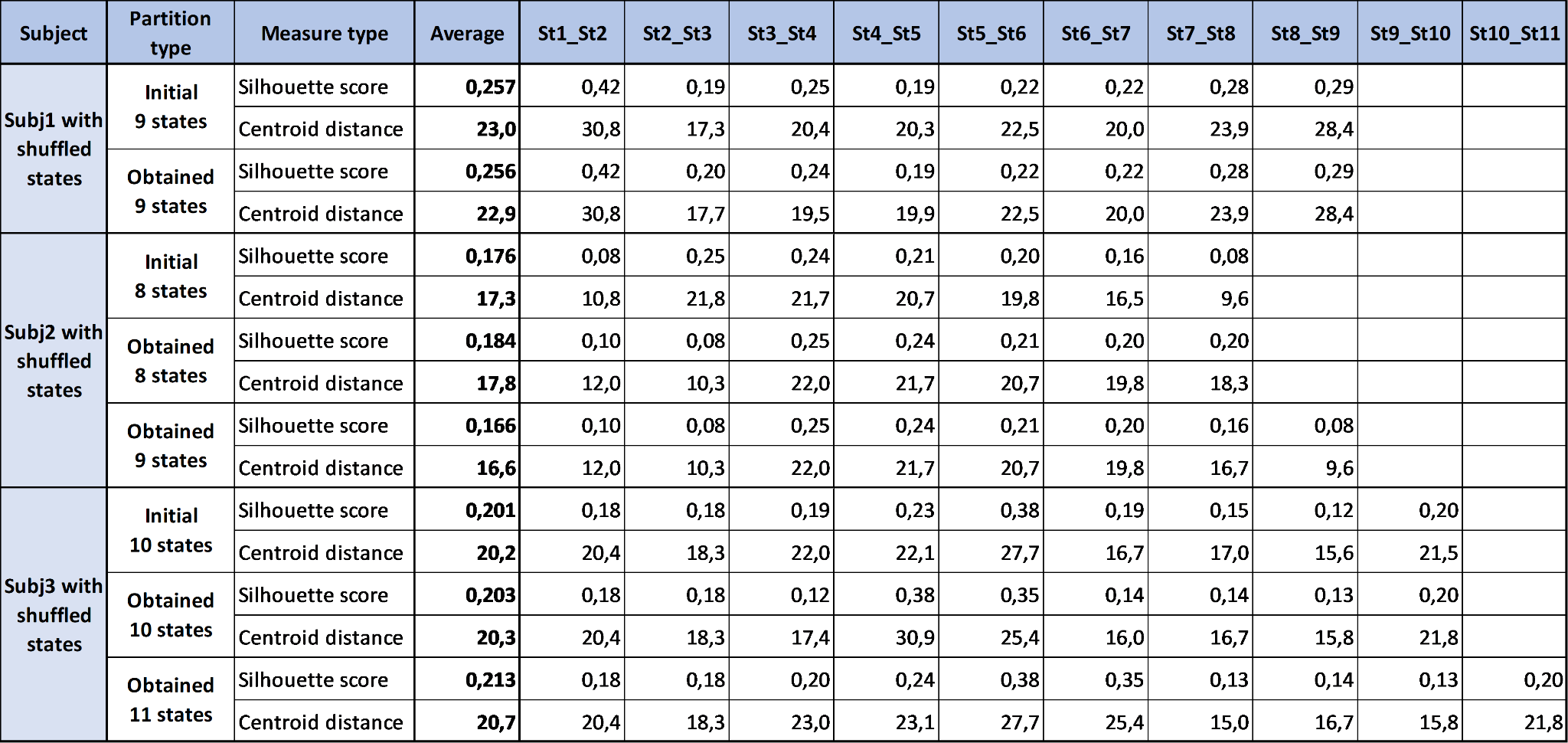 |

| ***Supplementary Table 8.*** *SDA resulting hyperparameters for practitioners and surrogate data.* |
| --- |
| 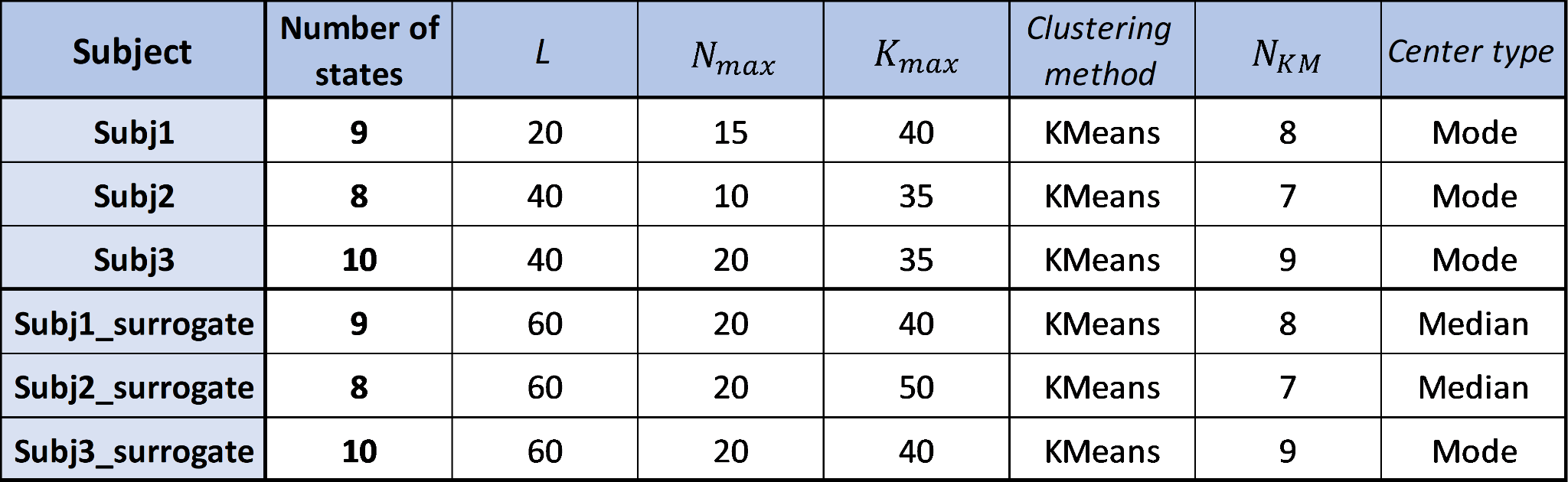 |

| 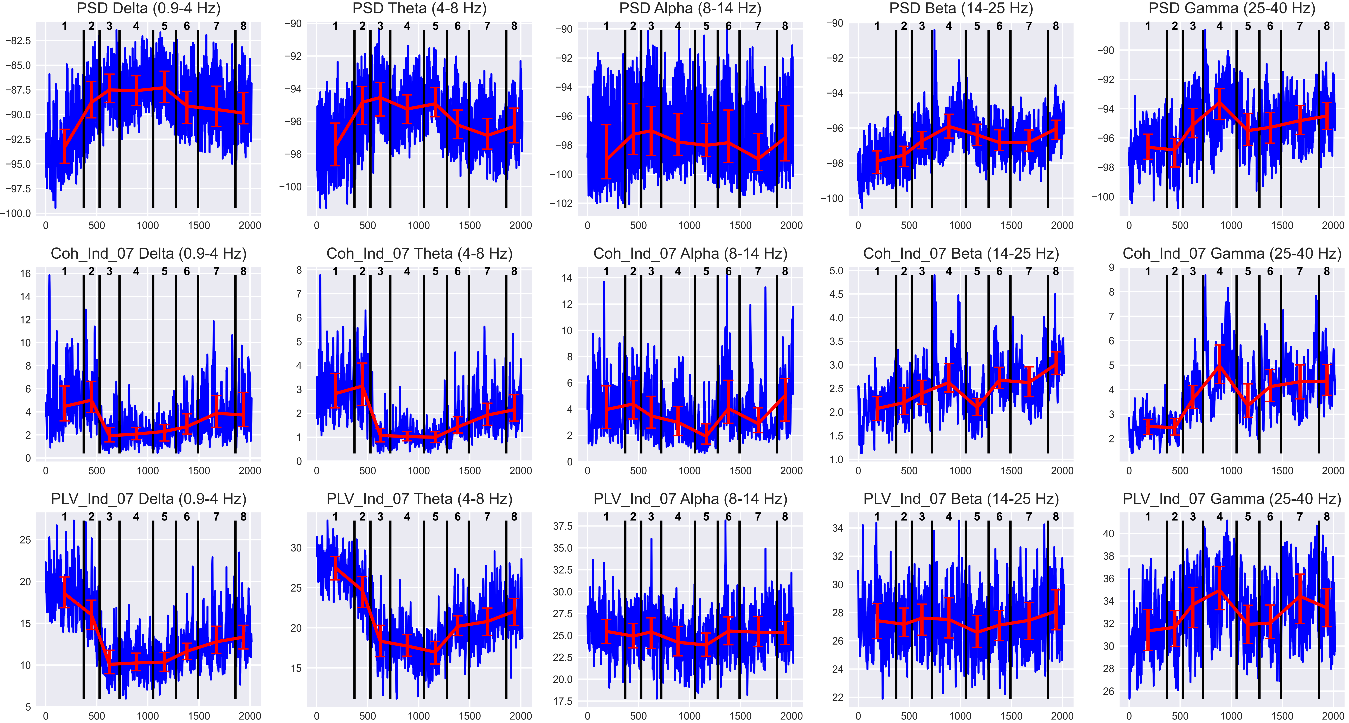 |
| --- |
| **Supplementary Figure 1.** EEG features behavior in states of practitioner Subj2 EEG data, obtained by SDA. Refer to Figure 6 caption. |

| 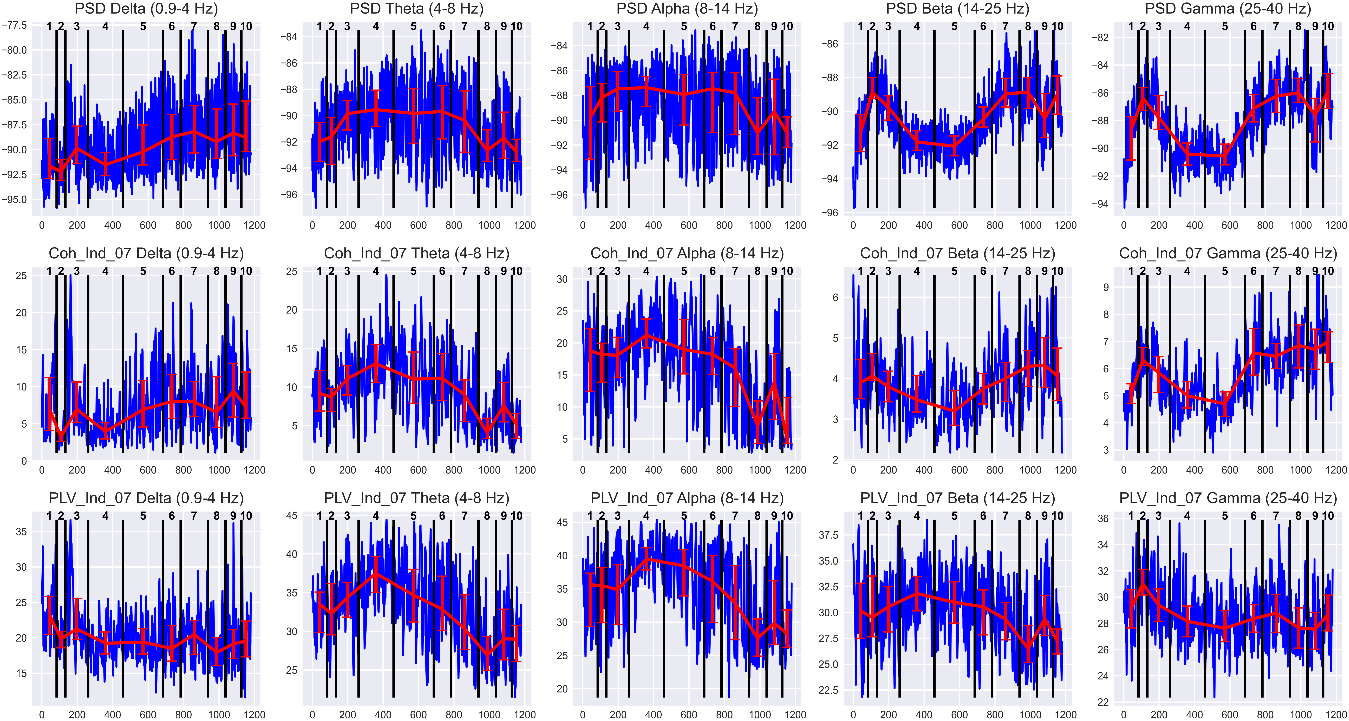 |
| --- |
| **Supplementary Figure 2.** EEG features behavior in states of practitioner Subj3 EEG data, obtained by SDA. Refer to Figure 6 caption. |

| 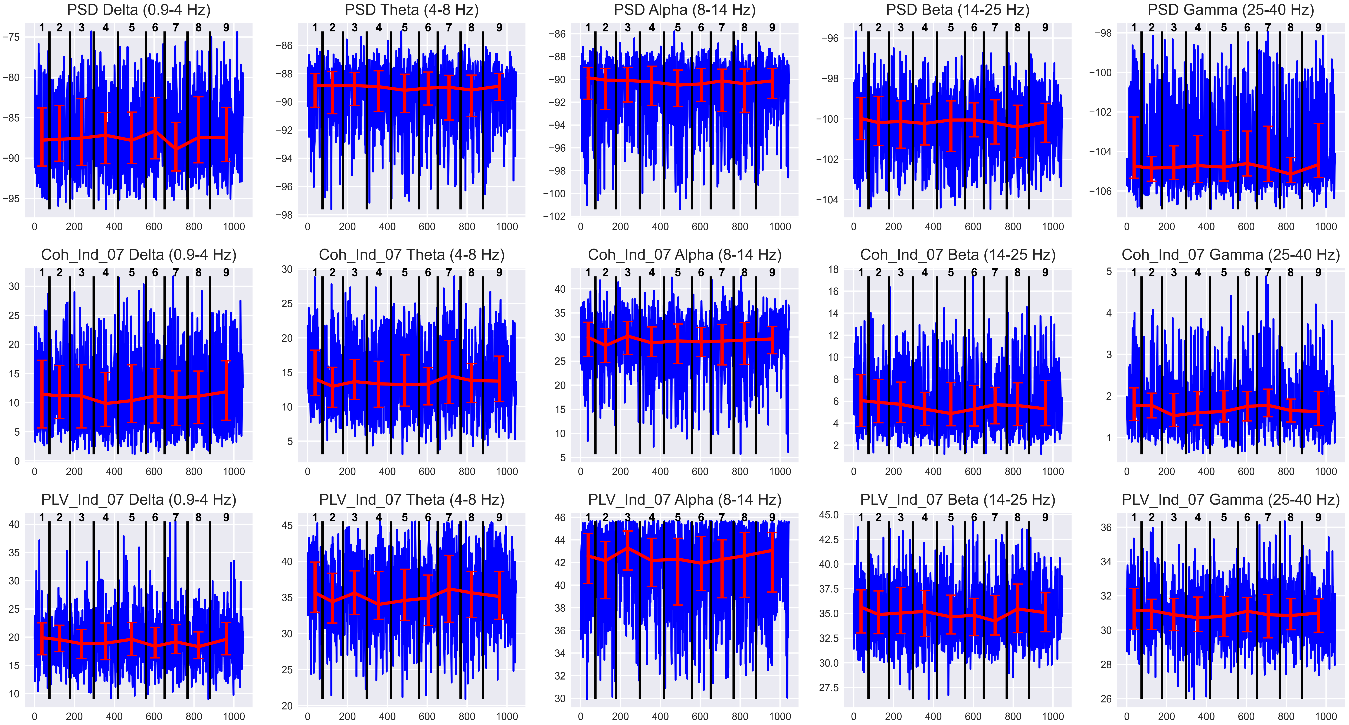 |
| --- |
| **Supplementary Figure 3.** EEG features behavior in states of surrogate data Subj1_surrogate, obtained by SDA. Refer to Figure 6 caption. |

| 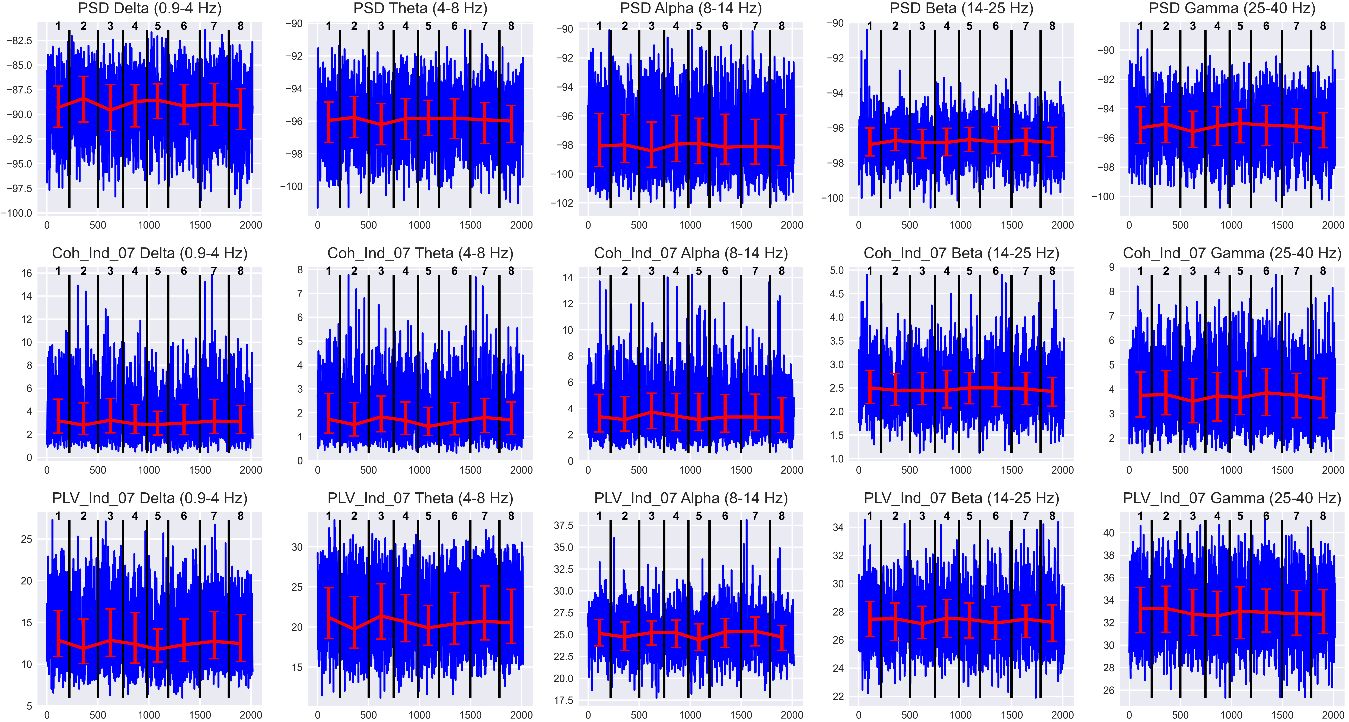 |
| --- |
| **Supplementary Figure 4.** EEG features behavior in states of surrogate data Subj2_surrogate, obtained by SDA. Refer to Figure 6 caption. |

| 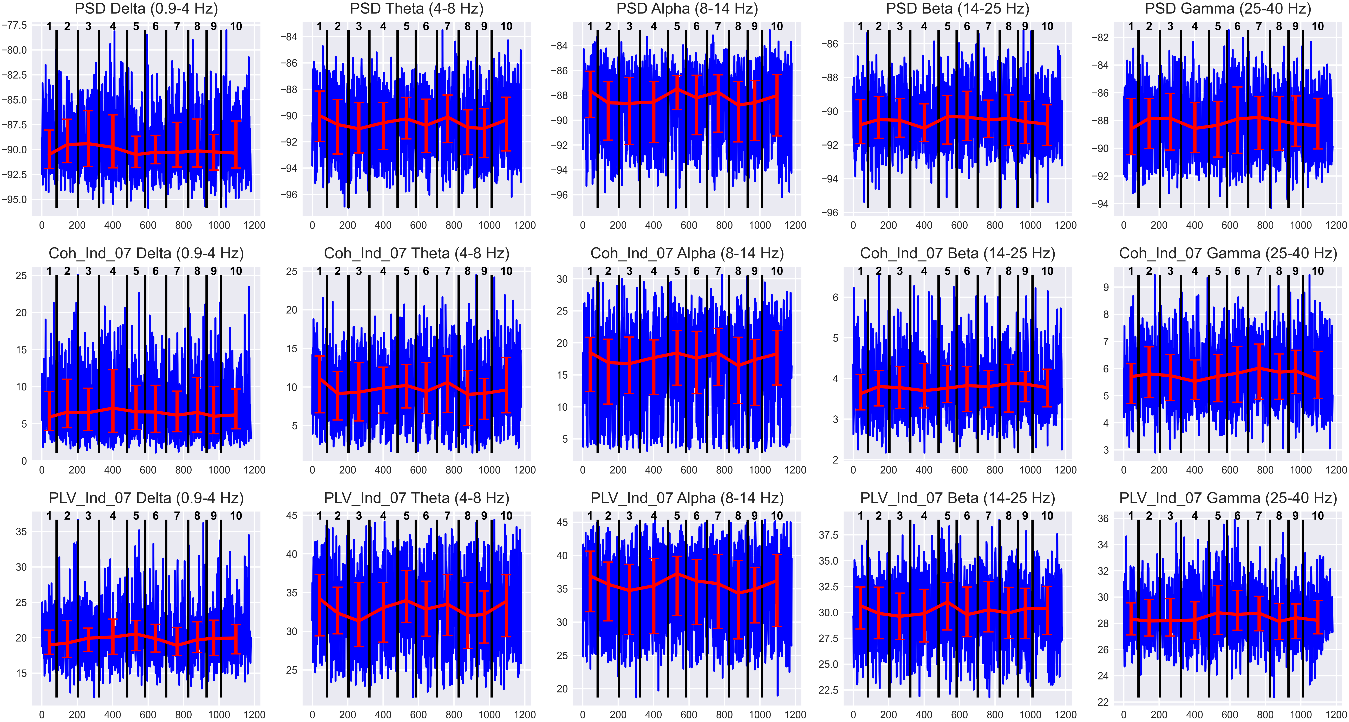 |
| --- |
| **Supplementary Figure 5.** EEG features behavior in states of surrogate data Subj3_surrogate, obtained by SDA. Refer to Figure 6 caption. |

| 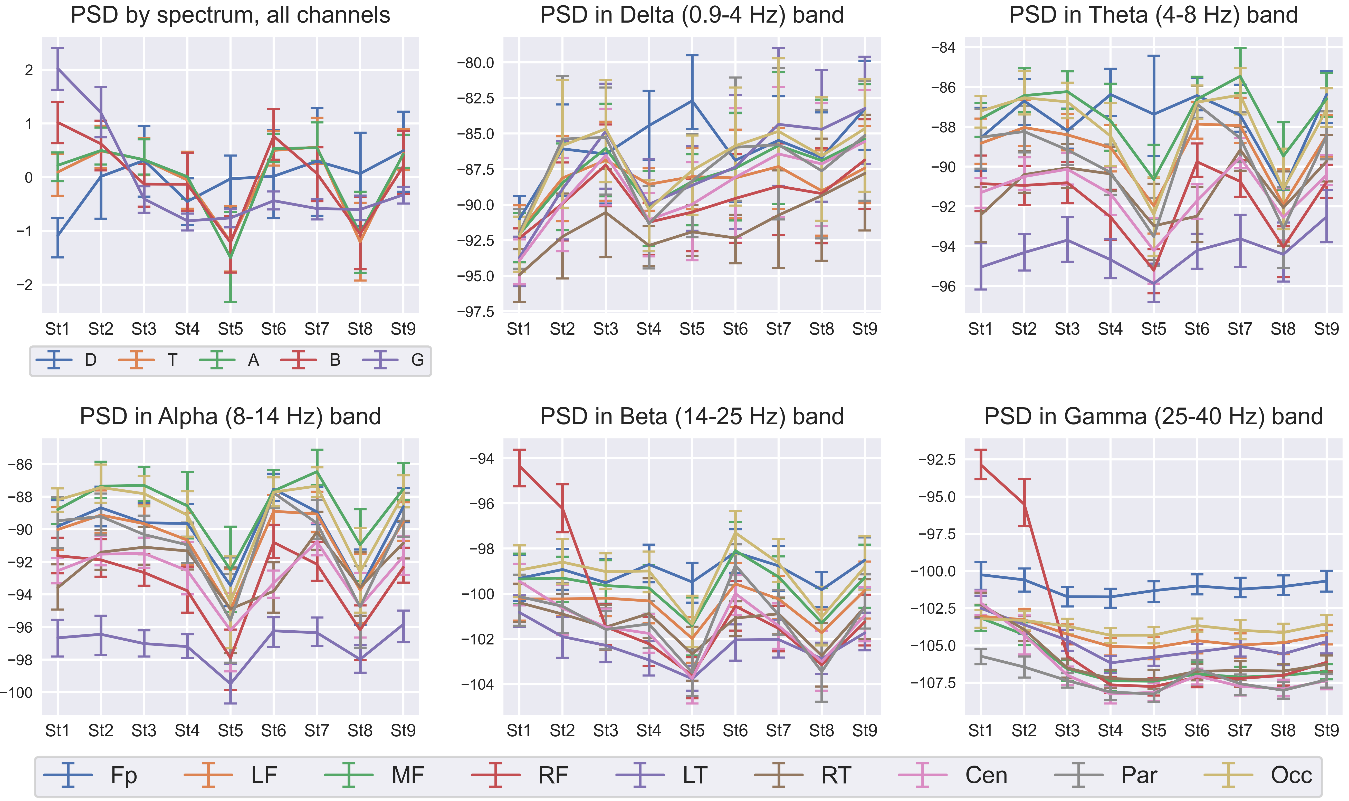 |
| --- |
| **Supplementary Figure 6.** PSD interstate dynamics in 5 frequency bands and 9 spatial ROIs in Subj1’s meditation. Refer to Figure 7 caption. |

| 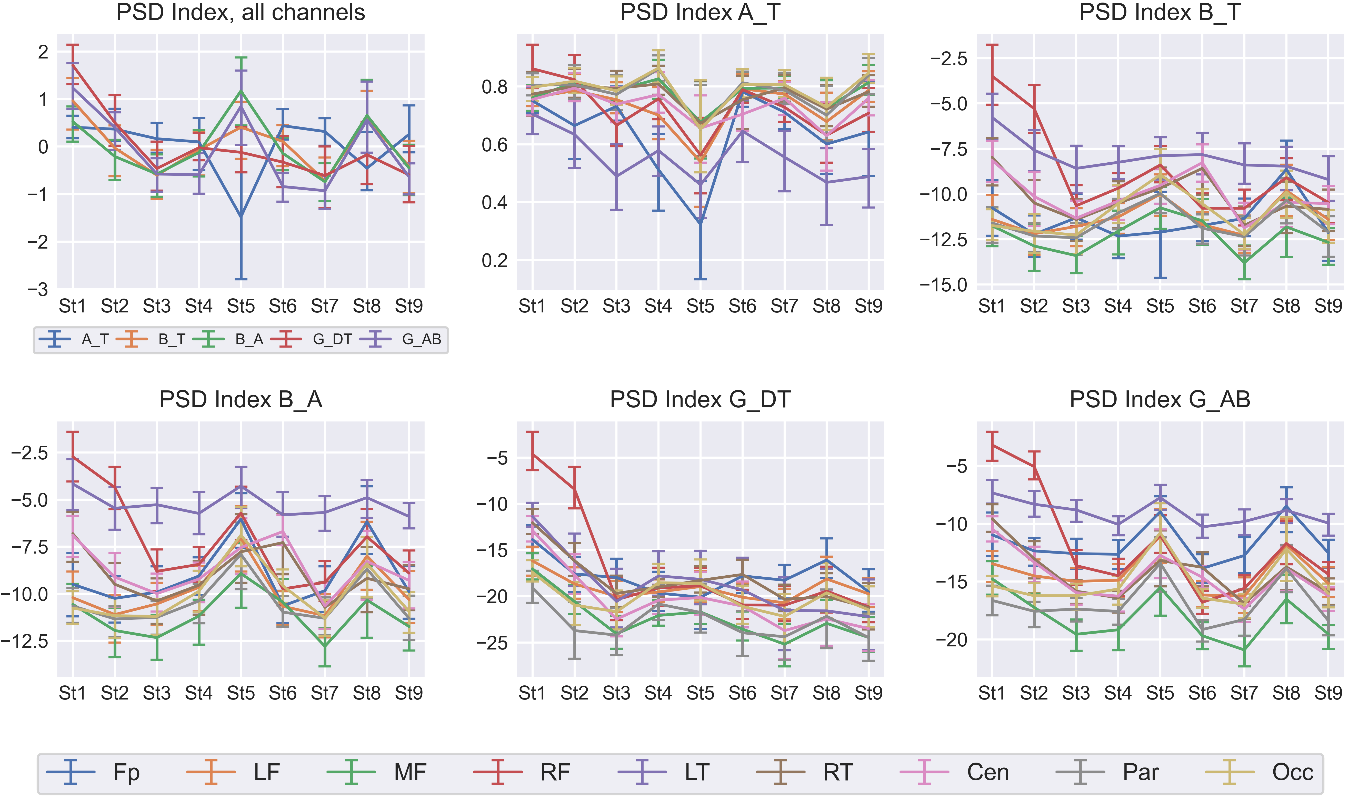 |
| --- |
| **Supplementary Figure 7.** PSD Ratios interstate dynamics in 9 spatial ROIs in Subj1 meditation. Box in the left upper corner illustrates z-scored 5 PSD Indices averaged over all 38 channels – Alpha/Theta (A_T – blue line), Beta/Theta (B_T – orange line), Beta/Alpha (B_A – green line), Gamma/(Delta+Theta) (G_DT – red line) and Gamma/(Alpha+Beta) (G_AB – purple line). The remaining 5 boxes illustrate the average dynamics of these 5 PSD Ratios in 9 spatial ROIs. Refer to Figure 7 caption. |

| 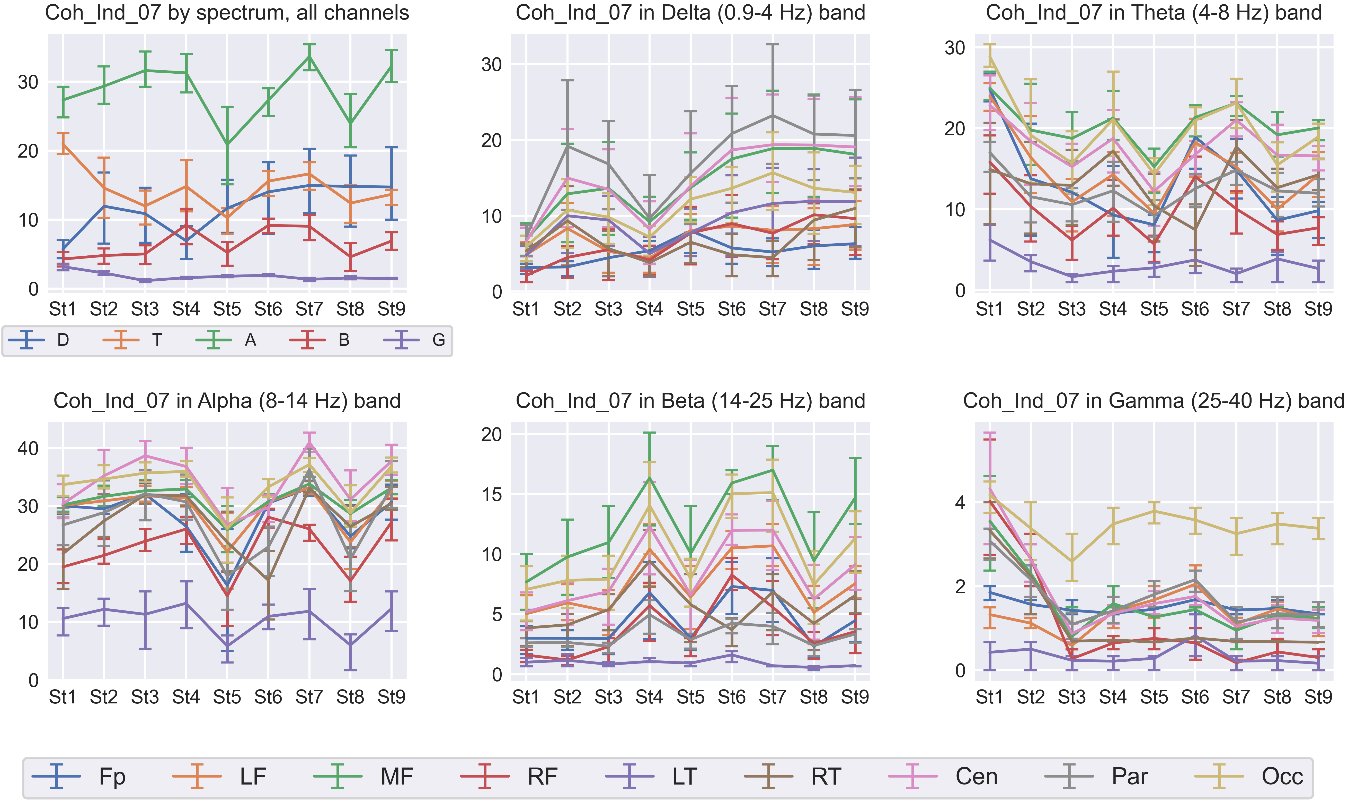 |
| --- |
| **Supplementary Figure 8.** Interstate dynamics of Coherence Index with threshold 0.7 in 5 frequency bands and 9 spatial ROIs in Subj1 meditation. Box in the left upper corner illustrates Coherence Indices averaged over all 38 channels for 5 frequency bands. With respect to 5 frequency bands the rest 5 boxes illustrate the average dynamics of Coherence Indices in 9 spatial ROIs. Refer to Figure 7 caption. |

| 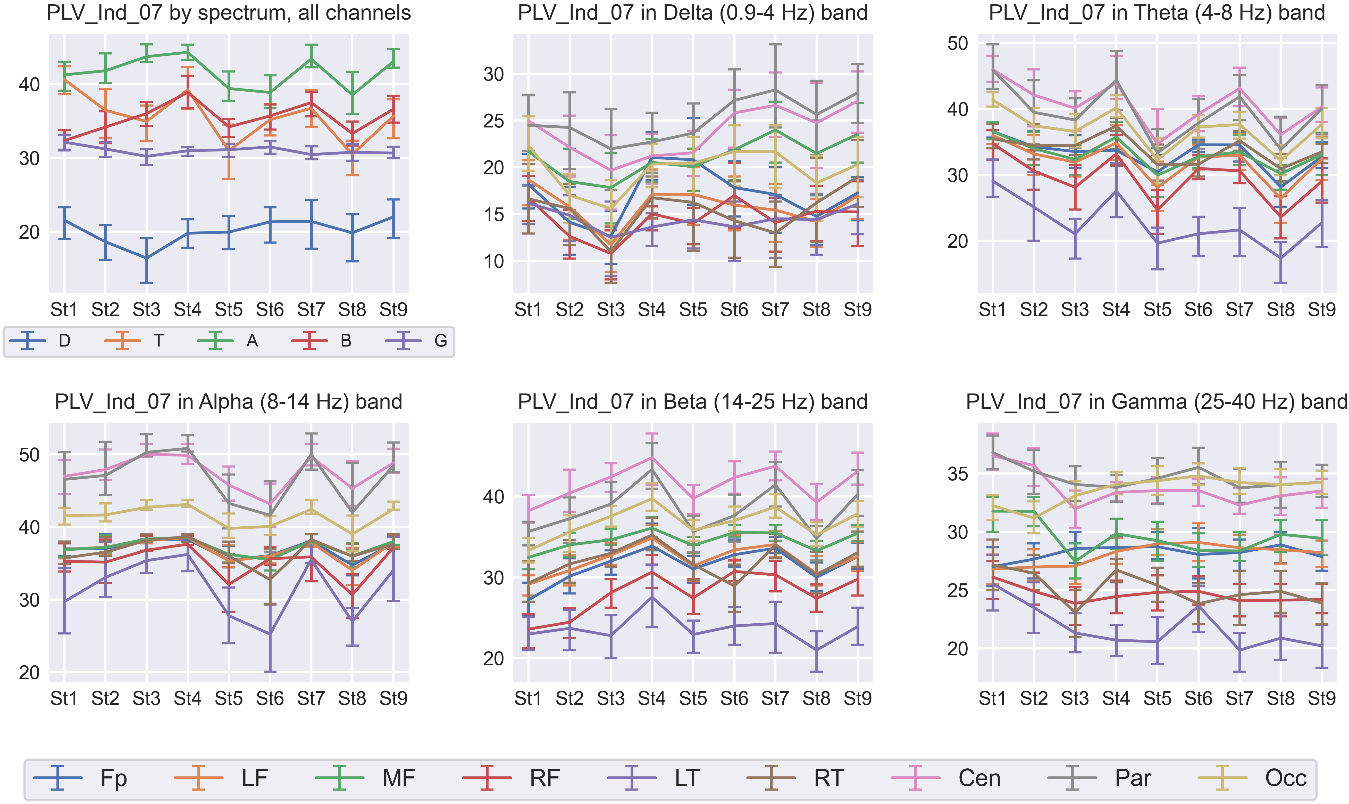 |
| --- |
| **Supplementary Figure 9.** Interstate dynamics of PLV Index with threshold 0.7 in 5 frequency bands and 9 spatial ROIs in Subj1’s meditation. Box in the left upper corner illustrates PLV Indices averaged over all 38 channels for 5 frequency bands. With respect to 5 frequency bands the rest 5 boxes illustrate the average dynamics of PLV Indices in 9 spatial ROIs. Refer to Figure 7 caption. |

| 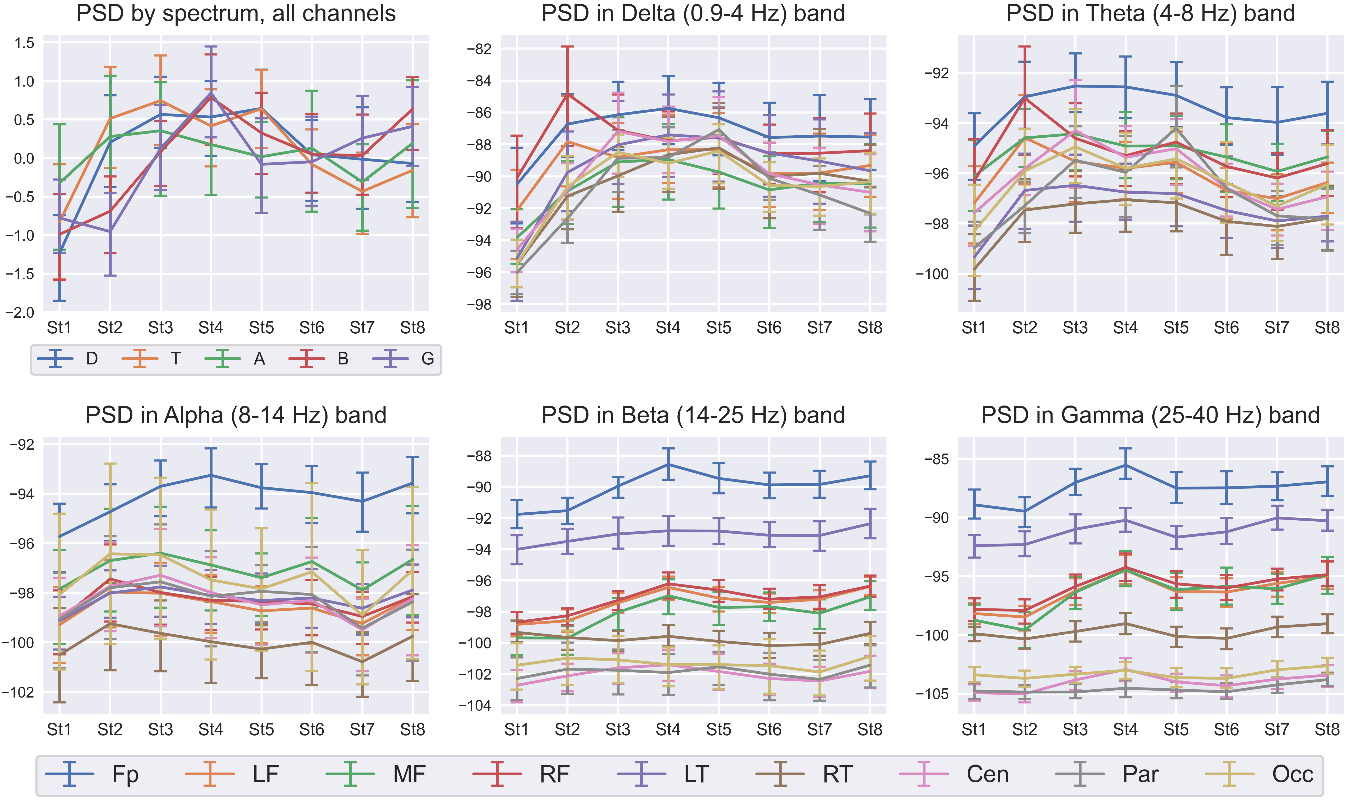 |
| --- |
| **Supplementary Figure 10.** PSD interstate dynamics in 5 frequency bands and 9 spatial ROIs in Subj2’s meditation. Refer to Figure 7 caption. |

| 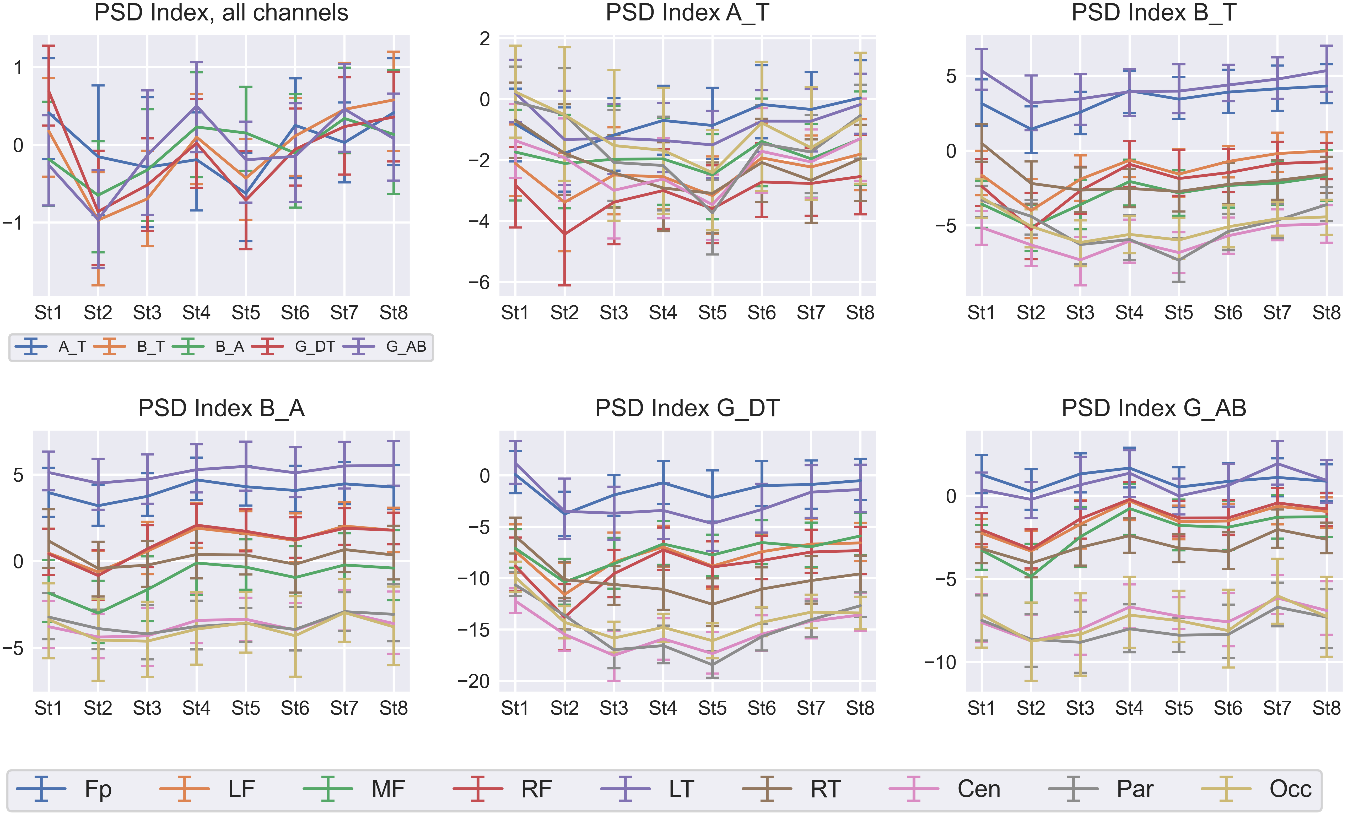 |
| --- |
| **Supplementary Figure 11.** PSD Ratios interstate dynamics in 9 spatial ROIs in Subj2’s meditation. Box in the left upper corner illustrates z-scored 5 PSD Indices averaged over all 38 channels – Alpha/Theta (A_T – blue line), Beta/Theta (B_T – orange line), Beta/Alpha (B_A – green line), Gamma/(Delta+Theta) (G_DT – red line) and Gamma/(Alpha+Beta) (G_AB – purple line). The remaining 5 boxes illustrate the average dynamics of these 5 PSD Ratios in 9 spatial ROIs. Refer to Figure 7 caption. |
| 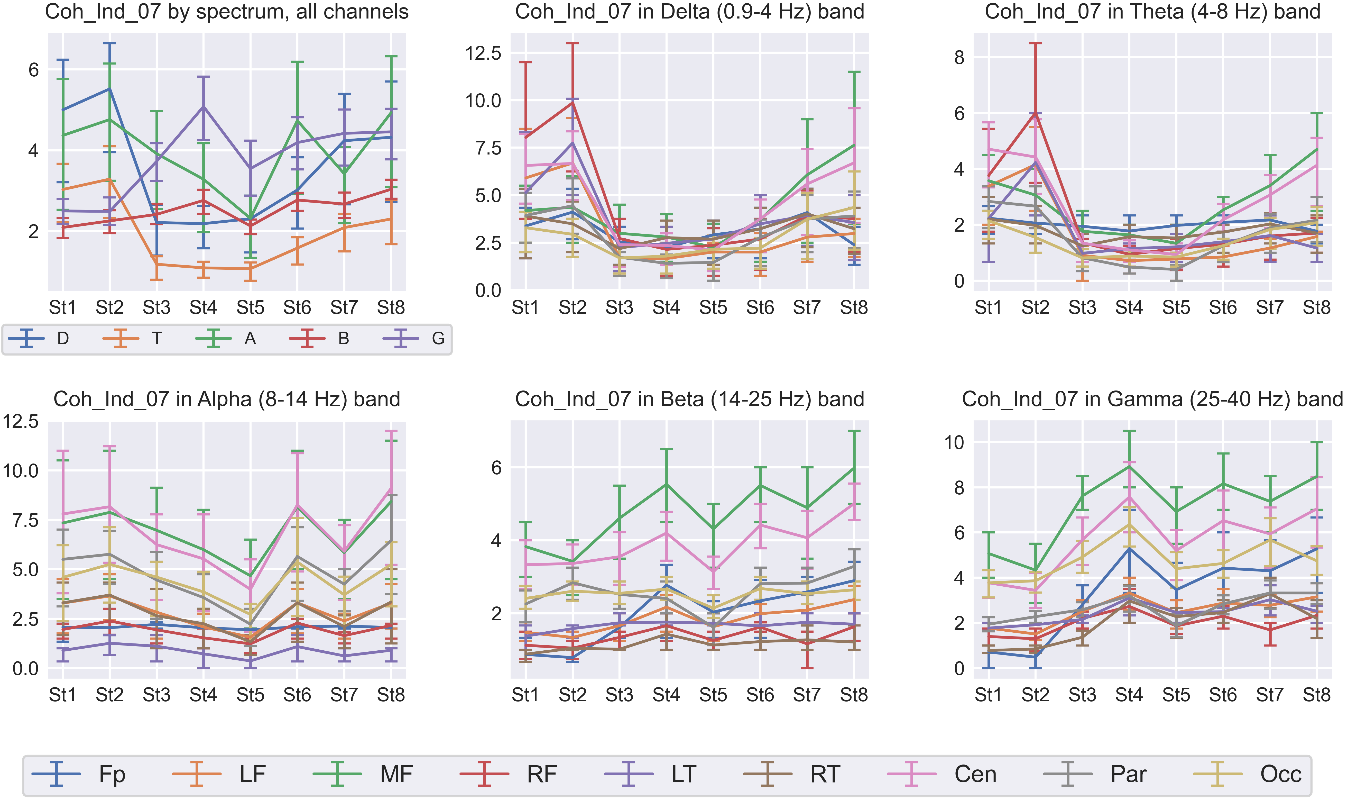 |
| **Supplementary Figure 12.** Interstate dynamics of Coherence Index with threshold 0.7 in 5 frequency bands and 9 spatial ROIs in Subj2’s meditation. Box in the left upper corner illustrates Coherence Indices averaged over all 38 channels for 5 frequency bands. With respect to 5 frequency bands the rest 5 boxes illustrate the average dynamics of Coherence Indices in 9 spatial ROIs. Refer to Figure 7 caption. |

| 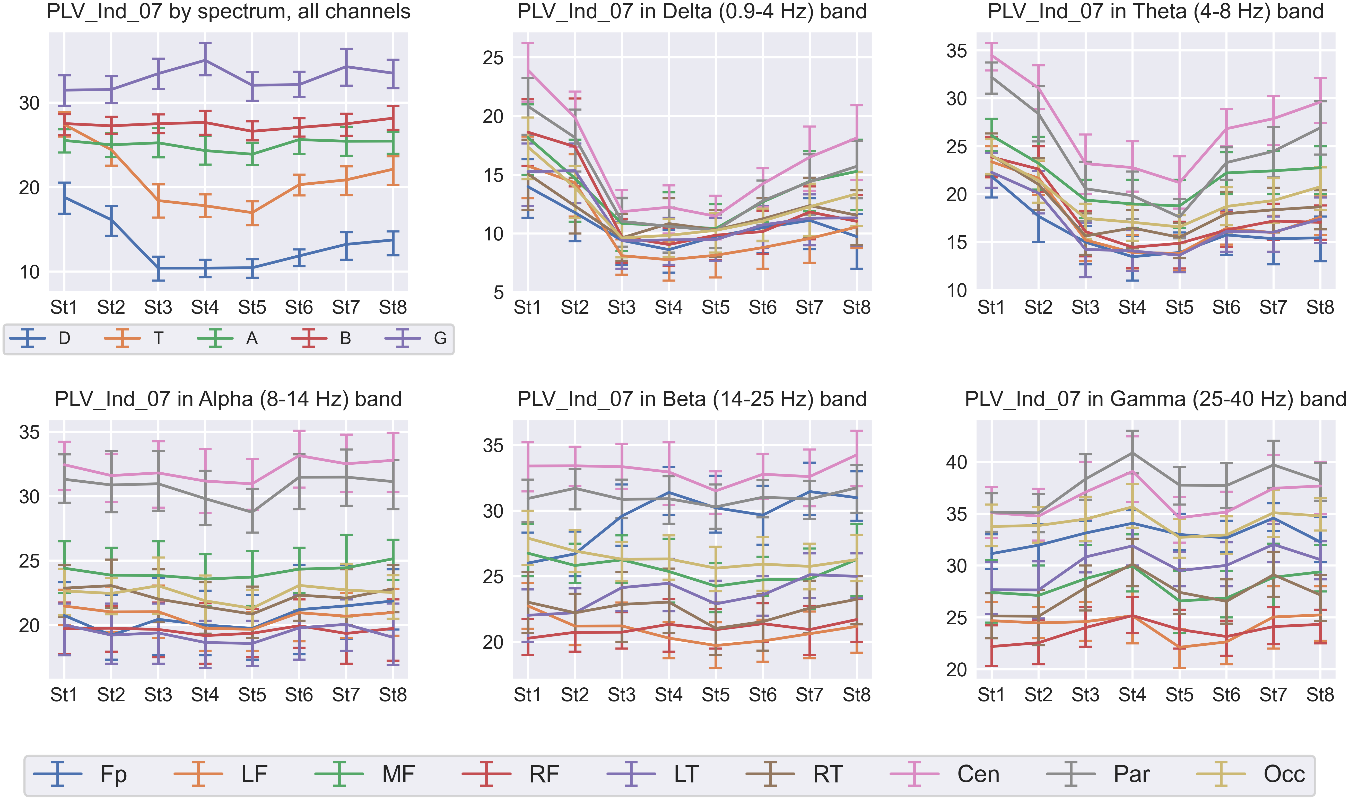 |
| --- |
| **Supplementary Figure 13.** Interstate dynamics of PLV Index with threshold 0.7 in 5 frequency bands and 9 spatial ROIs in Subj2’s meditation. Box in the left upper corner illustrates PLV Indices averaged over all 38 channels for 5 frequency bands. With respect to 5 frequency bands the rest 5 boxes illustrate the average dynamics of PLV Indices in 9 spatial ROIs. Refer to Figure 7 caption. |

| 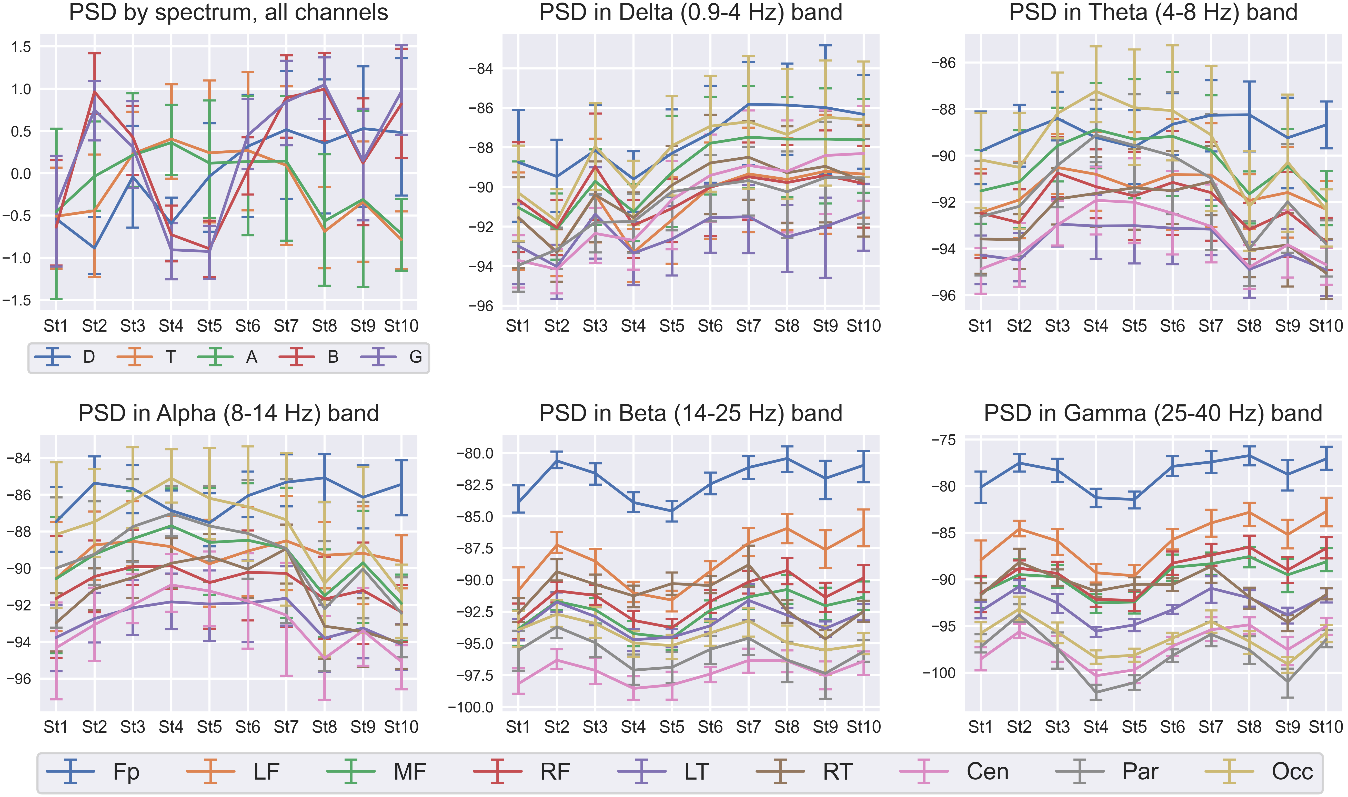 |
| --- |
| **Supplementary Figure 14.** PSD interstate dynamics in 5 frequency bands and 9 spatial ROIs in Subj3’s meditation. Refer to Figure 7 caption. |

| 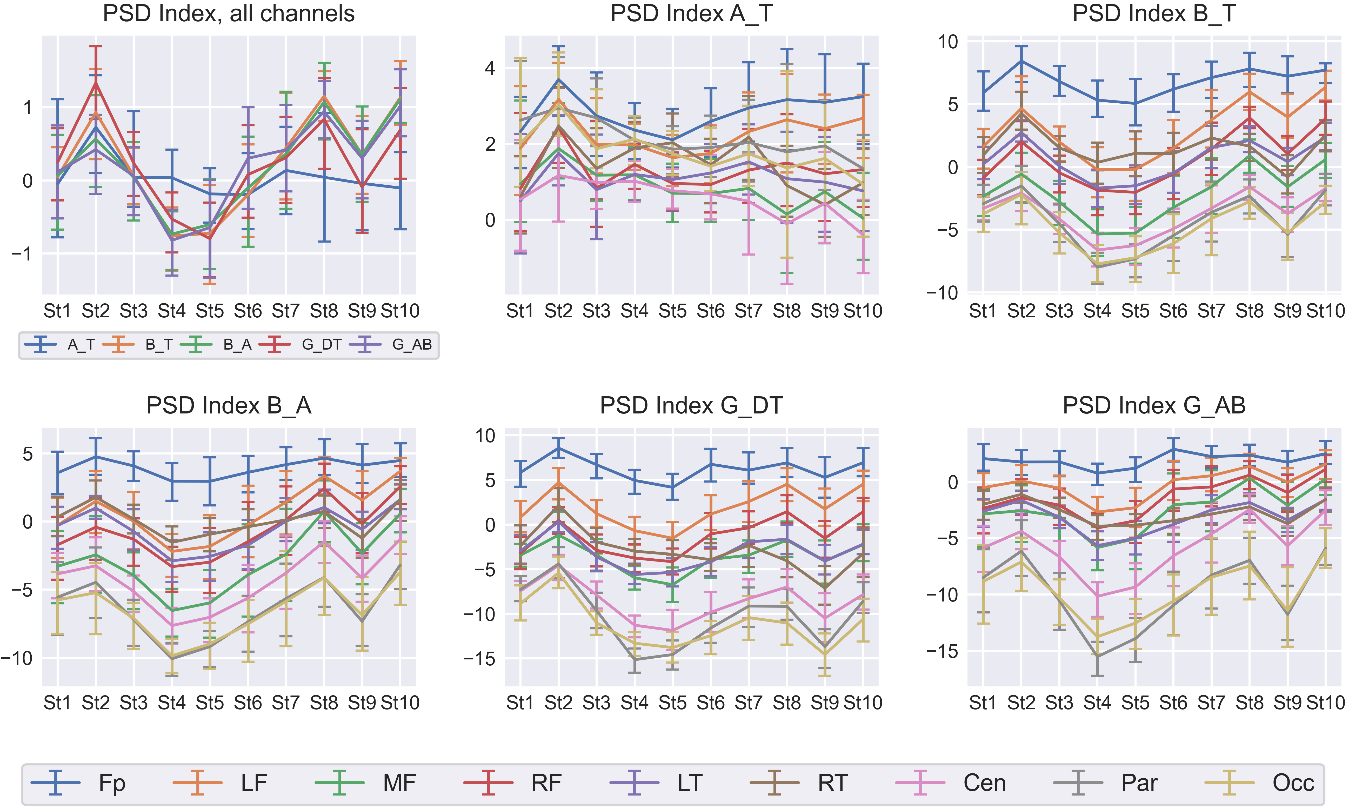 |
| --- |
| **Supplementary Figure 15.** PSD Ratios interstate dynamics in 9 spatial ROIs in Subj3’s meditation. Box in the left upper corner illustrates z-scored 5 PSD Indices averaged over all 38 channels – Alpha/Theta (A_T – blue line), Beta/Theta (B_T – orange line), Beta/Alpha (B_A – green line), Gamma/(Delta+Theta) (G_DT – red line) and Gamma/(Alpha+Beta) (G_AB – purple line). The remaining 5 boxes illustrate the average dynamics of these 5 PSD Ratios in 9 spatial ROIs. Refer to Figure 7 caption. |

| 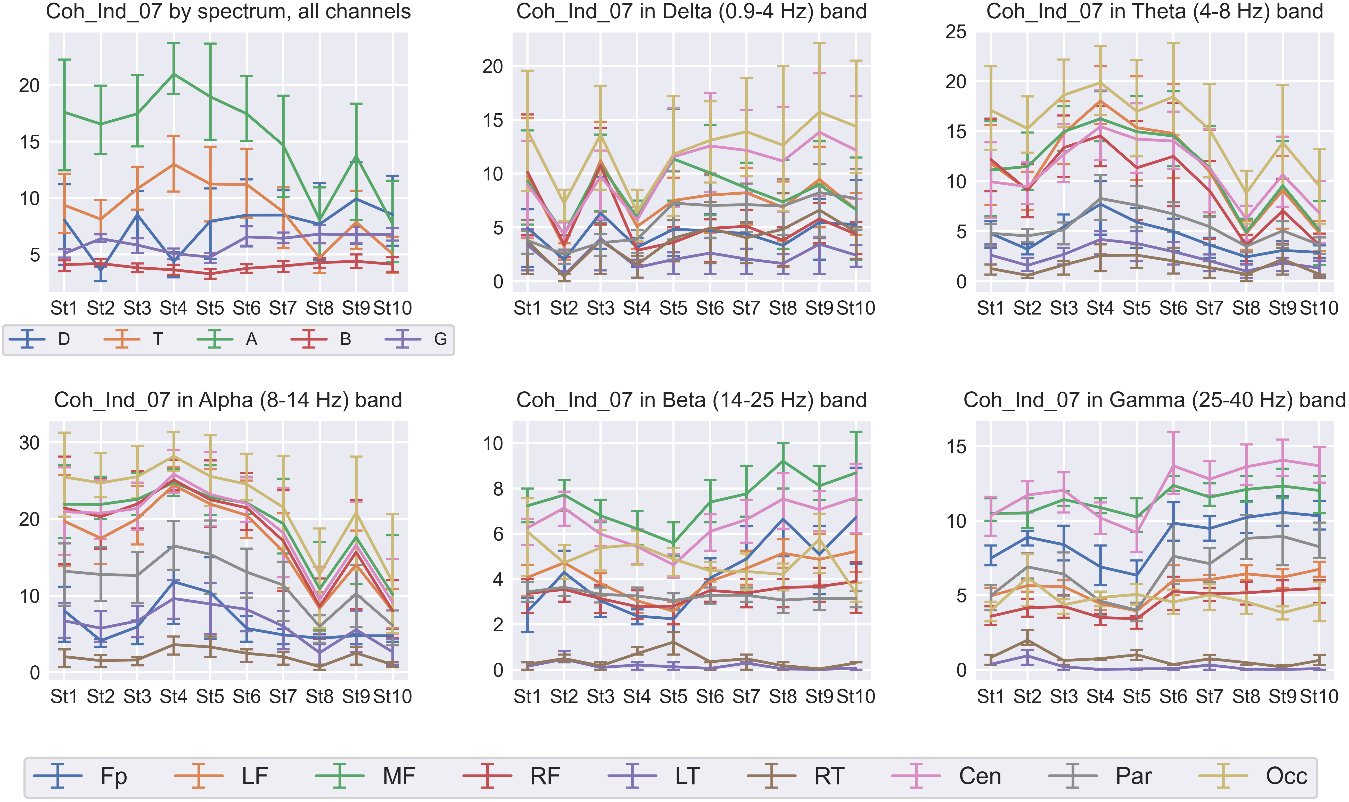 |
| --- |
| **Supplementary Figure 16.** Interstate dynamics of Coherence Index with threshold 0.7 in 5 frequency bands and 9 spatial ROIs in Subj3’s meditation. Box in the left upper corner illustrates Coherence Indices averaged over all 38 channels for 5 frequency bands. With respect to 5 frequency bands the rest 5 boxes illustrate the average dynamics of Coherence Indices in 9 spatial ROIs. Additional description is similar to Figure 7 in the paper. |

| 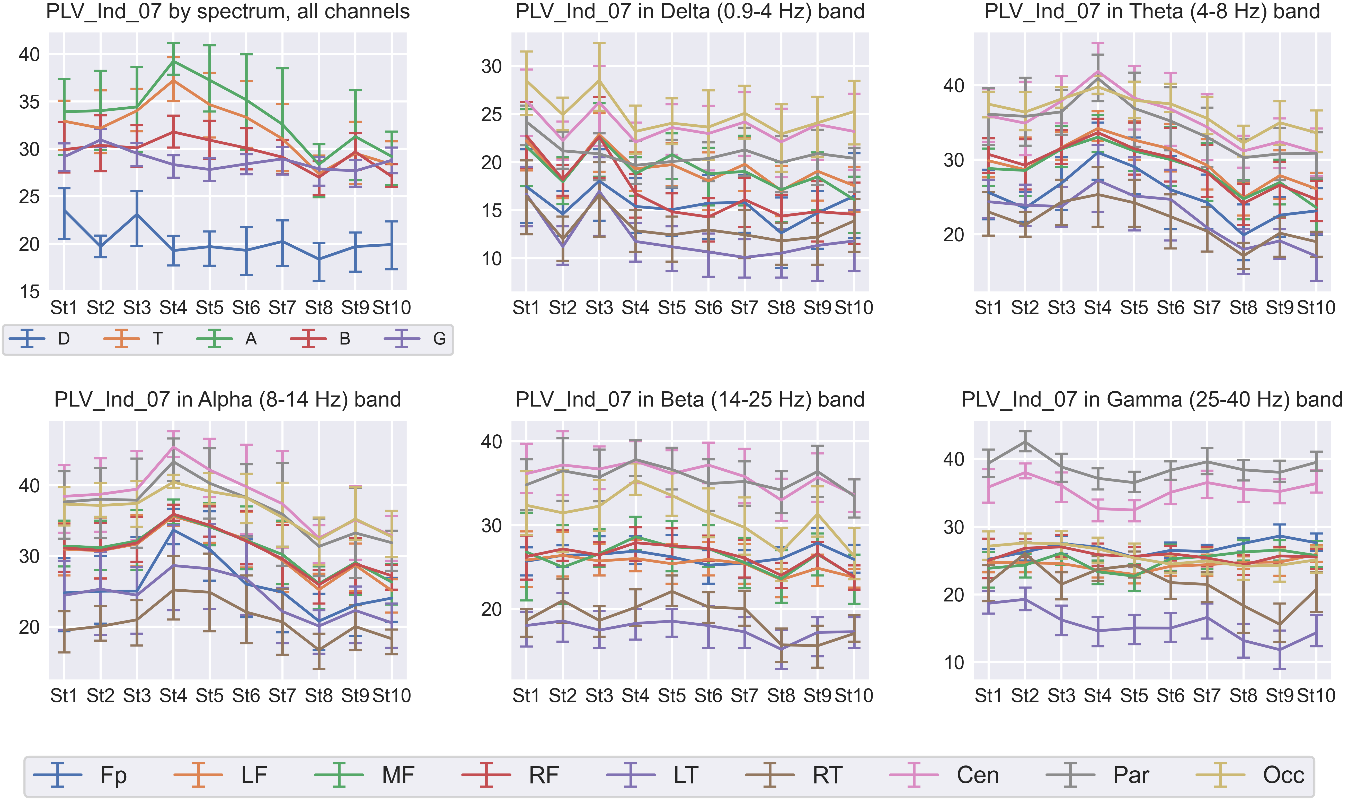 |
| --- |
| **Supplementary Figure 17.** Interstate dynamics of PLV Index with threshold 0.7 in 5 frequency bands and 9 spatial ROIs in Subj3’s meditation. Box in the left upper corner illustrates PLV Indices averaged over all 38 channels for 5 frequency bands. With respect to 5 frequency bands the rest 5 boxes illustrate the average dynamics of PLV. Refer to Figure 7 caption. |

| ***Supplementary Table 9. State pairwise statistical significance a)*** *in practitioner’s Subj1 EEG data;* ***b)*** *in surrogate data Subj1_surrogate. For each pair of states, obtained by SDA, at the intersection of the corresponding row and column the percentage of significantly different features is indicated according to Mann-Whitney U-test with Bonferroni correction (p<0.01).* |
| --- |
| 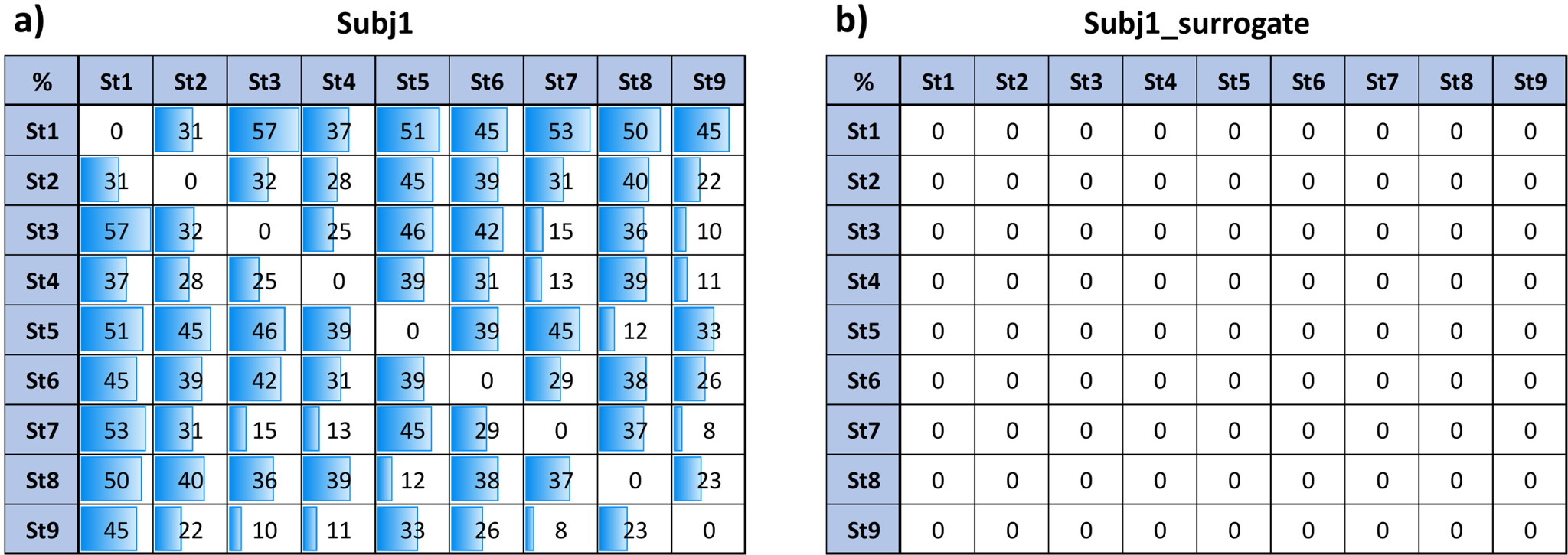 |

| ***Supplementary Table 10. State pairwise statistical significance a)*** *in practitioner’s Subj2 EEG data;* ***b)*** *in surrogate data Subj2_surrogate. For each pair of states a percent of significantly different features is given according to Mann-Whitney U-test with Bonferroni correction (p<0.01).* |
| --- |
| 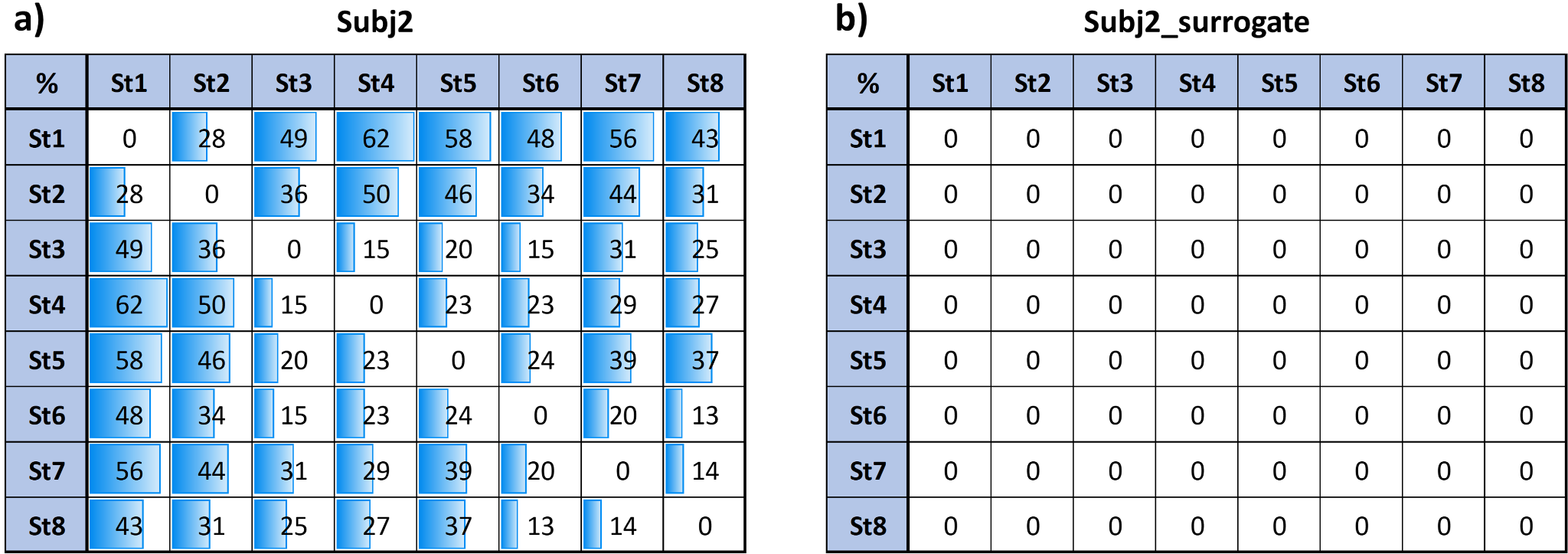 |

| ***Supplementary Table 11. State pairwise statistical significance a)*** *in practitioner’s Subj3 EEG data;* ***b)*** *in surrogate data Subj3_surrogate. For each pair of states a percent of significantly different features is given according to Mann-Whitney U-test with Bonferroni correction (p<0.01).* |
| --- |
| 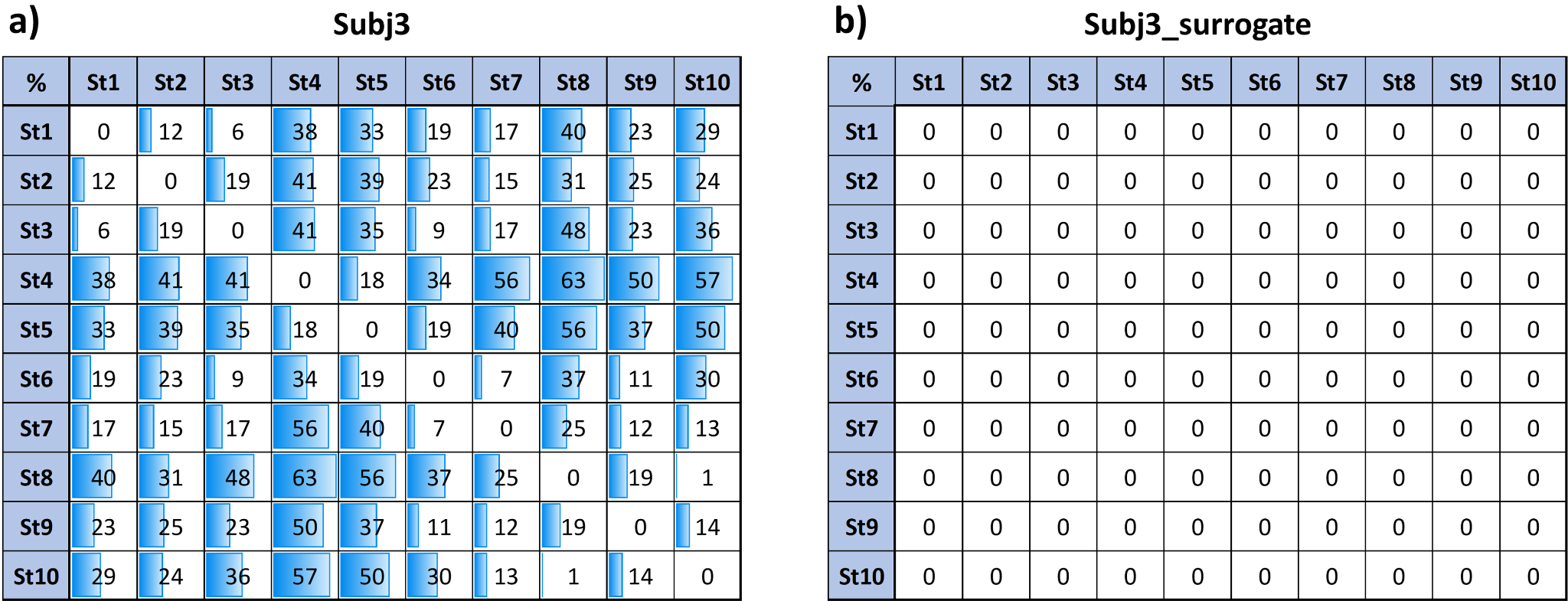 |

| ***Supplementary Table 12.*** *Statistical significance of features in states versus feature general median value for Subj1 in comparison with Subj1_surrogate. Detailed statistical significance in different groups of features for Subj1. For each state a percent of features, significantly different from median value, is given according to one-sample Wilcoxon signed-rank test with Bonferroni correction (p<0.01).* |
| --- |
| 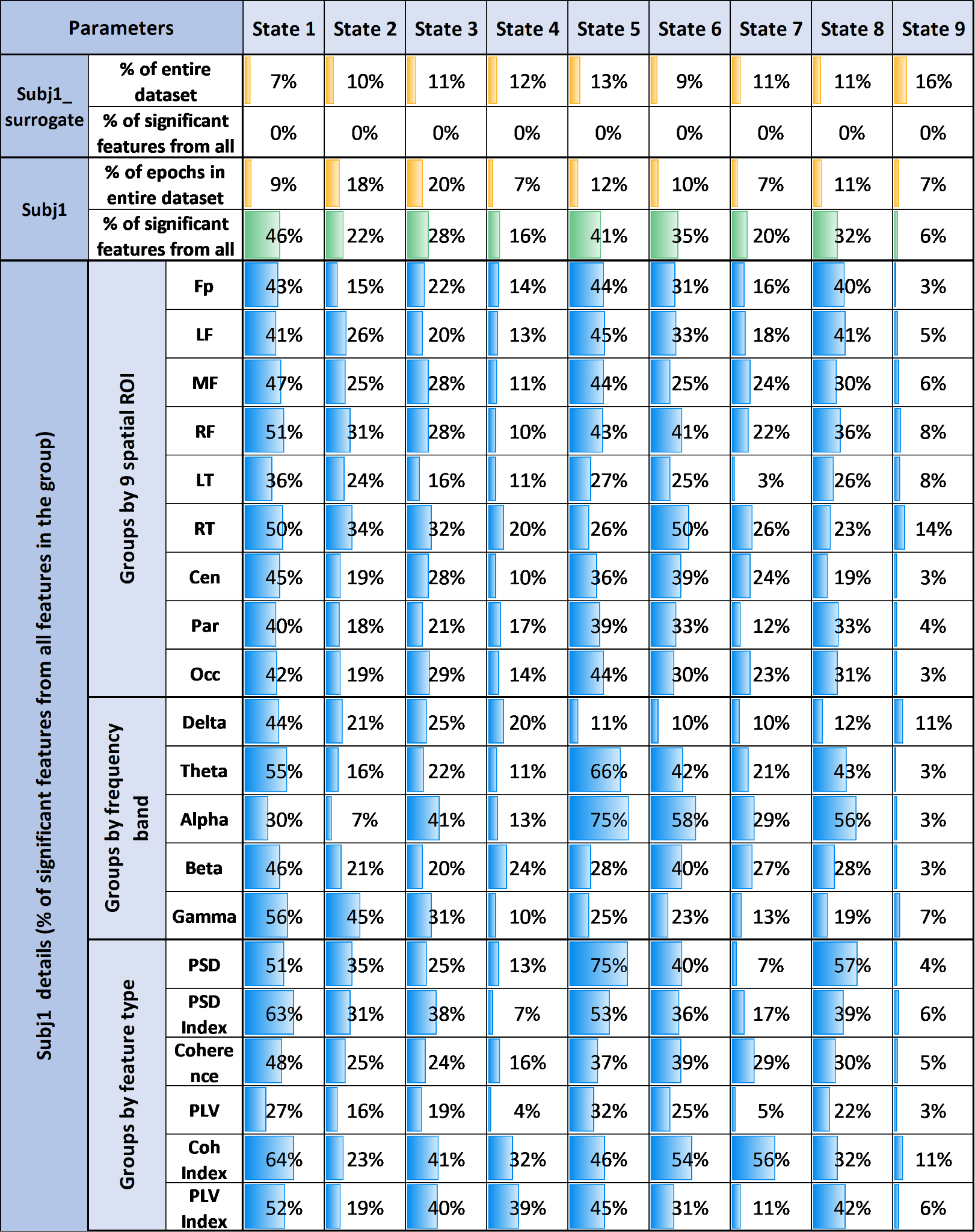 |

| ***Supplementary Table 13****. Statistical significance of features in states versus feature general median value for Subj2 in comparison with Subj2_surrogate. Detailed statistical significance in different groups of features for Subj2. For each state a percent of features, significantly different from median value, is given according to one-sample Wilcoxon signed-rank test with Bonferroni correction (p<0.01).* |
| --- |
| 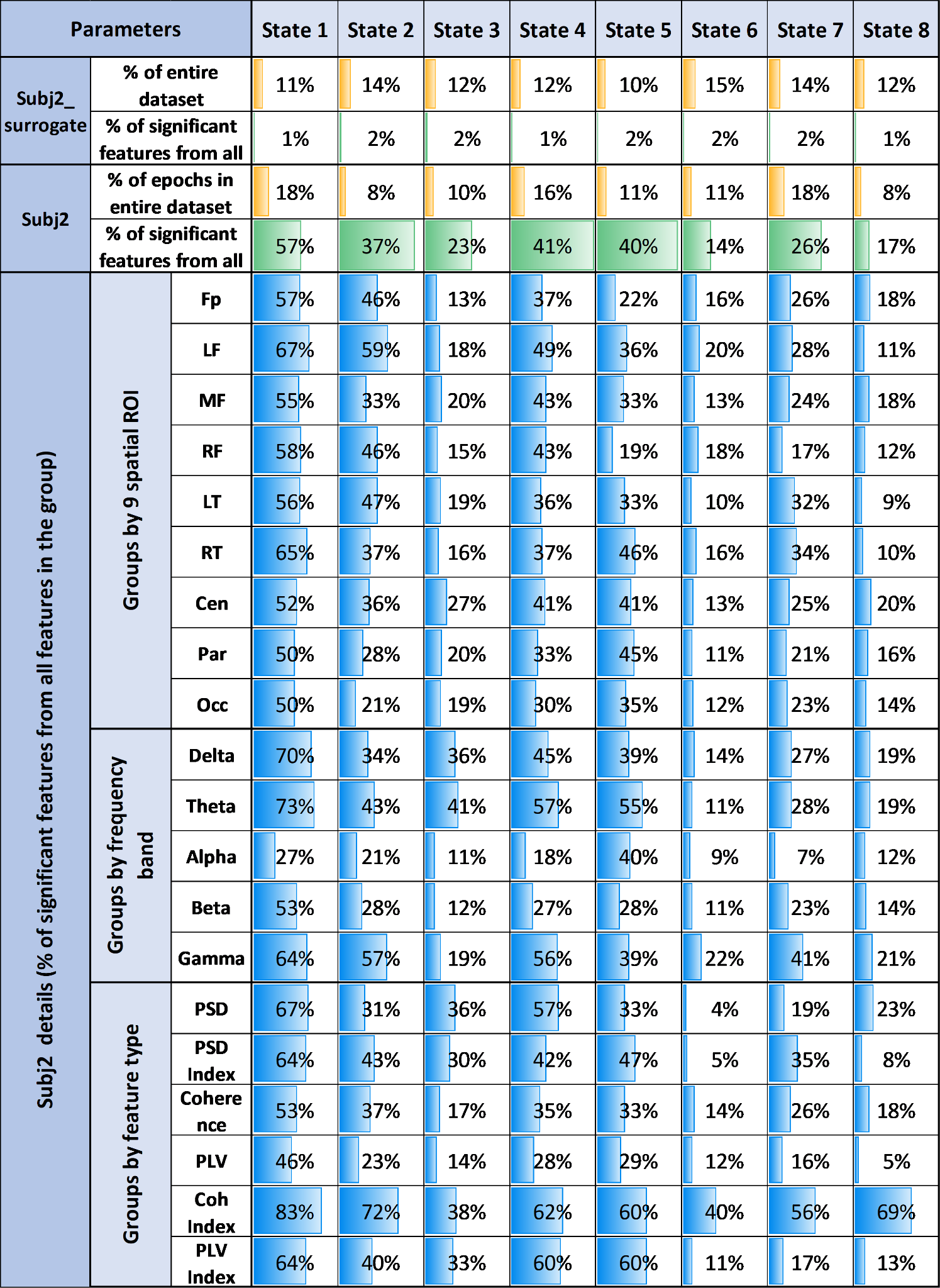 |

| ***Supplementary Table 14****. Statistical significance of features in states versus feature general median value for Subj3 in comparison with Subj3_surrogate. Detailed statistical significance in different groups of features for Subj3. For each state a percent of features, significantly different from median value, is given according to one-sample Wilcoxon signed-rank test with Bonferroni correction (p<0.01).* |
| --- |
| 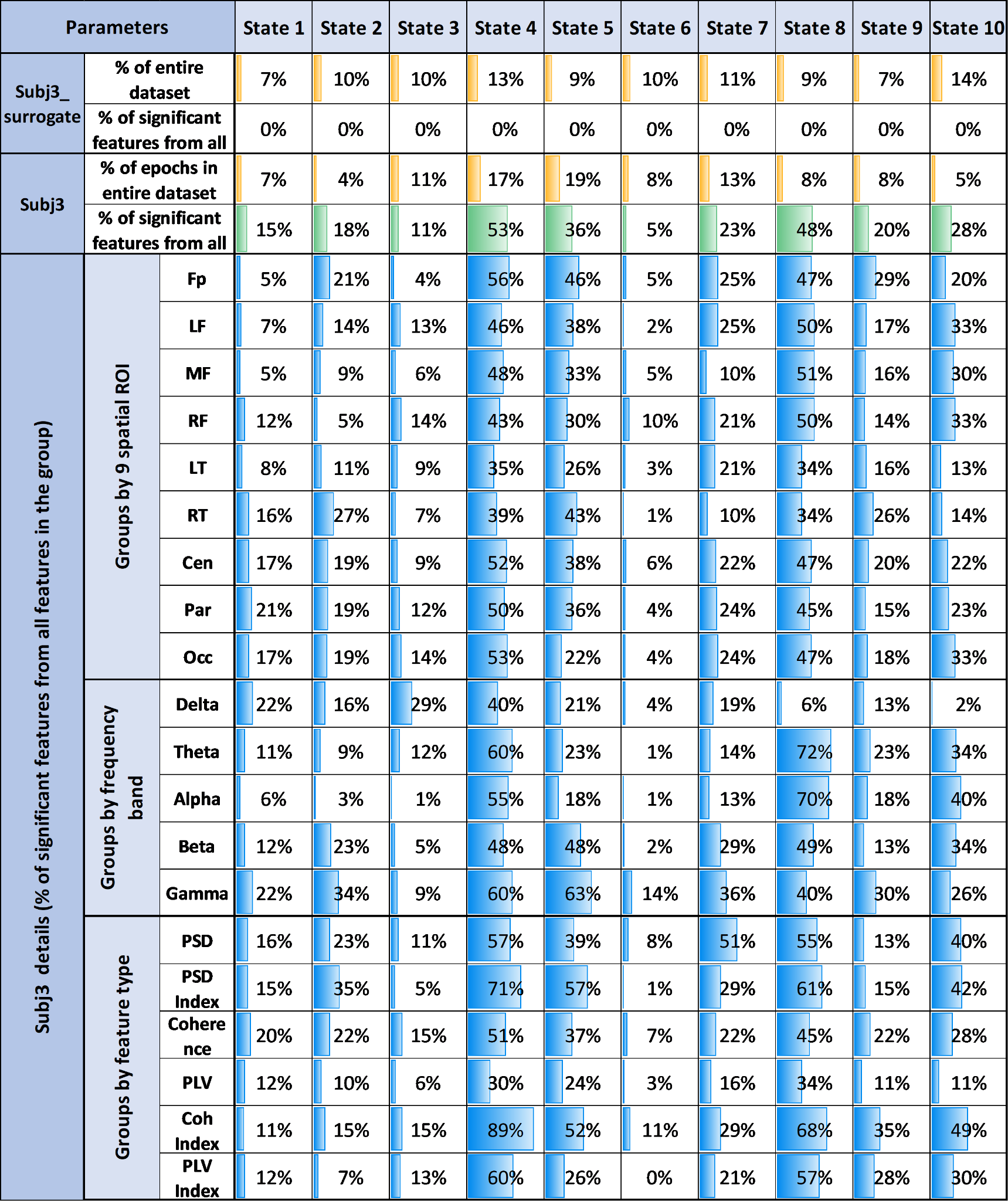 |

| 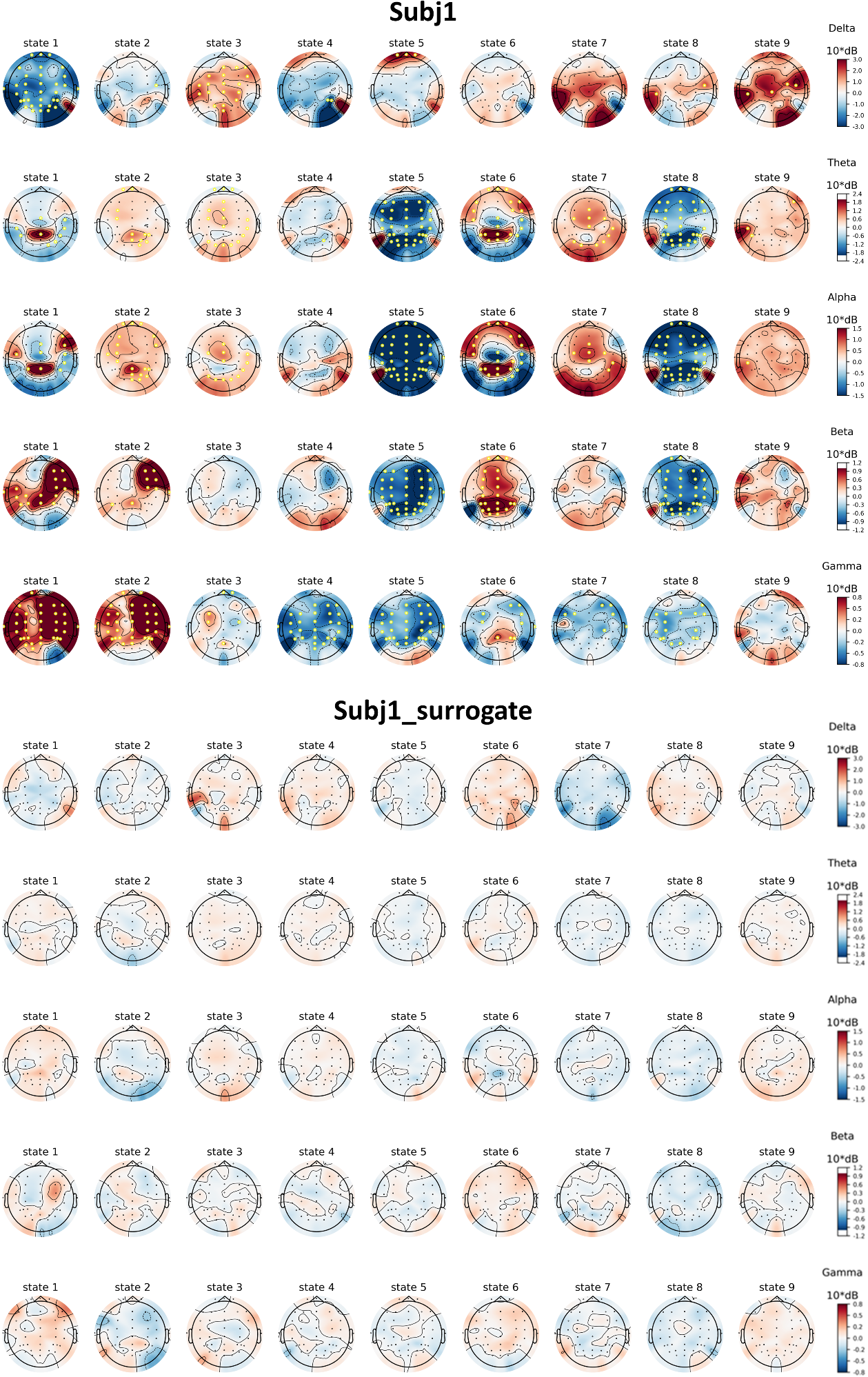 |
| --- |
| **Supplementary Figure 18.** Topographic maps of PSD features for Subj1 and Subj1_surrogate. Refer to Figure 8 caption. |

| 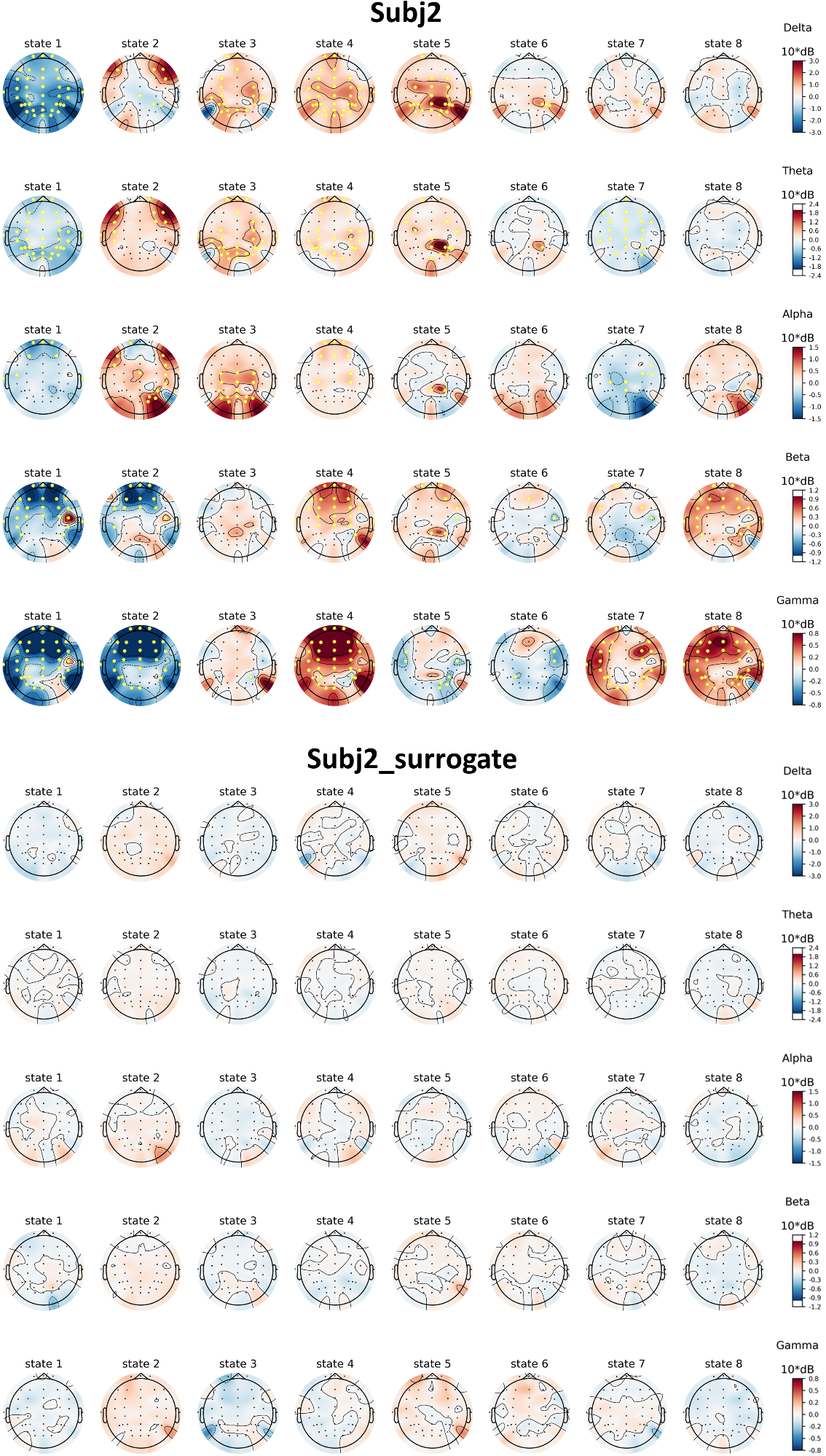 |
| --- |
| **Supplementary Figure 19.** Topographic maps of PSD features for Subj2 and Subj2_surrogate. Refer to Figure 8 caption. |

| 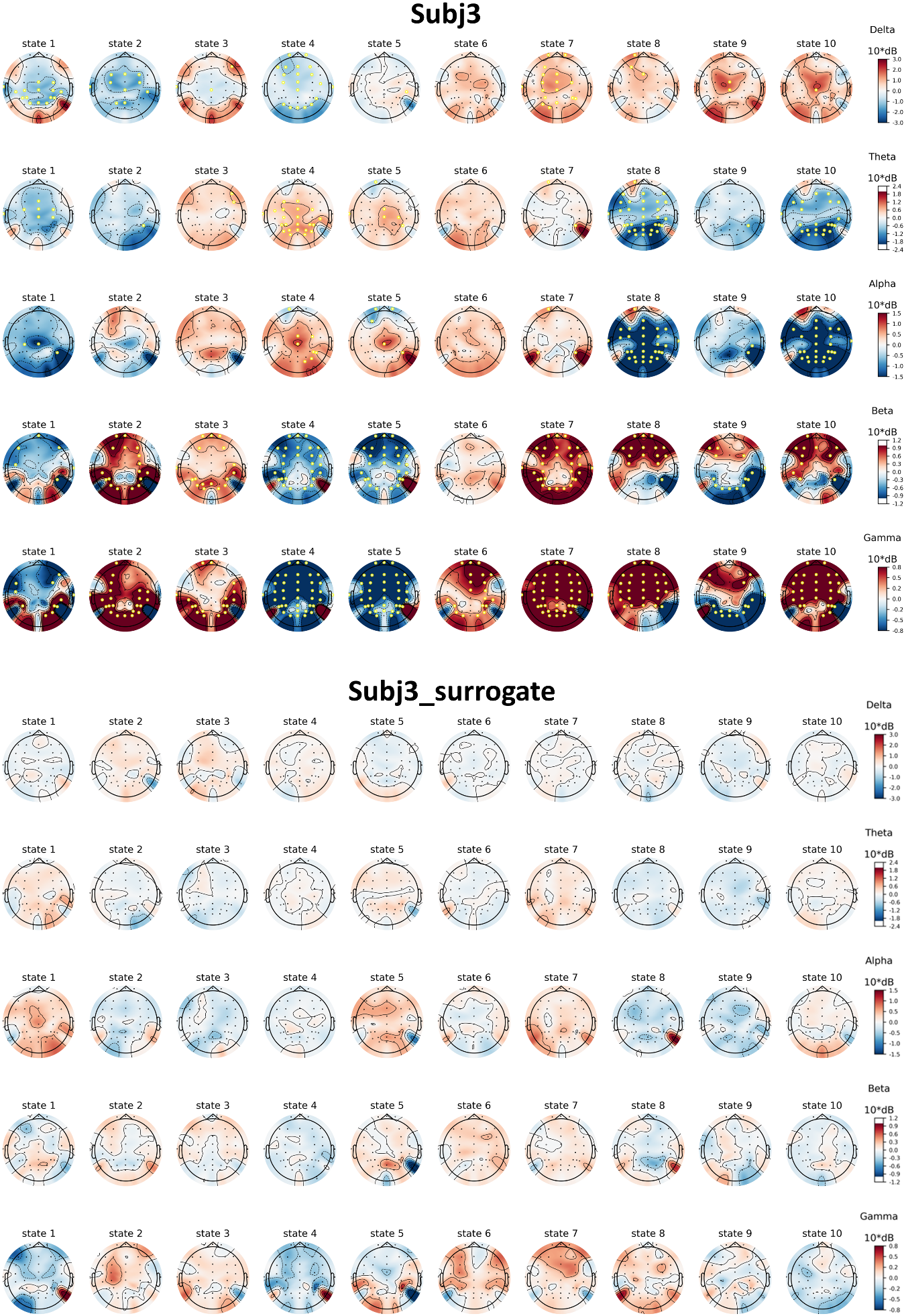 |
| --- |
| **Supplementary Figure 20.** Topographic maps of PSD features for Subj3 and Subj3_surrogate. Refer to Figure 8 caption. |

| ***Supplementary Table 15****. Information value analysis for Subj1 in comparison with Subj1_surrogate in states, obtained by SDA. Detailed IV statistics in different groups of features for Subj1. For each state a percent of features with IV ≥ 0.4 (corresponding to median and strong predictive power of features) is given.* |
| --- |
| 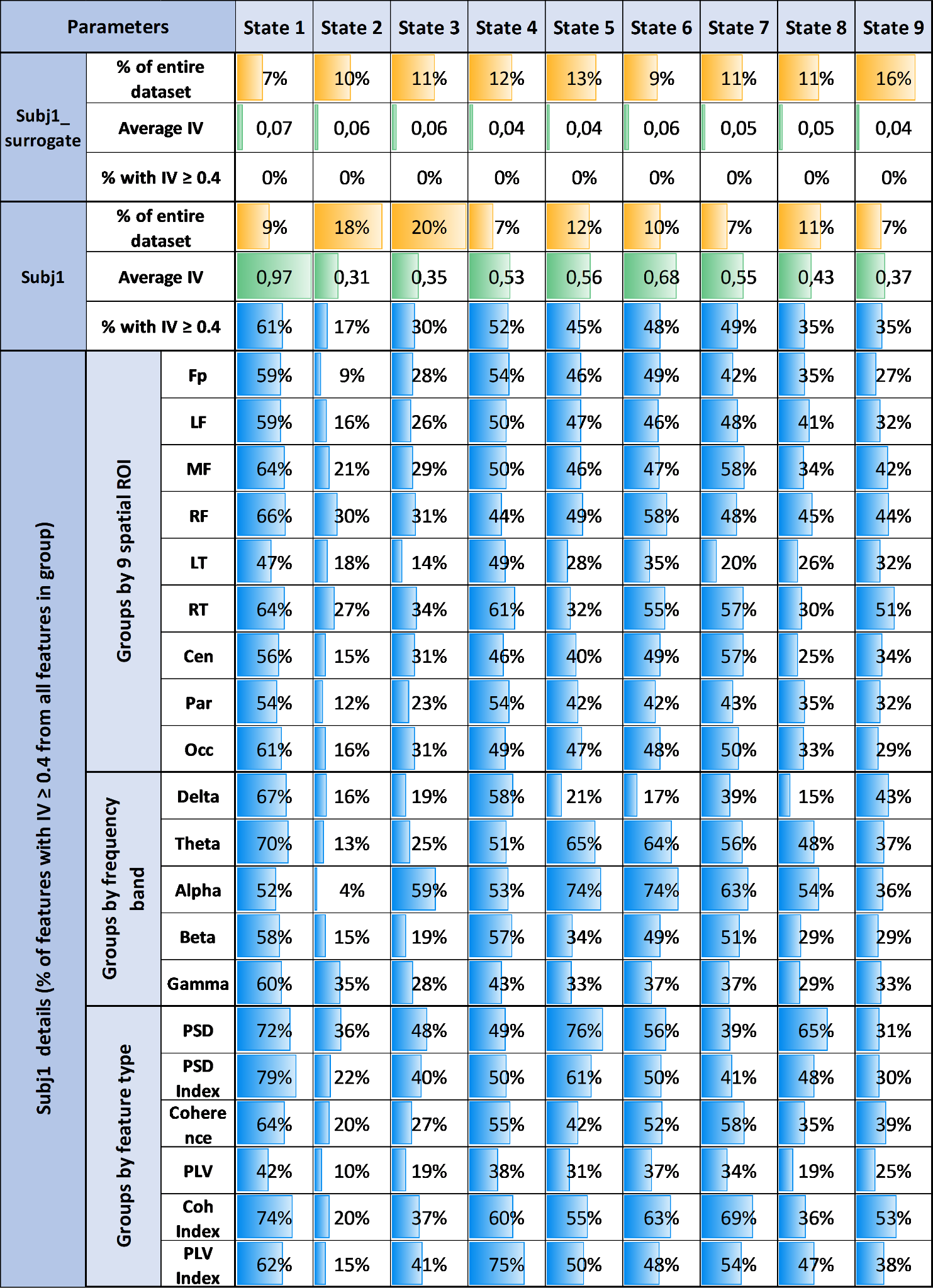 |

| ***Supplementary Table 16****. IV analysis for Subj2 in comparison with Subj2_surrogate in states obtained by SDA. Detailed IV statistics in different groups of features for Subj2. For each state a percent of features with IV ≥ 0.4 (corresponding to median and strong predictive power of features) is given.* |
| --- |
| 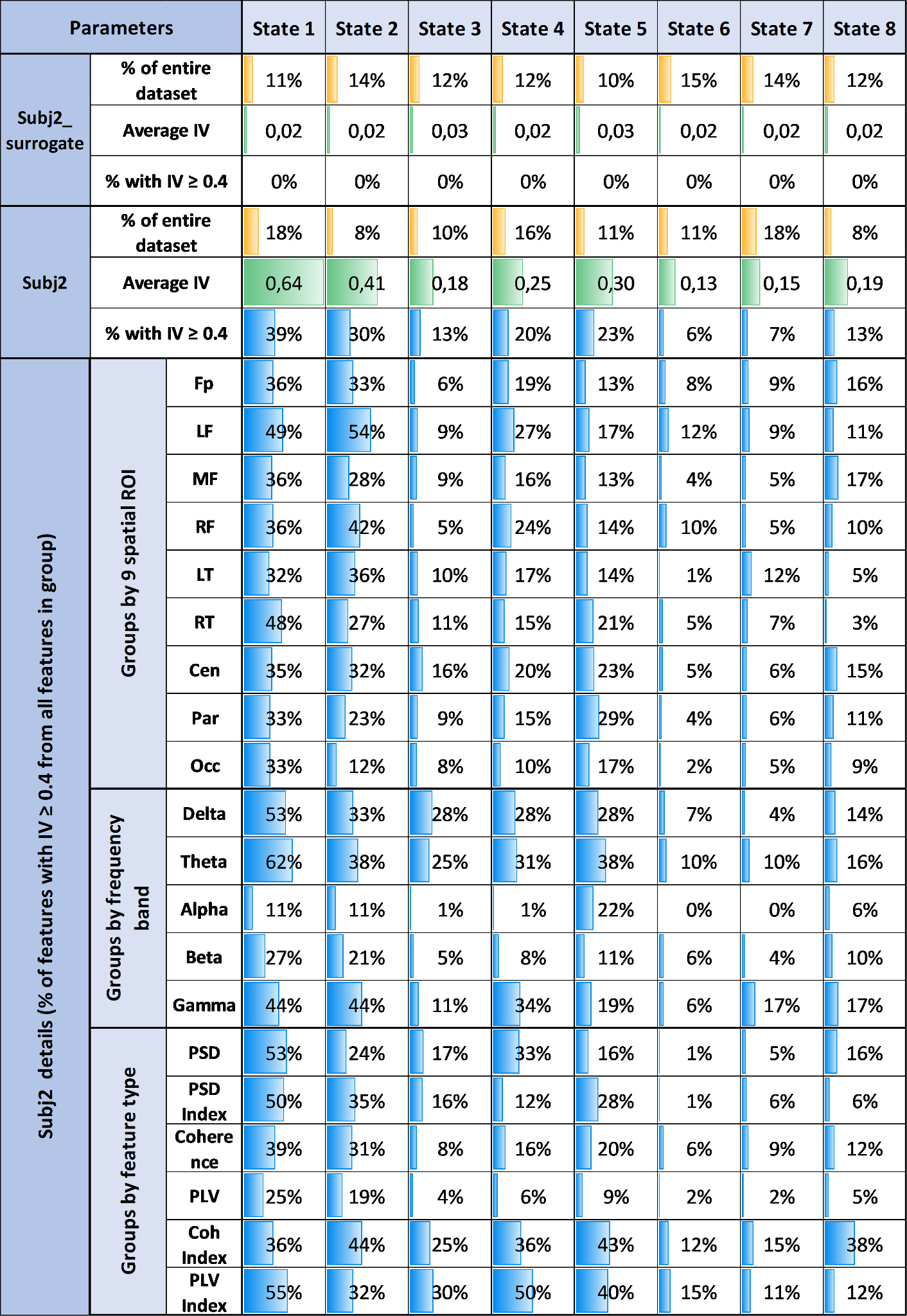 |

| ***Supplementary Table 17****. Information value analysis for Subj3 in comparison with Subj3_surrogate in states, obtained by SDA. Detailed IV statistics in different groups of features for Subj3. For each state a percent of features with IV ≥ 0.4 (corresponding to median and strong predictive power of features) is given.* |
| --- |
| 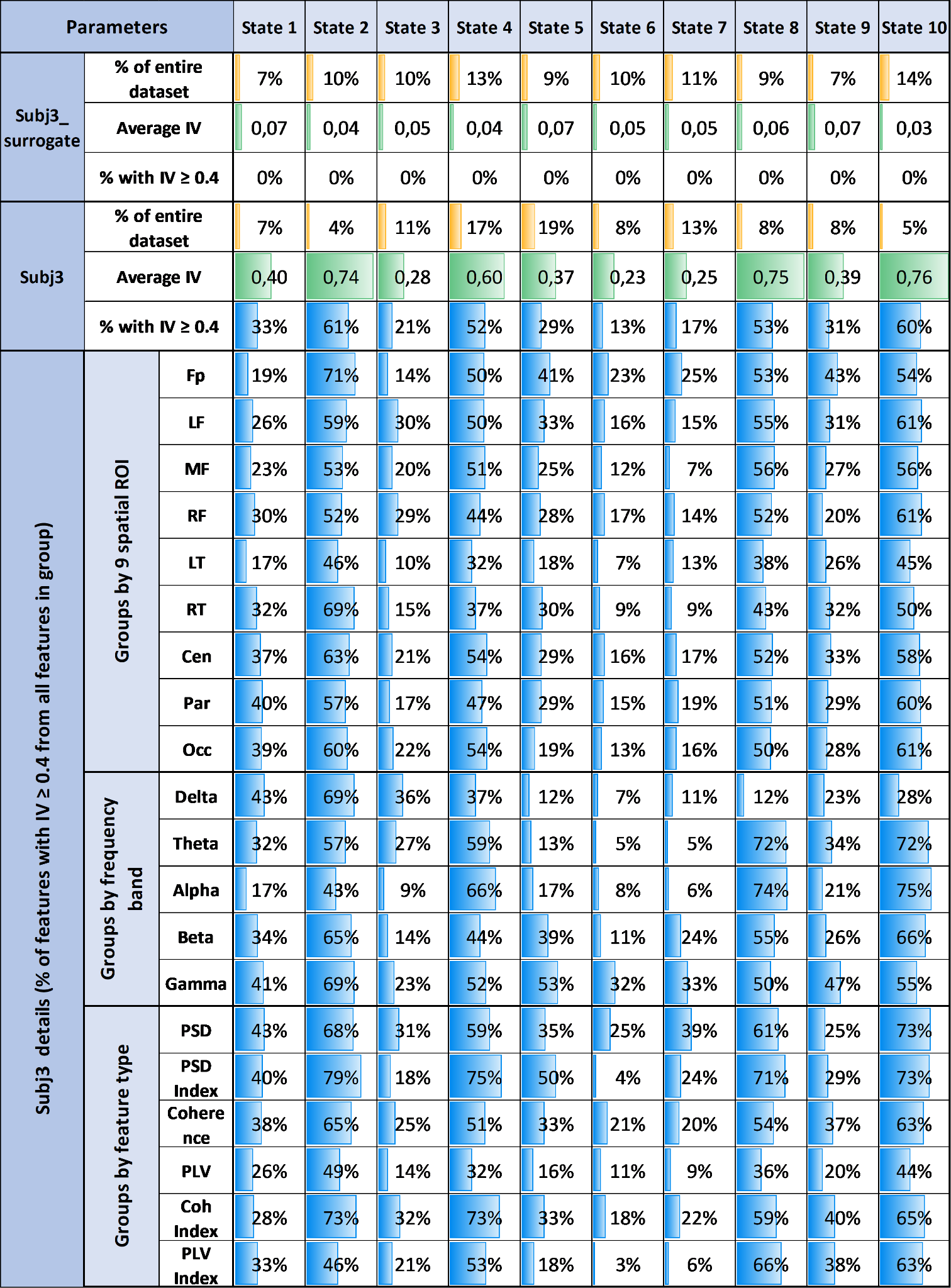 |

| 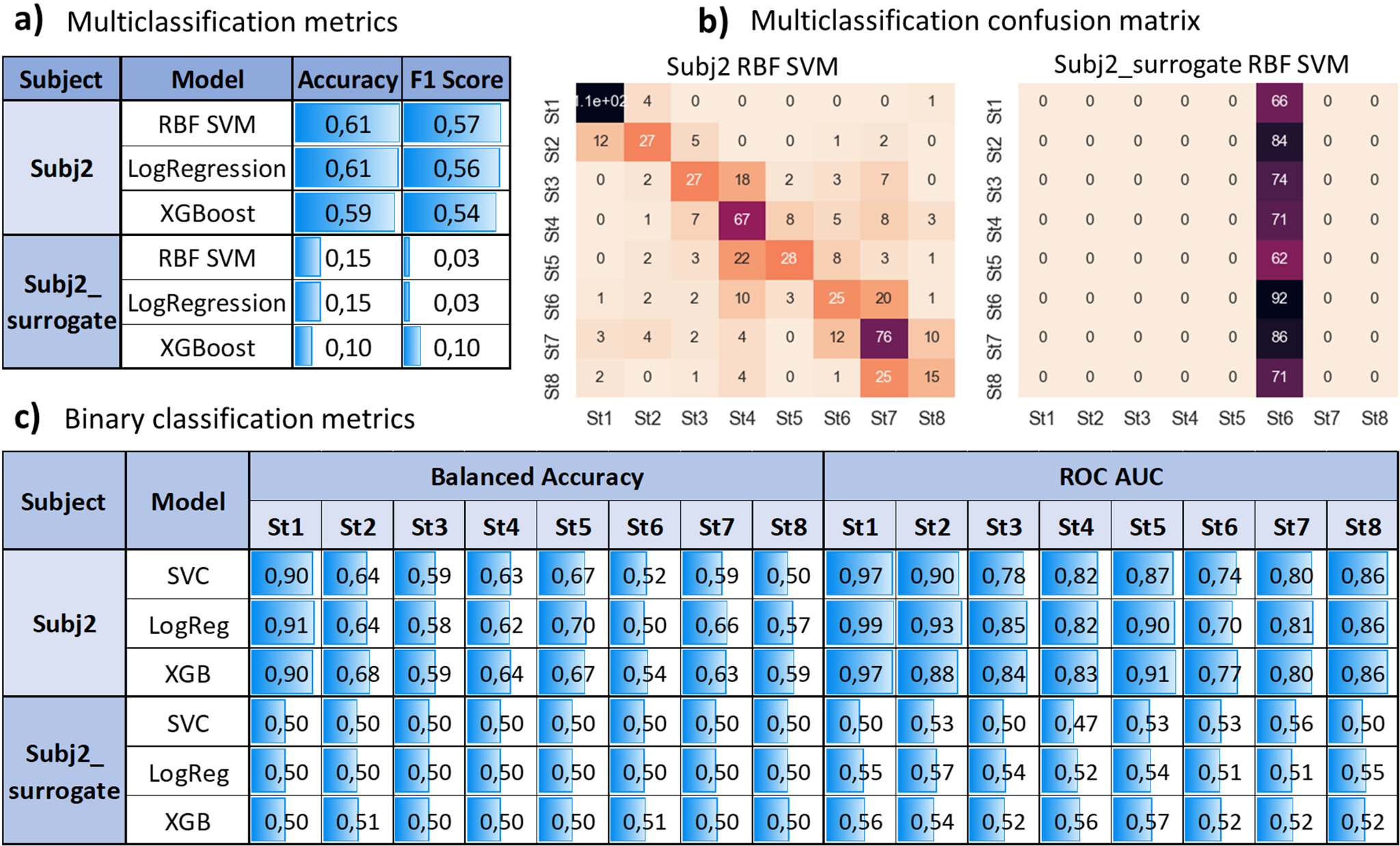 |
| --- |
| **Supplementary Figure 21.** Predictive modeling analysis for Subj2 in comparison with Subj2_surrogate. Refer to Figure 9 caption. |

| 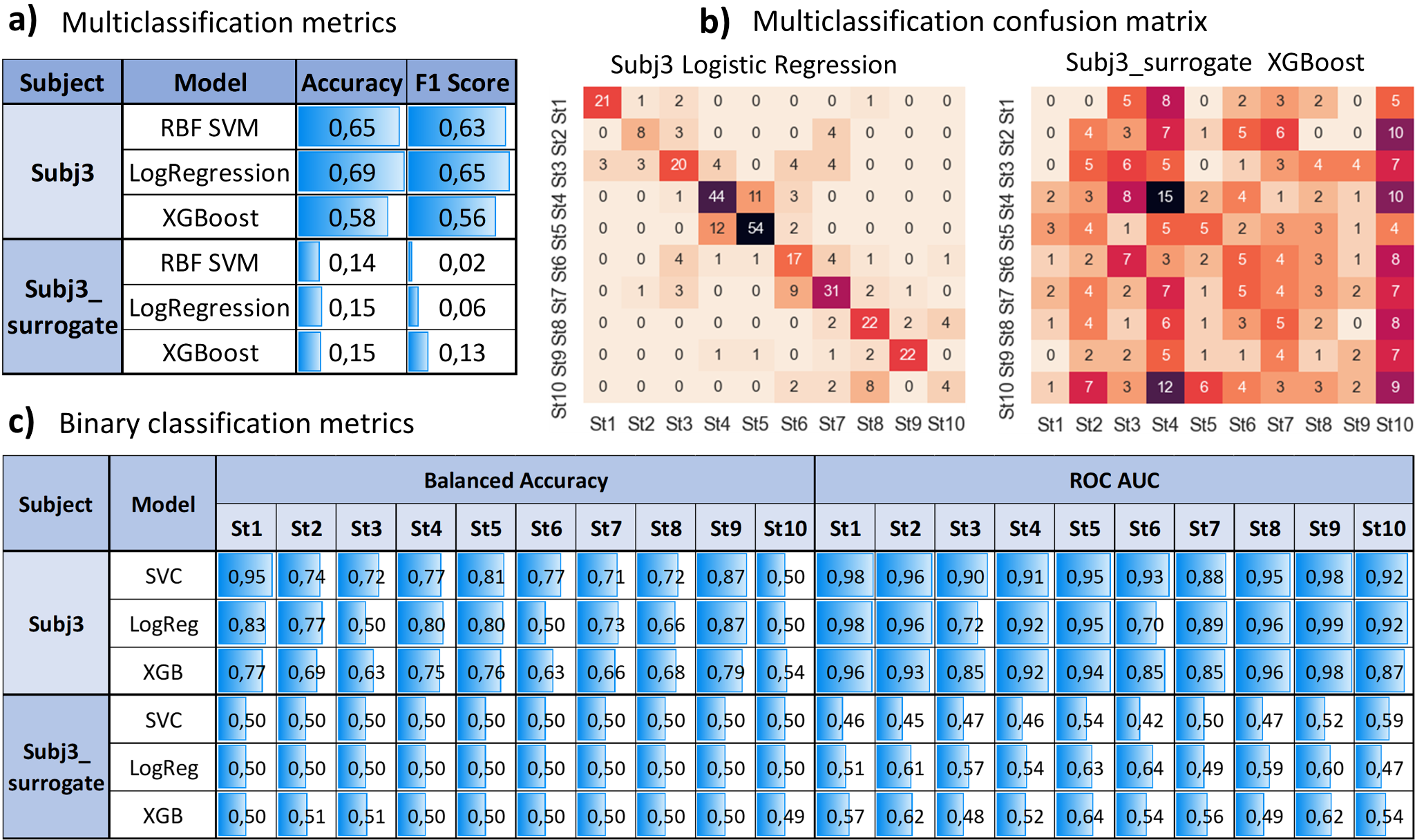 |
| --- |
| **Supplementary Figure 22.** Predictive modeling analysis for Subj3 in comparison with Subj3_surrogate. Refer to Figure 9 caption. |

| 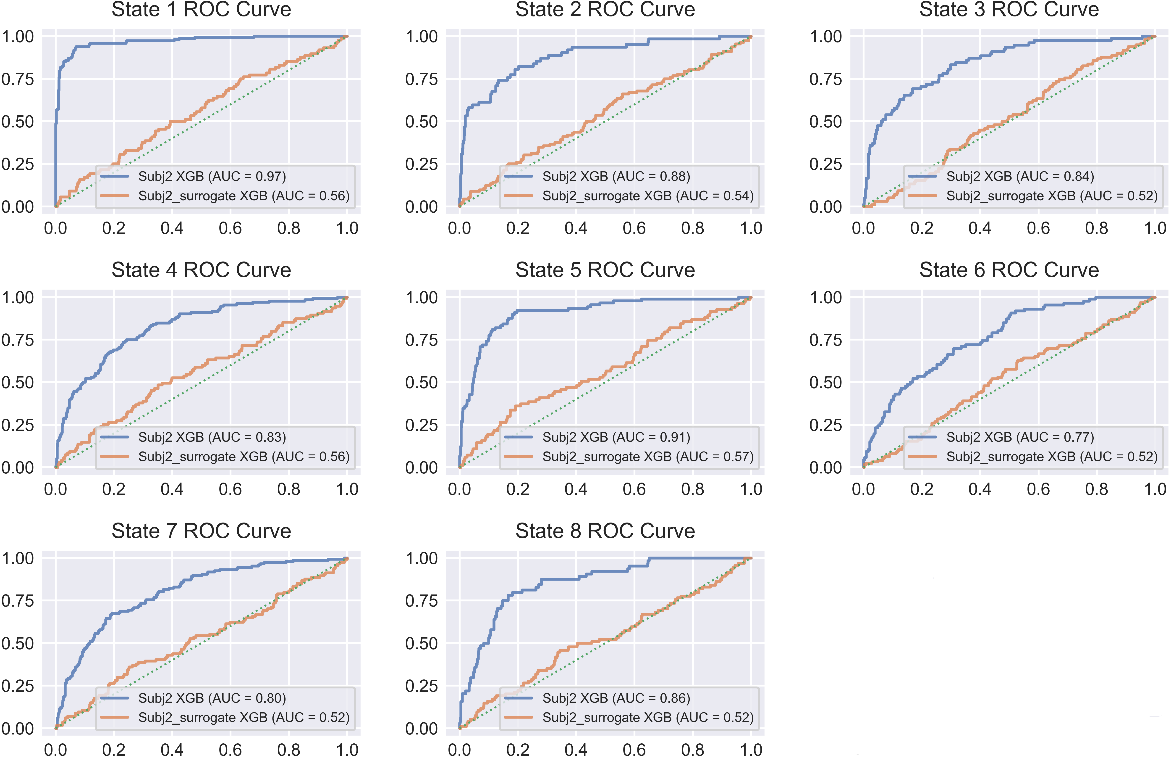 |
| --- |
| **Supplementary Figure 23.** ROC curve plots for binary classifiers, predicting states obtained by SDA for Subj2 and Subj2_surrogate. Refer to Figure 10 caption. |

| 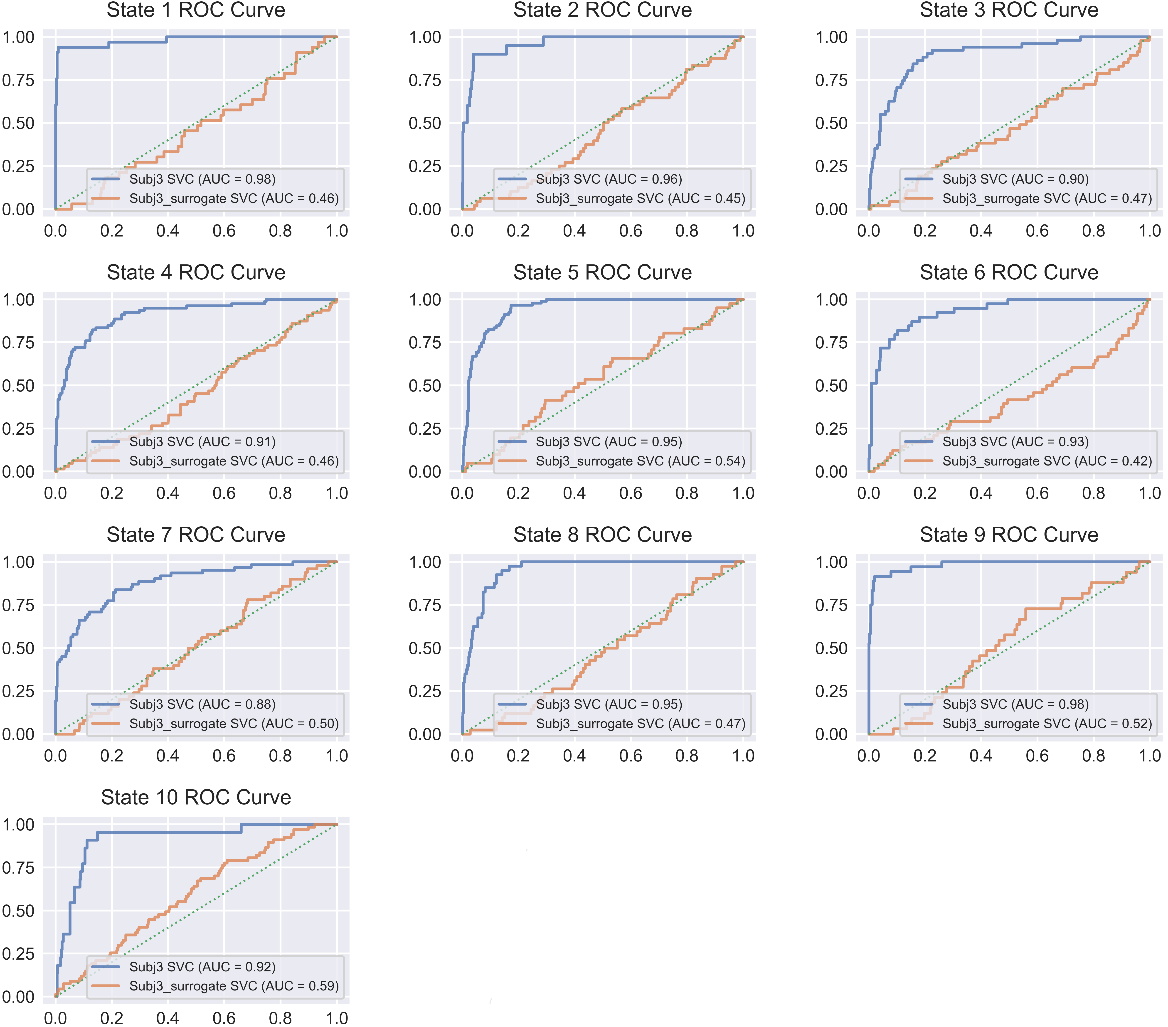 |
| --- |
| **Supplementary Figure 24.** ROC curve plots for binary classifiers, predicting states obtained by SDA for Subj3 and Subj3_surrogate. Refer to Figure 10 caption. |
